# Chromosome-level genome assemblies of four wild peach species provide insights into genome evolution and genetic basis of stress resistance

**DOI:** 10.1186/s12915-022-01342-y

**Published:** 2022-06-13

**Authors:** Ke Cao, Zhen Peng, Xing Zhao, Yong Li, Kuozhan Liu, Pere Arus, Weichao Fang, Changwen Chen, Xinwei Wang, Jinlong Wu, Zhangjun Fei, Lirong Wang

**Affiliations:** 1 The Key Laboratory of Biology and Genetic Improvement of Horticultural Crops (Fruit Tree Breeding Technology), Ministry of Agriculture, Zhengzhou Fruit Research Institute, Chinese Academy of Agricultural Sciences, Zhengzhou, 450009 People’s Republic of China; 2 Novogene Bioinformatics Institute, Beijing, People’s Republic of China; 3 IRTA, Centre de Recerca en Agrigenòmica, CSIC-IRTA-UAB-UB, Campus UAB – Edifici CRAG, Cerdanyola del Vallès (Bellaterra), Barcelona, Spain; 4 Boyce Thompson Institute for Plant Research, Cornell University, Ithaca, NY 14853 USA; 5 National Horticulture Germplasm Resources Center, Zhengzhou Fruit Research Institute, Chinese Academy of Agricultural Sciences, Zhengzhou, 450009 People’s Republic of China

**Keywords:** Comparative genomics, Stress resistance, High-altitude adaptation, Peach

## Abstract

**Background:**

Peach (*Prunus* persica) is an economically important stone fruit crop in Rosaceae and widely cultivated in temperate and subtropical regions, emerging as an excellent material to study the interaction between plant and environment. During its genus, there are four wild species of peach, all living in harsh environments. For example, one of the wild species, *P. mira*, originates from the Qinghai-Tibet Plateau (QTP) and exhibits strong cold/ultraviolet ray environmental adaptations. Although remarkable progresses in the gene discovery of fruit quality-related traits in peach using previous assembled genome were obtained, genomic basis of the response of these wild species to different geographical environments remains unclear.

**Results:**

To uncover key genes regulating adaptability in different species and analyze the role of genetic variations in resistance formation, we performed de novo genome assembling of four wild relatives of peach (*P. persica*), *P. mira*, *P. davidiana*, *P. kansuensis*, and *P. ferganensis* and resequenced 175 peach varieties. The phylogenetic tree showed that the divergence time of *P. mira* and other wild relatives of peach was 11.5 million years ago, which was consistent with the drastic crustal movement of QTP. Abundant genetic variations were identified in four wild species when compared to *P. persica*, and the results showed that plant-pathogen interaction pathways were enriched in genes containing small insertions and deletions and copy number variations in all four wild relatives of peach. Then, the data were used to identify new genes and variations regulating resistance. For example, presence/absence variations which result from a hybridization event that occurred between *P. mira* and *P. dulcis* enhanced the resistance of their putative hybrid, *P. davidiana*. Using bulked segregant analysis, we located the nematode resistance locus of *P. kansuensis* in chromosome 2. Within the mapping region, a deletion in the promoter of one *NBS-LRR* gene was found to involve the resistance by regulating gene expression. Furthermore, combined with RNA-seq and selective sweeps analysis, we proposed that a deletion in the promoter of one *CBF* gene was essential for high-altitude adaptation of *P. mira* through increasing its resistance to low temperature.

**Conclusions:**

In general, the reference genomes assembled in the study facilitate our understanding of resistance mechanism of perennial fruit crops, and provide valuable resources for future breeding and improvement.

**Supplementary Information:**

The online version contains supplementary material available at 10.1186/s12915-022-01342-y.

## Background

Peach (*Prunus persica*) is the third most important of the deciduous fruit crop in the world, and is widely cultivated in temperate and subtropical regions, ranking only after apple and pear. Benefiting from the small genome size, peach has been extensively used for comparative and functional genomic researches of the Rosaceae family [1]. Nine years ago, the first high-quality reference genome sequence of peach was released [2]. Subsequently, the evolution [3, 4], domestication regions [3, 5, 6], and genes associated with important traits [7, 8] for peach had been successfully dissected.

Adaptability is the basis of plant survival and reproduction, especially under extreme environmental conditions. Among the four wild relatives of *P. persica*, *P. mira* originate from the Qinghai-Tibet Plateau (QTP) and show strong adaptability to cold and ultraviolet rays (UV) resistance. *P. davidiana* is widely distributed in the slopes and valleys of mountains in northern China and is resistant to cold and drought environments. *P. kansuensis* and *P. ferganensis* grow in one specific region, Gansu and Xinjiang province of China, respectively [9]. However, genomic basis of the response of these wild species to different geographical environments remains unclear.

Genome assembly is a powerful tool to discover elite genes and the method have used frequently in peach in recently years. For example, Guan et al. [10] produced a comprehensive structure variations map for peach to identify a large 1.67-Mb heterozygous inversion that segregates perfectly with flat-fruit shape after assembling a flat-fruit peach cultivar “Rui You Pan 1.” Cao et al. [11] analyzed the molecular basis of the reduction of volatile compounds during the evolution of peaches and identified the key gene regulating linalool content through the assembly of a cultivated peach variety “Chinese Cling” and the association analysis of 256 peach varieties. Moreover, some wild related species of peach were also assembled. Tan et al. [12] reported de novo genome assemblies for five species, including *P. persica*, *P. mira*, and *P. davidiana*. Next, the author performed an association analysis of 417 peach accessions to identify loci associated with fruit shape, fruit development period, and floral morphology, respectively. In general, researchers mainly focused on gene identification of fruit quality characters in these studies. To dissect the genetic basis of adaptation to high plateau of *P. mira*, Wang et al. [13] de novo assembled three high-quality genomes of Tibetan *Prunus* species, including *P. mira*. The results showed that the expansion of SINE retrotransposons helped Tibetan *Prunus* species adapt to the harsh environment of the Himalayan plateau by promoting the accumulation of beneficial metabolites.

In this study, to dissect the mechanism by which regulate peach evolution and species adaptation at the genome-wide level, we de novo assembled the genomes of four wild relatives of *P. persica*. Comparative genomic analysis showed that *P. davidiana* was evolved from *P. mira* directly and crossed *P. dulcis* during its evolutionary history. Then, a nematode resistance gene was identified in *P. kansuensis* using the identified genetic variations based on new assemblies. Finally, comprehensive characterization of selective sweeps revealed the new mechanisms underlying the high-altitude adaptability in *P. mira*. Compared with previous studies, the genomes of four wild species assembled in the study are of higher quality, providing more detailed basic data for other researchers to study peach evolution and dissect the genetic mechanism of resistance traits.

## Results

### Assembly of the genomes of four wild peach species

The high-quality genome of *P. mira*, *P. davidiana*, *P. kansuensis*, and *P. ferganensis* were assembled using a combination of PacBio, Illumina, and Hi-C (High-throughput chromosome conformation capture) platforms. Using the k-mer method (Additional file 1: Table S1; Additional file 2: Fig. S1), we estimated the size of four genomes to be 242.94, 237.29, 238.06, and 237.24 with a heterozygous ratio of 0.76%, 1.10%, 0.56%, and 0.53% in *P. mira*, *P. davidiana*, *P. kansuensis*, and *P. ferganensis*, respectively. Then, a total of 600.37 ×, 574.84 ×, 343.99 ×, and 387.90 × coverage of sequences were generated for above species and used for genome assembly, respectively (Additional file 1: Table S2). Among the ultimately obtained 253.63, 259.27, 253.17, and 261.3 Mb assemblies (Table 1; Fig. S2), 92.29%, 89.46%, 94.03%, and 90.53% of them were anchored and allocated to eight pseudochromosomes for *P. mira*, *P. davidiana*, *P. kansuensis*, and *P. ferganensis*, respectively (Additional file 1: Table S3). The contig N50 sizes of the four final assemblies were 25.95, 22.64, 24.42, and 26.47 Mb, respectively (Table 1), which were higher than that of *P. persica* (250 kb), which produced by the Sanger technology [14]. Compared with the previously published genomes of *P. mira* and *P. davidiana* with contig N50 of ~ 8.3 Mb and 2.3 Mb, respectively [12], our final assembly showed an ~ 3.1 and 10.0-fold increase in the length of the contig N50. In addition, compared with another released *P. mira* assembly with a contig N50 of 12.14 Mb [13], our *P. mira* assembly showed an ~ 2.14-fold increase in the length of the contig N50. We also performed Benchmarking Universal Single-Copy Orthologs (BUSCO) analysis [15] (Additional file 1: Table S4) and RNA-Seq read mapping rate calculation (Additional file 1: Table S5) to evaluate the quality of the assembly. The results indicated that more than 95% of the highly conserved embryophyta genes were completely present in the four genomes, and more than 85% RNA-Seq reads could be mapped to the corresponding genomes. Subsequently, repeat sequence were annotated and the results showed that long terminal repeat (LTR) retrotransposons made up the majority of the transposable elements (TEs), comprising 35.61%, 42.44%, 41.75%, and 41.86% of the *P. mira*, *P. davidiana*, *P. kansuensis*, and *P. ferganensis* genome, respectively (Additional file 1: Table S6). Gene prediction and annotation were performed resulting in 28,519, 27,236, 26,986, and 28,587 protein-coding genes in *P. mira*, *P. davidiana*, *P. kansuensis*, and *P. ferganensis*, respectively (Additional file 2: Fig. S2; Additional file 1: Table S7 and 8). About 96.82-97.73% of the protein-coding genes of the four wild species could be functionally annotated (Additional file 1: Table S9). In addition, we also identified 8171-15,770 ribosomal RNA, 1226-1564 transfer RNA, 514-753 small nuclear RNA, and 563-693 microRNA genes in the four wild peach species (Additional file 1: Table S10).

**Table 1:** Genomic sequencing, assembly, and annotation statistic for four wild peach species

Species	***P. mira***	***P. davidiana***	***P. kansuensis***	***P. ferganensis***
**Estimated genome size (Mb)**	242.94	237.29	238.06	237.24
**Sequencing depth (×)**	600.37	574.84	343.99	387.9
**Total sequencing data (Gb)**	145.86	136.41	91.15	92.02
**Assembled genome size (Mb)**	253.63	259.27	253.17	261.30
**Contig N50 (bp)**	25,948,270	22,637,438	24,419,080	26,470,137
**Contig N50 (number)**	4	4	5	4
**Scaffold N50 (bp)**	27,933,902	28,549,251	28,885,292	28,247,033
**Scaffold N50 (number)**	4	4	4	4
**GC content (%)**	38.29	38.92	38.34	38.84
**Percent of repeat sequence (%)**	48.38	52.27	49.33	49.49
**Predicted gene number**	28,519	27,236	26,986	28,587
**Annotated gene number**	27,613	26,545	26,374	27,803

**Fig. 1.**
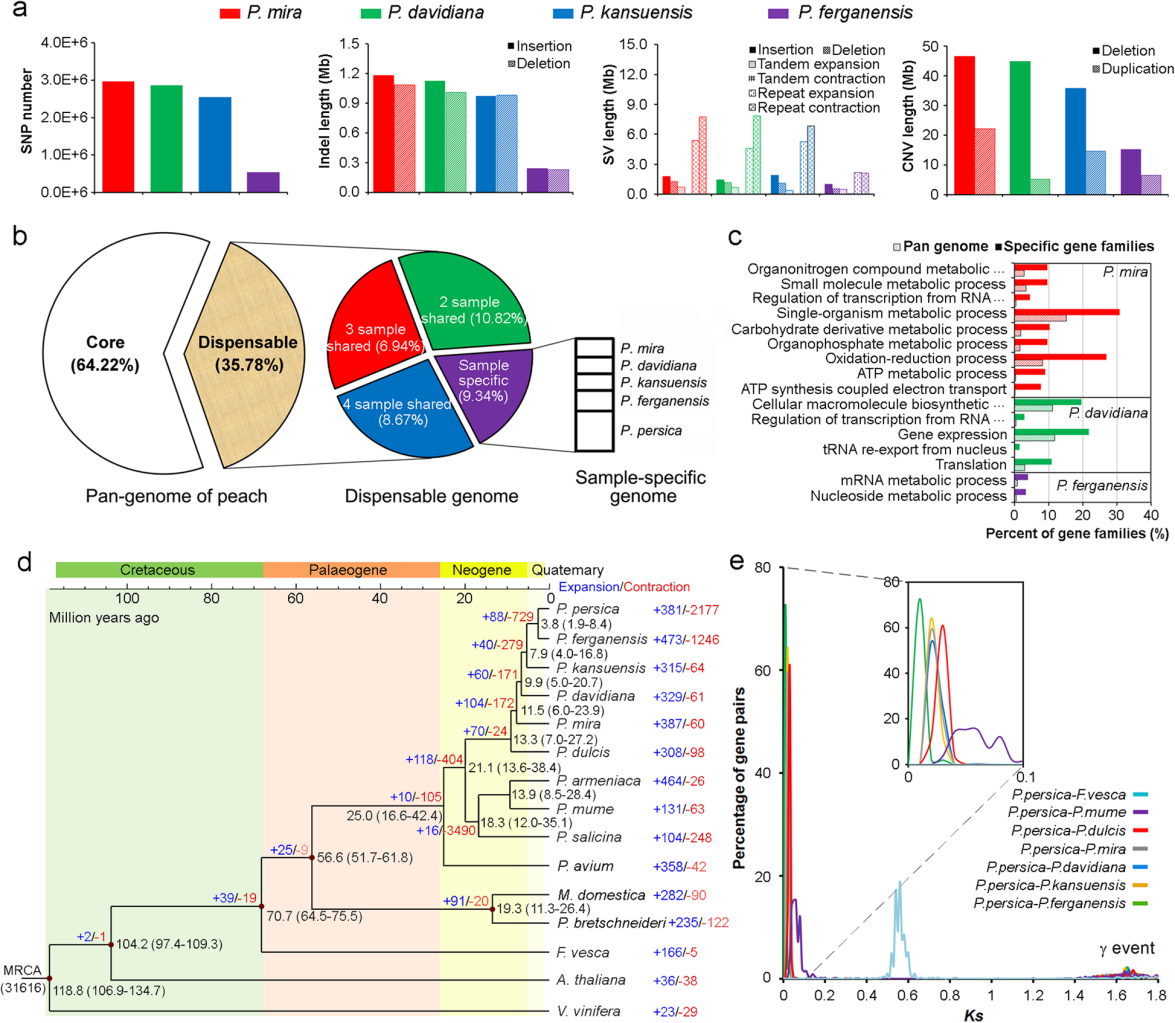
Variation screening of four wild species and evolutionary analysis of peach genome. **a** SNPs, small indels, SVs, and CNVs in *P. mira*, *P. davidiana*, *P. kansuensis*, and *P. ferganensis* compared to *P. persica*. **b** Core and dispensable gene families of four wild peaches and *P. persica*. **c** Gene Ontology annotation of genes specific in each species. **d** Estimation of divergence times of 15 species and identification of gene family expansions and contractions. Numbers on the nodes represent the divergence times from present (million years ago, Mya). MCRA, most recent common ancestor. **e** Distribution of *Ks* (synonymous mutation rate) values of orthologous genes between six genomes of the *Prunus* species and strawberry

Genome variations between species were also identified by anchoring the four assembled wild genomes onto the reference genome of *P. persica* [14]. A total of 547,998-2,971,987 single nucleotide polymorphisms (SNPs; Additional file 1: Table S11), 106,436-490,421 small insertions and deletions (indels; Additional file 1: Table S12), 3870-10,994 large structural variants (SVs; ≥ 50 bp in length; Additional file 1: Table S13), and 3344-6840 copy number variations (CNVs; Additional file 1: Table S14) were identified in the four species (Fig. 1a; Additional file 2: Fig. S3). A total of 20 SVs that differed in *P. mira* and *P. persica* was randomly selected (Additional file 1: Table S15) to design primers (Additional file 1: Table S16) for amplification in cultivar “2010-138” and “Shenzhou Li He Shui Mi” to verify the accuracy of these variations. The results showed that among the 20 variants, only the electrophoresis results of two primers might be inconsistent with the sequencing results (Additional file 2: Fig. S4). Meanwhile, IGV software (www.broadinstitute.org/igv) was used to observe the above 20 variant sites manually. We analyzed the function of the genes which comprised different variations (Additional file 2: Fig. S5-8) and found that plant-pathogen interaction pathways were enriched in genes containing small indels and CNVs in all four wild relatives of peach (*p* = 0.01 ~ 1.74e^−22^), showing that responding with environmental stress was very important for the speciation of four species.

### Peach genome evolution and species divergence

Combining the genome of *P. mira*, *P. davidiana*, *P. kansuensis*, *P. ferganensis*, and *P. persica*, all of the genes in the genomes of these five peach species were classified into 22,747 families on the basis of the homology of their encoded proteins (Additional file 2: Fig. S9). Presence/absence variations (PAVs) analysis of gene families revealed 8,138 (35.78%) dispensable gene-families distributed across all five genomes, and 368, 303, 303, 388, and 763 families specific to each of the above species, respectively (Fig. 1b). Single-organism metabolic process (*p* = 1.36e^−6^) and oxidation-reduction (*p* = 2.53e^−15^) were found to be enriched in gene families specific to *P. mira*. Cellular macromolecule biosynthetic process (*p* = 0.05) and gene expression (*p* = 0.01) pathways were enriched in specific gene families of *P. davidiana* (Fig. 1c).

A total of 830 single-copy gene families in the 15 species were identified (Additional file 2: Fig. S10) and used to construct a phylogenetic tree of *P. persica* and its wild related species as well as other representative plant species (Fig. 1d). Based on the known divergence time between *Arabidopsis thaliana* and strawberry, the age estimate for the split of the ancestor of *P. mume*, *P. armeniaca*, *P. salicina*, and the common ancestor of peach and almond was around 21.1 million years ago (Mya), later than that in a previous report, presumably 44.0 Mya [16]. Hereafter, the divergence time of *P. dulcis* and *P. mira* was about 13.3 Mya, which was obviously earlier than that of Yu et al. [4] and Alioto et al. [17] who found the divergence time of the two species was 4.99 and 5.88 Mya, respectively. Furthermore, we found that *P. mira* split with the common ancestor of *P. davidiana*, *P. kansuensis*, and *P. ferganensis* approximately 11.5 Mya. The event occurred around the drastic crustal movement of QTP [18] where *P. mira* originated.

Lineage-specific gene family expansions may be associated with the emergence of specific functions and physiology [19]. With the genome evolution of wild peach relatives, the number of expanded gene families was decreased from *P. mira* to *P. kansuensis* while increased in *P. ferganensis* or *P. persica* compared with that in the most recent common ancestor (MCRA; Fig. 1d). We found that the expanded gene families were highly enriched with those related to aminobenzoate degradation (or arginine and proline metabolism, styrene degradation) in *P. mira* (*p* = 5.53e^−21^; Additional file 2: Fig. S11) which originated in QTP, same as in *Polylepis tarapacana* [20] and *Populus kangdingensis* [21] where lived in high altitude regions.

To validate the speciation events of peach species, fourfold synonymous third-codon transversion (4DTv) rates were calculated and the results showed that all 4DTv values (Additional file 2: Fig. S12) among paralogs in four wild peach species peaked at around 0.50 to 0.55, consistent with the whole-genome triplication event (γ event) shared by all eudicots and indicating that no recent whole-genome duplication occurred. A peak 4DTv value at around 0 for the orthologs between four wild related species of peach and *P. persica* highlighted a very recent diversification of *Prunus* species [16]. To estimate the time of species divergence of the four wild species, we calculated the *Ks* (rate of synonymous mutation) values of orthologous genes between these species. As shown in Fig. 1e, the peaks at a *Ks* mode for orthologs between *P. persica*-*P. dulcis* was higher than that of other peaches. In addition, among the wild related species of *P. persica*, the similar *Ks* value of *P. mira*, *P. davidiana*, and *P. kansuensis* showed significantly higher than that of *P. ferganensis*, indicating similar results with phylogenetic tree.

### Variations screening provided identification of new genes regulating resistance

To assess the advantage of genome assemblies of peach using five species, we analyzed the molecular mechanism for regulating the resistance traits of different species one by one. According to previous reports, *P. fergenansis* was recognized as only a geographic type of *P. persica* [3], so it was not discussed in the following analysis. We found that PAV may explain the multi-resistance of *P. davidiana* to diverse stresses and the deletions in the promoter region could enhances the resistance of *P. kansuensis* to nematodes and *P. mira* to low temperature by regulating the expression of target genes, respectively. Specific cases are described below.

### Interspecific hybridization is essential for *P. davidiana* to obtain the multi-resistance traits

*P. davidiana* is a very important wild species of *P. persica* because of its resistance to various abiotic stresses such as drought and cold and biotic stresses such as aphids, which may be of great significance in the use of resistance breeding systems in the future [22]. Here, we first screened the *R* genes from four wild species and found a total of 295, 341, 310, and 322 genes were identified in *P. mira*, *P. davidiana*, *P. kansuensis*, and *P. ferganensis* genomes, respectively (Additional file 1: Table S17). The largest number of *R* genes in *P. davidiana* might explain its strong and multiple resistances to different pathogens, such as aphid, *Agrobacterium tumefaciuns*, etc. We found that most of the disease resistances QTLs/genes were located in genome regions containing candidate *R* genes (Additional file 2: Fig. S13).

**Fig. 2.**
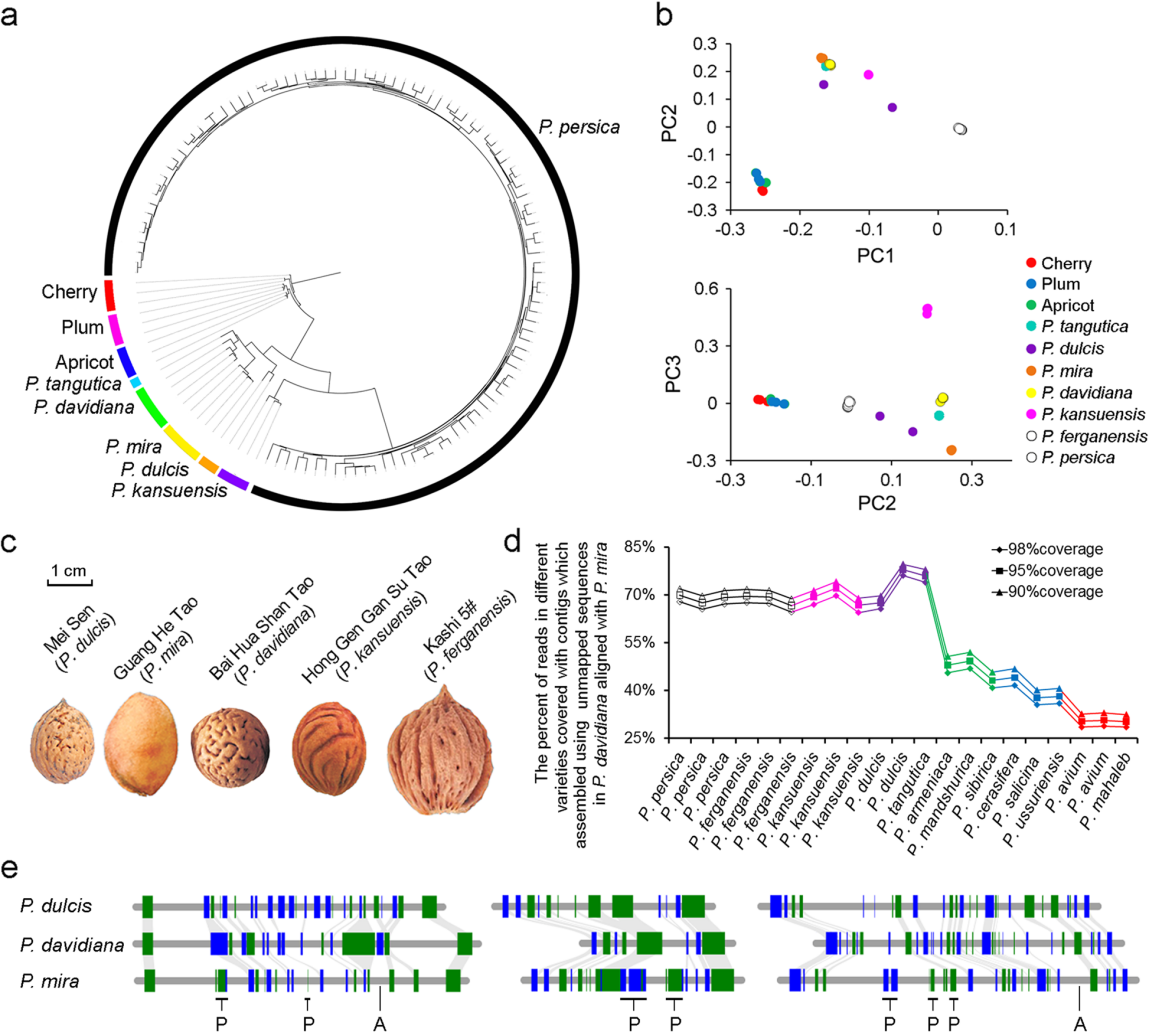
The evolution of *P. davidiana*. **a** Phylogenetic tree of 126 peach accessions which included *P. persica* and its wild relative species. **b** Principal component analysis (PCA) of above accessions. **c** Stone steak morphology of *P. dulcis*, *P. mira*, *P. davidiana*, *P. kansuensis*, and *P. ferganensis*. **d** Percent of *P. davidiana*-specific contigs covered by reads from different *Prunus* species. **e** Regional collinearity between *P. davidiana* with *P. dulcis* or with *P. mira* genomes. The detailed location of these three regions were at Chr. 1: 4.79 Mb, Chr. 1: 5.08 Mb, and Chr. 2: 7.50 Mb in *P. davidiana* genome. Green and blue boxes indicate the positive and negative direction of genes. “P” and “A” letters showed the gene was presence and absence in *P. davidiana* genome

Next, we want to analyze the source of these *R* genes. Considering that the k-mer frequency of *P. davidiana* presents a characteristic of a high heterozygosity genome (Additional file 2: Fig. S1b), we speculated that there was a hybridization event in the evolutionary history of *P. davidiana*. To further verify this inference, one hundred and twenty-six accessions of wild peach species (Additional file 1: Table S18; accessions 101-126) were resequenced, and the obtained sequences (Additional file 1: Table S19) were aligned to the *P. mira* genome to obtain a total of 839,431 high-quality SNPs. Using these SNPs, we constructed a phylogenetic tree and found that cherries, plums, and apricots had a close relationship with *P. persica* and its wild related species. And during the latter category, *P. dulcis*, *P. tangutica*, *P. davidiana*, and *P. mira* formed a small subgroup (Fig. 2a). Principal component analysis (PCA) also demonstrated that *P. dulcis* or *P. tangutica* is located between *P. mira* and *P. davidiana* in the diagram of PC2-PC3 (Fig. 2b). We then phenotyped the stone streak of different species and found that abundant dot streaks were present in the *P. davidiana* but absent in the more recent species, *P. kansuensis*. However, the dot streaks could trace back to the ancient species of this family, such as *P. dulcis* (Fig. 2c). According to the above evidence, we proposed that *P. davidiana* showed intermediate genomic characteristics between *P. mira* and *P. dulcis* and might originate from the cross between these two species. A previous study [4] reported that *P. tangutica* has a closer relationship with *P. davidiana*. However, since no reference genome is available for *P. tangutica*, a direct comparison of genes from *P. davidiana* and *P. tangutica* is not feasible. Therefore, we first aligned the sequences of *P. davidiana* to its putative ancestor, *P. mira*, and assembled the unmapped sequences to obtain a partial reference genome. Genome resequencing data of different species were then aligned to this partial genome. We found that the highest mapping rate was an accession belonging to *P. dulcis*, and next was *P. tangutica* (Fig. 2d), which again proved that the introgression in *P. davidiana* came from *P. dulcis* or *P. tangutica*. In addition, all the genes in *P. davidiana* were aligned with those from the putative ancestors who had an assembled genome, including *P. mira* and *P. dulcis* (Additional file 2: Fig. S14). The genes were then assigned as originating from a specific species if the highest score was observed from the alignments of orthologous genes between *P. davidiana* and the other species. The results showed that although about 50.74% of the genes (13,719) were specific to *P. mira*, 26.87% (7,266) were thought to be derived from *P. dulcis*, and no more than 8% from other species.

To further study the evolutionary events leading to the genome structure of *P. davidiana*, we investigated the chromosome-to-chromosome relationships based on 16 (*P. davidiana* versus *P. mira*, including 21,186 genes) and 28 (*P. davidiana* versus *P. dulcis*, including 18,480 genes) identified syntenic blocks. The mosaic syntenic patterns again demonstrated that *P. davidiana* might have arisen during the evolutionary process of *P. mira* but with a cross of other species (Additional file 2: Fig. S15). Regional collinearity between *P. davidiana* with *P. dulcis* or *P. mira* indicated that some regions contained PAVs in *P. davidiana* genome showed a high structural similarity with them in *P. dulcis* (Fig. 2e). Although *P. dulcis* is mainly distributed in Georgia, Azerbaijan, Turkey, Syria, and Xinjiang province (China), it can also be found in Sichuan province of China where some *P. mira* grow in this region at the same time, indicating the hybridization event is highly possible (Additional file 2: Fig. S16).

We finally analyzed the origin of *R* genes in *P. davidiana* (Additional file 2: Fig. S12) and found 57.25% of all *R* genes were categorized to originate from *P. mira*, followed by *P. dulcis* (23.05%). Therefore, we believed that the cross between *P. mira* and *P. dulcis* or *P. tangutica* enhanced the adaptation of *P. davidiana* when it was spread to new environments.

### An indel in the promoter of an NBS-LRR gene regulates the nematode resistance in *P. kansuensis*

**Fig. 3.**
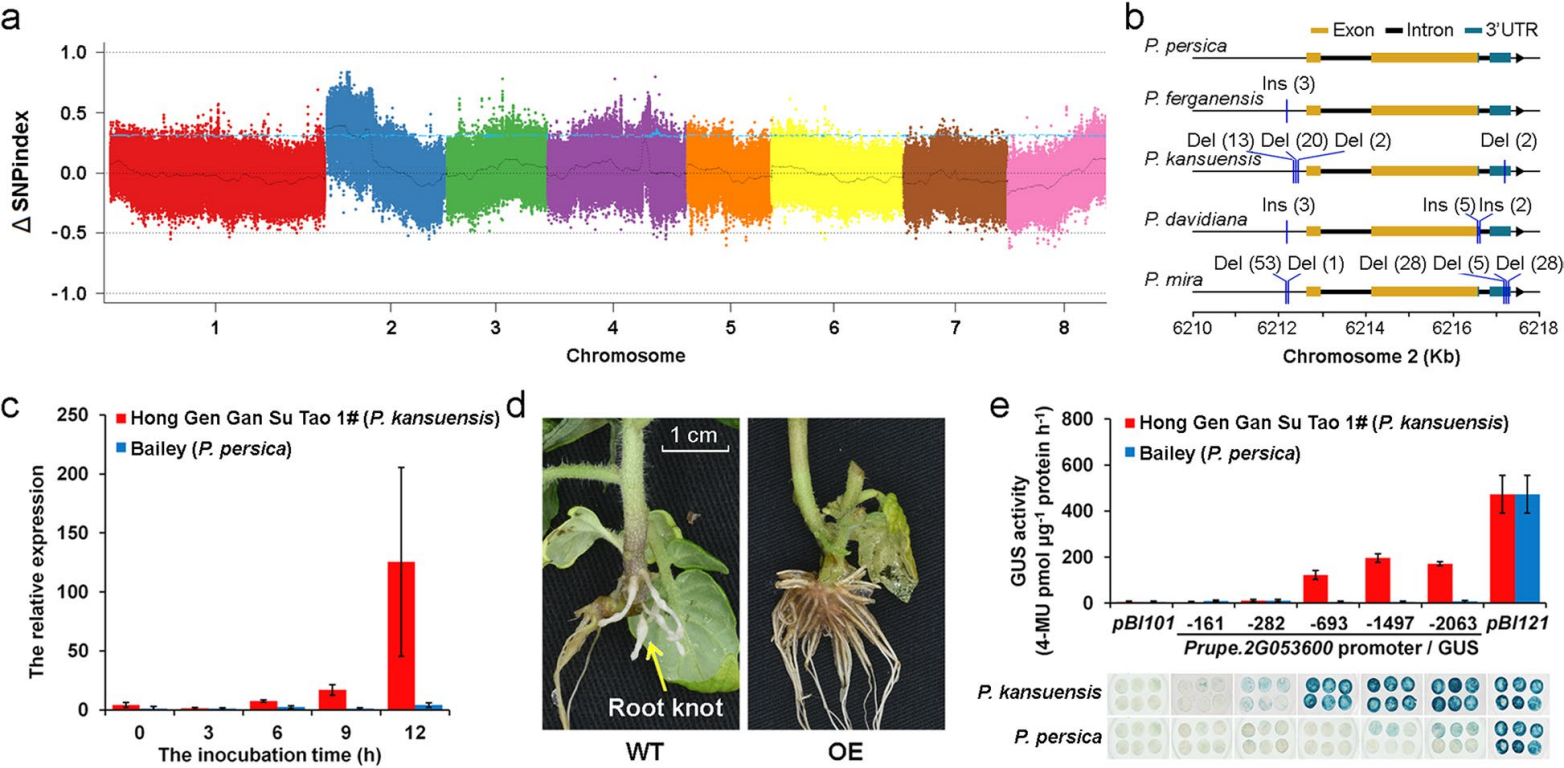
Identification of nematode resistance genes in *P. kansuensis*. **a** Bulked segregation analysis to locate the nematode resistance gene using a backcross population. **b** The Indels in the promoter and mRNA regions of one *NBS-LRR* genes on Chr. 2. “Ins” and “Del” indicate an insertion and deletion, respectively. And the number after the variation type indicates the length (bp) of Indels. **c** Expression of *Prupe.2G053600* in two accessions (“Hong Gen Gan Su Tao 1#” and “Bailey”) inoculated with nematode. **e** Functional validation of *Prupe.2G053600* through analysis of transgenic tomato plants expressing *Prupe.2G053600* under nematode treatment. **e** Promoter activity assay. Promoters with different lengths were fused to the *GUS* gene in plasmid *pBI101*. GUS was dyed, and its activity was measured using protein extracts of tobacco. *pBI101* and *pBI121* indicate negative control and positive control, respectively

*P. kansuensis* shows high resistance to root-knot nematodes [23]. To discover candidate causal sequence variations involved in the resistance, we constructed a backcross (BC_1_) population from the cross between “Hong Gen Gan Su Tao 1#” (*P. kansuensis*) and a cultivated peach “Bailey” (*P. persica*). “Hong Gen Gan Su Tao 1#” harbored high resistance to root-knot nematodes (*Meloidogyne incognita*), whereas other accessions including *P. mira*, *P. davidiana*, and *P. ferganensis* all showed low resistance to *M. incognita* [23]. Using this BC_1_ population, a nematode resistance locus was mapped at the top region of chromosome 2 (5.0-7.0 Mb) based on bulk segregation analysis (BSA) (Fig. 3a). The most significant locus was located at 6,671,644 bp of chromosome 2. In other species, the *R* genes to nematodes generally encode proteins containing the NBS-LRR domain [24]. Moreover, previous reports showed that the expression level of *R* gene which conferring root-knot nematode resistance was significantly higher in the roots of resistant plants than that of susceptible one and its value drastically increased when the plants were inoculated with nematodes [25, 26]. Therefore, transcriptome data [27] of two cultivars (resistant and susceptible to nematode infection, respectively) were analyzed again. We found that three of 19 expressed *NBS-LRR* genes (*Prupe.2G046000*, *Prupe.2G053600*, and *Prupe.2G055500*) have higher expression levels in “Hong Gen Gan Su Tao 1#” than those of “Bailey” during most inoculation periods (Fig. S17). However, only *Prupe.2G053600* was induced by root-knot nematodes in 6 or 12 h of resistant accession than that of 0 h (Additional file 2: Fig. S17; Fig. 3c). In the regions, we further focused on the genome variations mainly including small indels and SVs which occurred in the 24 *R* genes and their promoter. We found that there were 92 variations which only present in *P. kansuensis* but not in other species (Additional file 1: Table S20). Among them, *Prupe.2G053600*, which comprised a large deletion (Chr. 2: 6,212,387..6212,422 bp) in the promoter was further analyzed (Fig. 3b).

Then, the coding sequence of this gene was inserted into the plant expression vectors and transformed into tomato (cv. Micro-Tom). The transgenic lines were validated by genomic polymerase chain reaction (PCR) and qRT-PCR. We found the most transgenic lines showed remarkable nematode resistance with less root knots compared to control plants (Fig. 3d; Additional file 2: Fig. S18).

We validated this promoter deletion (35 bp) and found that it co-segregated with resistant phenotype of the seedlings in the BC_1_ population. To identify the active region of the promoter in *Prupe.2G053600*, we amplified 161, 282, 693, 1497, and 2063 bp of the 5′ flanking region of the gene in “Hong Gen Gan Su Tao 1#’ and linked the amplified products with the β-glucuronidase (GUS) coding sequence to transiently transform into *Nicotiana tabacum*. Leaves from the transgenic lines were analyzed for GUS activity by histochemical GUS staining and GUS quantitative enzyme activity determination. The lines carrying the various *Prupe.2G053600* promoters displayed remarkable but lesser GUS activity in comparison with the CaMV35S transformed one (pBI121 vector). An increase in GUS expression was observed with promoters longer than 693 bp, indicating the deletion (310 bp ahead of start codon) could drive the expression of *Prupe.2G053600* (Fig. 3e). We also constructed a mutation by recovery of 35 bp deletion to the promoter of “Hong Gen Gan Su Tao 1#” peach and linked it with *GUS* gene. The result found that the GUS activity of this mutation was significantly inhibited compared with control (Additional file 2: Fig. S19). In addition, we analyzed the difference of cis-elements in the promoter region of the resistant accession “Hong Gen Gan Su Tao 1#” and the susceptible one “Bailey.” The result showed that although the former accession was less than the latter by four TATA-box Elements, three more CAAAT-box elements in the former than that of the latter may be the main reason for its higher activity in regulating the expression of the target gene (Additional file 1: Table S21).

### Combined RNA-seq and selection sweep analysis to dissect the high-altitude adaptability in *P. mira*

*P. mira* is an attractive model for studying high-altitude adaptability of perennial plants because it originated in the QTP in China. The region has an average elevation of ∼4000 m above the sea level, and the oxygen concentration is ∼40% lower and UV radiation is ∼30% stronger than those at the sea level [28]. Up to date, knowledge on the mechanism of high-altitude adaptability has been reported in pig [29], yak [30], human [28, 31], snake [32], hulless barley [33], and the herbaceous plant *Crucihimalaya himalaica* [34]. However, little is known in perennial crops about the genetic basis of response to harsh conditions, such as low temperature and high UV radiation in high-altitude environments.

**Fig. 4.**
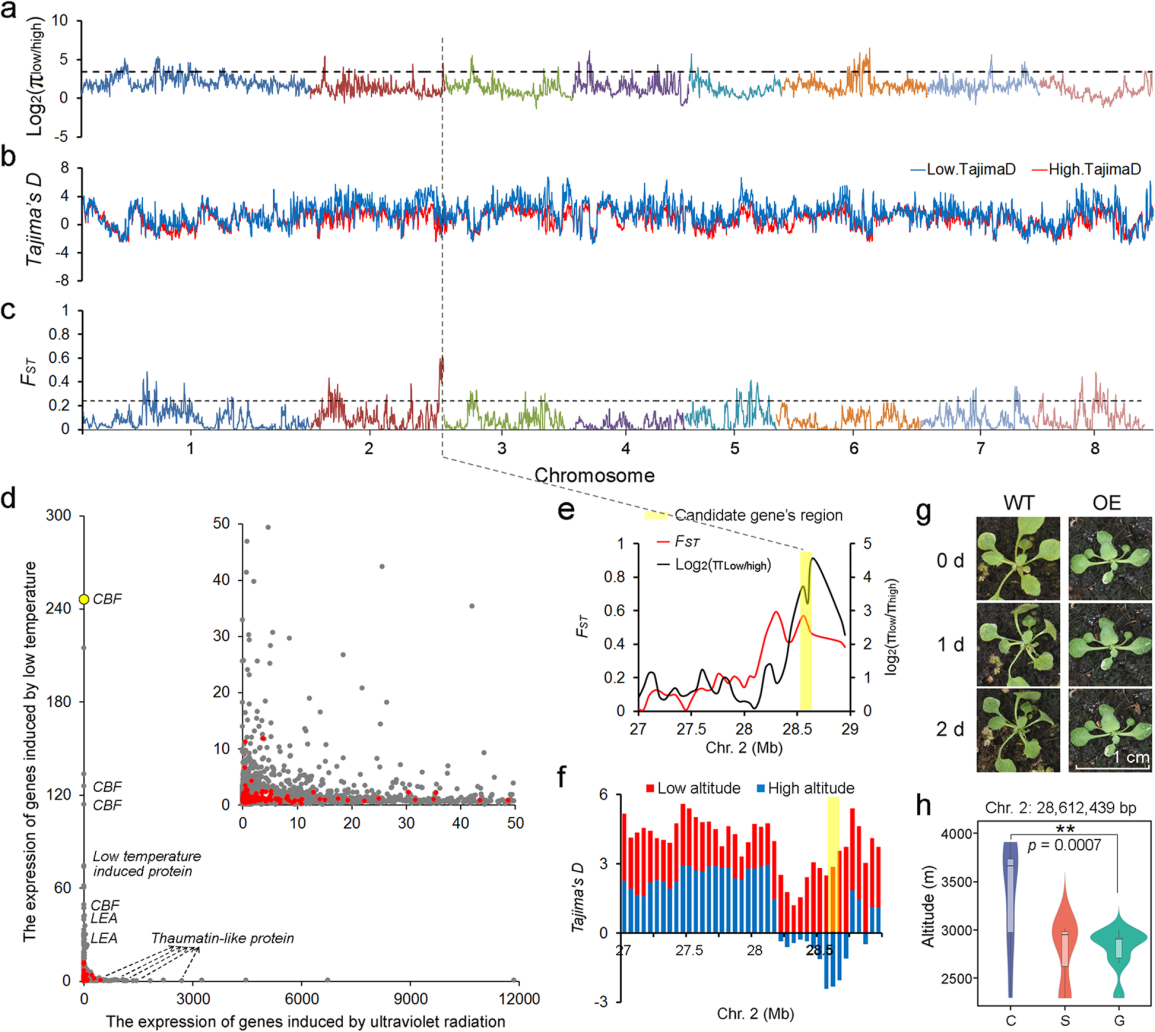
Selective regions associated with high-altitude adaptation in *P. mira*. **a**–**c** Domestication signals in accessions originating in high-altitude region compared to those in low-altitude. The signals were defined by the top 5% of π_ratio_ (**a**), Tajima’s *D* (**b**), and *F*_ST_ values (**c**). **d** Distribution of expression of genes induced by low temperature and UV of *P. mira*. Grey dots indicate the background genes and red dots indicate selective genes associated with high-altitude adaptation. **e** Detailed π ratio and *F*_*ST*_ values in the genome region of the candidate gene, *Pmi02g3025* (pointed by the dashed line), which was substantially induced by low temperature. **f** Detailed Tajima’s *D* in the genome region of the candidate gene *Pmi02g3025*. **g** *A. thaliana* plants expressing *Pmi02g3025* gene (OE) and the control (WT) treated with low temperature. **h** Genotypes (K indicates G/T) of a variation (Chr. 2: 28,612,439 bp) located at the promoter of *Pmi02g3025* in accessions from different altitude regions

Population genomic analyses were performed to analyze the high-altitude adaptability of *P. mira*. A total of 49 accessions (Additional file 1: Table S18; Accessions 127-175) belonging to the species with an altitude ranging from 2293 to 4390 m were resequenced to an average depth of 24.96×, and the sequencing reads were aligned to the *P. mira* genome to identify a total of 1,187,611 SNPs. Based on the altitude of these samples (Additional file 2: Fig. S20), the phylogenetic and structure analyses using the identified SNPs, 12 accessions thought to have not corresponded to their altitude categories or in an admixture subgroup between high and low altitude subgroups were excluded in the following analysis. Finally, ten accessions were classified into a high-altitude subgroup and 27 into the low-altitude subgroup (Additional file 2: Fig. S21). We calculated and compared the nucleotide diversity (π; Fig. 4a), Tajima’s *D* (Fig. 4b), and fixation index (*F*_*ST*_, Fig. 4c) values using SNPs across the genome of high- and low-altitude groups, resulting in the identification of selective sweeps of a total of 1.9 Mb and containing 231 genes (Additional file 1: Table S22). These genes were mostly involved in resistance to a series of stresses, such as cold, UV light, and DNA damage (Additional file 1: Table S23; Additional file 2: Fig. S22).

Furthermore, using the young seedlings of *P. mira* treated under low temperature (0 °C) and UV-light (~ 100 μW/cm^2^) for 10 h, we found that most genes presented stronger induction by UV than by low temperature based on the RNA-Seq data (Fig. 4d). Gene expression analysis showed that genes encoding C-repeat binding transcription factor (CBF) and late embryogenesis abundant protein (LEA) are strongly expressed after being induced by low temperature, while the enrichment of thaumatin-like protein plays a key role in the UV response in *P. mira*. One *CBF* gene, *Pmi02g3025*, showed more than 240-fold induction of expression by cold and about 6-fold induction by UV-light. According to the *F*_*ST*_ and Tajima’s *D* values, this gene was indeed under selection by altitude (Fig. 4e, f). In addition, we heterologously expressed the *Pmi02g3025* gene in Arabidopsis and treated the T_2_ transgenic plants under 0 °C for 48 h. The transgenic Arabidopsis seedlings showed increased resistance to low temperature compared to the wild type (Fig. 4g). We found that the gene expression of *Pmi02g3025* at 2 h after cold induction were higher than 0 h in the transgenic plants (Additional file 2: Fig. S23). And the change of proline contents in wild-type and transgenic plants after cold induction also proved transgenic ones have a higher resistance to low temperature (Additional file 2: Fig. S24).

Based on resequencing data, we found seven SNPs and an indel in the promoter regions having a strong association with the phenotype, such as a SNP (Fig. 4 h) and a 19 bp deletion (Additional file 2: Fig. S25) located at 1222 bp and 212 bp upstream of the start codon, respectively. The minor allele frequency of the former was 0.72 and 0 in low and high altitude groups, respectively. And the resequencing results showed that all high-altitude varieties had a 19 bp deletion in promoter of *Pmi02g3025*. All these results indicated that an obvious divergence existed between high and low altitude groups. Subsequent experiments also proved the relationship between the deletion and promoter activity.

## Discussion

In peach, a high-quality reference genome of *P. persica* was released and has since widely used as a valuable resource for effectively mining candidate genes for important traits [14]. However, this genome sequence alone is not adequate to uncover wild-specific sequences which might have been lost during domestication or artificial selection [35]. In this study, we de novo assembled the genomes of four high-quality wild relatives of *P. persica*. We analyzed the divergence time of peach and its relatives and found that the divergence time of Prunus genus from others was 56.6 Mya, later than that in other studies, 66.2 Mya [16]. However, the divergence time between the wild relative of peach and almond was 13.3 Mya, which was significantly earlier than previous report, 4.99 Mya [4]. We believe that the more number of species analyzed in our study and the differential software used in previous two studies and our (BEAST vs PAML software) was the main reasons for the differences between different reports.

Furthermore, using this large-scale comprehensive dataset, millions of genomic variations including SNPs and SVs were identified. In addition, hundreds of specific gene families in each of the wild peach species were also identified. The above gene sets represent a useful source for in-depth functional genomic studies including the identification of a nematode resistance gene from *P. kansuensis*. We identified the genotype of the 35 bp deletion located in the promoter of *Prupe. 2G053600* in other accessions and found that it has no correlation with resistance (Additional file 1: Table S24). In addition, the putative interspecific hybridization of *P. davidiana* identified in this study expands our understanding of the evolutionary path of the species and the molecular mechanisms underlying multiple resistance traits. Moreover, based on expanded gene families and comparative genomic analysis using different accessions of *P. mira* originating from low- and high-altitude regions, we found that the *CBF* genes play an important role in high-altitude adaptation of *P. mira*. However, analyzing the distribution of the 19 bp deletion in varieties with different low temperature resistance indicated that the variation was specific in *P. mira* which lived in high altitude region (Additional file 1: Table S25).

In addition, we found that gene families involved in vitamin B6 metabolism were significantly expanded in *P. mira* and *P. ferganensis*. Further analysis indicated that among the above expanded gene families, a total of 148 genes encoding pyridoxal 5′-phosphate synthase were identified in *P. mira* and the corresponding gene numbers decreased to 6, 19, and 92 in *P. davidiana*, *P. kansuensis*, and *P. ferganensis*, respectively. Enzyme encoded by this gene family catalyzes the hydrolysis of glutamine to glutamate and ammonia as part of the biosynthesis of pyridoxal 5'-phosphate [36]. It was reported that pyridoxal 5'-phosphate was an essential compound to improve salt-stress tolerance in Arabidopsis [37], wheat [38] and rice [39]. The component also responded to several stresses such as chilling, UV radiation, and drought in tobacco [40]. However, there were few reports on the involvement of the vitamin B6 metabolic pathway in altitude adaptation in plants. Interestingly, the researcher compared gene families in the genome of *Triplophysa bleekeri*, an endemic fish inhabiting high-altitude regions, to those fish species living in non-QTP regions and found that the expanded gene families in former species were primarily enriched in categories of vitamin B6 metabolism [41]. Furthermore, after sequencing the 16S rRNA genes of oral [42] or skin [43] microbiomes which sampled from Tibetans at different altitude in Tibet, the vitamin B6 metabolic pathway was all found to be enriched in the higher altitude group. Therefore, the expansion of gene involved in vitamin B6 metabolism in *P. mira* might explain its high-altitude adaptability.

In recent years, we and other groups have made important progress in peach evolution, divergence, and excellent genes identification. However, the main ideas of these studies were different from that of this study. For example, by performing large-scale resequencing of 10 wild and 74 cultivated peach varieties, we show that *P. mira* is the most primitive wild species of peach, and *P. ferganensis* can be classified as a subgroup of *P. persica* [3]. Obviously, gene introgression analysis was not performed in the above study. However, in the study, we constructed the phylogenetic trees of different species and speculated that the specific genes in *P. davidiana* may be derived from the hybridization of *P. mira* and almond (Fig. 2). To verify this speculation, *P. kansuensis* and *P. davidiana* genomes were firstly aligned to *P. dulcis*. The results showed that the mapping rate of the latter (65.15%) was slight higher than that of the former (63.73%). At the same time, these two genomes were also aligned with *P. mira* and found that the rate was 68.52% in the latter, which was also slight higher than the former (66.91%), confirming that *P. kansuensis* was more evolutional than *P. davidiana* during peach evolution. Different from this result, Yu et al. [4] found that an ancient introgression that existed between *P. mira* and the common ancestor of *P. kansuensis* and *P. persica* might lead to the development of fruit edibility in peach. Genome assembly and identification and functional verification of several excellent genes are not involved in the above studies. Here, we first completed the assembly of four wild species of *P. persica* and identified the nematode-resistant genes of *P. kansuensis* and the altitude adaptability-related genes of *P. mira*. However, there are still significant differences between the discovery of important genes in the study and that reported before [8] which mainly focused on fruit quality traits.

Moreover, when analyzing the genetic variations in key genes controlling various adaptive traits of different peach species, we found that the source of variations was diverse, including gene expansion, hybridization, and mutation. In previous studies, genome expansion and contraction of repetitive elements within the genome was reckoned to fuel evolutionary diversity in many plant species [44]. And adaptive introgression or sex might represent more efficient management option to conserve evolutionary potential in a changing environment [45, 46]. In view of the different origin of peach species, we speculated that the different sources of the variations were related to the intensity of the environmental change of the species. But, more evidences are needed to prove the precise mechanism underlying plant adaptation to changing environment during evolution.

## Conclusions

In summary, the study reported four high-quality genome assemblies and found different wild species contained a large number of specific genetic variations, including PAV and indels. To demonstrate their utility for basic genetics research and plant breeding applications, the role of some genetic variations in the resistance formation in wild peach species were dissected. We believe these genomic resources will increase our understanding of the evolution of fruit crops and be available to accelerating crop improvement in future.

## Methods

### Plant materials

In this study, different samples were used for DNA sequencing. First, genomes of four wild accessions (2010-138, Zhou Xing Shan Tao 1#, Hong Gen Gan Su Tao 1#, and Ka Shi 1#) were sequenced and de novo assembled. Second, 175 peach accessions were selected for genome resequencing. The above accessions were conserved in the National Germplasm Resource Repository of Peach at Zhengzhou Fruit Research Institute, Chinese Academy of Agricultural Sciences, China. Among them, most accessions belonging to *P. mira* were sampled from Tibet with different altitudes. Third, one BC_1_ population was constructed between “Hong Gen Gan Su Tao 1#” (*P. kansuensis*) and “Bailey” (*P. persica*) to identify QTLs linked to nematode resistance. Resistance to nematode in this BC_1_ population was evaluated previously by our group [3]. Genomic DNA was extracted from young leaves using the Plant Genomic DNA kit (Tiangen, Beijing, China).

Moreover, different samples were used for RNA sequencing (RNA-Seq). First, young leaves, mature fruits, seeds, phloem, and roots (obtained through asexual reproduction) of *P. persica* (Shang Hai Shui Mi), *P. ferganensis* (Kashi 1#), *P. kansuensis* (Hong Gen Gan Su Tao 1#), *P. davidiana* (Hong Hua Shan Tao), and *P. mira* (2010-138) were collected. Second, roots of “Hong Gen Gan Su Tao 1#” and “Bailey” infected with *Meloidogyne incognita* for 3, 6, 9, 12 h were collected for RNA-Seq analysis which was reported before [27].

### Genome sequencing of wild peach species

The genomes of four wild species of peach were sequenced using different platforms including PacBio Sequel and Illumina according to the manufacturers’ protocols. Library construction and sequencing were performed at Novogene Bioinformatics Technology Co., Ltd (Tianjin, China). For short-read sequencing, two short-insert libraries (230 bp and 500 bp) and 4 large-insert libraries (2 kb, 5 kb, 10 kb, and 20 kb) were constructed for *P. mira* and *P. davidiana*, while two short-insert libraries and 2 large-insert libraries (2 kb and 5 kb) were constructed for *P. kansuensis* and *P. ferganensis*. These libraries were sequenced on an Illumina HiSeq X Ten platform (Illumina, San Diego, CA).

For single molecule real-time (SMRT) sequencing, a 15-kb library was constructed and sequenced on the PacBio Sequel II platform (Pacific Biosciences, Menlo Park, CA). On the basis of subreads, the ccs software (https://github.com/PacificBiosciences/ccs) was used to generate HiFi reads. The parameter was set as below: min-passes = 3, min-rq = 0.99.

For chromosome conformation capture (Hi-C) sequencing, leaves fixed in 1% (vol/vol) formaldehyde were used for library construction. Cell lysis, chromatin digestion, proximity-ligation treatments, DNA recovery, and subsequent DNA manipulations were performed as previously described [47]. DpnII was used as the restriction enzyme in chromatin digestion. The Hi-C library was sequenced on the Illumina HiSeq X Ten platform (Illumina, San Diego, CA) to generate 150 bp paired-end reads.

### RNA-Seq data generation

To assist protein-coding gene predictions, we performed RNA-Seq using five different tissues for each species of 15 years old, and for each sample, three independent biological replicates were generated. Total RNA was extracted with the RNA Extraction Kit (Aidlab, Beijing, China), following the manufacturer’s protocol. RNA-Seq libraries were prepared with the Illumina standard mRNA-seq library preparation kit and sequenced on a HiSeq 2500 system (Illumina, San Diego, CA) with paired-end mode.

### Genome assembly of wild peach species

The genome sizes of the four wild peach species were estimated by K-mer analysis. The occurrences of K-mer with a peak depth were counted using Illumina paired-end reads, and genome sizes were calculated according to the formula: total number of K-mers/depth at the K-mer peak, using JELLYFISH 2.1.3 software [48] with K set to 17. Firstly, all HiFi reads which obtained from the above procedure were *de novo* assembled using Hifiasm (v0.13-r308) [49]. Then, Hi-C data were used to correct superscaffolds and cluster the scaffolds into pseudochromosomes through ALLHiC [50] pipeline.

To evaluate the quality of the genome assemblies, we performed BUSCO v4.0.0 [15] analysis on the four assembled genomes with the 1,614 conserved plant single-copy orthologs. Moreover, RNA-Seq data with different tissues were used to validate the results of gene prediction (Additional file 1: Table S5).

### Repetitive element identification

A combined strategy based on homology alignment and de novo search was used to identify repeat elements in the four wild peach genomes. For de novo prediction of TEs, we used RepeatModeler (http://www.repeatmasker.org/RepeatModeler.html), RepeatScout (http://www.repeatmasker.org/), Piler [51], and LTR-Finder [52] with default parameters. For alignment of homologous sequences to identify repeats in the assembled genomes, we used RepeatProteinMask and RepeatMasker (http://www.repeatmasker.org) with the repbase library [53]. TEs overlapping with the same type of repeats were integrated, while those with low scores were removed if they overlapped more than 80 percent of their lengths and belonged to different types.

### Gene prediction and functional annotation

Gene prediction was performed using a combination of homology, ab initio and transcriptome based approaches. For homology-based prediction, protein sequences from *P. persica*, *Pyrus bretschneideri*, *P. mume*, *Malus domestica*, and *Fragaria vesca* (Genome Database for Rosaceae; https://www.rosaceae.org) and *Vitis vinifera* (http://www.genoscope.cns.fr/externe/GenomeBrowser/Vitis/) and *Arabidopsis thaliana* (https://www.arabidopsis.org) were downloaded and aligned to the peach assemblies. Augustus [54], GlimmerHMM [55], and SNAP [56] were used for ab initio predictions. For transcriptome-based prediction, RNA-Seq data derived from root, phloem, leaf, flower, and fruit were mapped to the assemblies using HISAT2 software [57] and assembled into the transcripts using Cufflinks (version 2.1.1) with a reference-guided approach [58]. Moreover, RNA-Seq data were also de novo assembled using Trinity v2.0 [59] and open reading frames in the assembled transcripts were predicted using PASA [60]. Finally, gene models generated from all three approaches were integrated using EvidenceModeler [60] to generate the final consensus gene models.

The predicted genes were functionally annotated by comparing their protein sequences against the NCBI non-redundant (nr), Swiss-Prot (http://www.uniprot.org/), TrEMBL (http://www.uniprot.org/), Kyoto Encyclopedia of Genes and Genomes (KEGG, http://www.genome.jp/kegg/), InterPro, and Gene ontology (GO) databases.

tRNAscan-SE [61] was used with default parameters to identify tRNA sequences in the genome assemblies. rRNAs in the genomes were identified by aligning the reference rRNA sequence of relative species to the assemblies using BLAST with *E*-values < 1e^−10^ and nucleotide sequence identities > 95%. Finally, the INFERNAL v1.1 (http://infernal.janelia.org/) software was used to compare the genome assemblies with the Rfam database (http://rfam.xfam.org/) to predict miRNA and snRNA sequences.

### Genome alignment and collinearity analysis

Orthologous genes within the *P. mira* and *P. persica* genomes were identified using BLASTP (*E* value <1e^−5^), and MCScanX [62] was used to identify syntenic blocks between the two genomes. The collinearity of the two genomes was then plotted according to the identified syntenic blocks.

Four wild peach genomes were aligned to the *P. persica* genome using LASTZ [63] with the parameters of “M=254K=4500 L=3000 Y=15000 --seed=match 12 --step=20 --identity=85.” In order to avoid the interference caused by repetitive sequences in alignments, RepeatMasker (http://www.repeatmasker.org) and RepBase library [53] were used to mask the repetitive sequences in genomes of *P. persica* and four wild species. The raw alignments were combined into larger blocks using the ChainNet algorithm implemented in LASTZ [63].

### Variation identification

We identified SNPs and small indels (< 50 bp) between the four wild and reference peach genomes using SAMtools (http://samtools.sourceforge.net/) and LAST (https://gitlab.com/mcfrith/last) with parameters “-m20 -E0.05” and SNP and indel filtering criteria “minimum quality = 20, minimum depth = 5, maximum depth = 200.” SVs were identified from genome alignments by LAST with parameters “-m20 -E0.05.” The software has been used in many studies [64, 65] for long read or genome alignments. CNVs were identified using CNVnator-0.3.3 [66]. And observation by IGV software and PCR amplification (Additional file 2: Fig. S4; Additional file 1: Table S15 and 16) was used to confirm the accuracy of SVs.

### BSA to locate nematode resistance gene

Using a previously constructed BC_1_ population [24], we extracted genomic DNA from fresh leaves of its parents and two bulked populations which mixed by 20 resistance seedlings and 20 susceptible ones. The samples were used for resequencing on an Illumina HiSeq 2500 platform (Illumina, San Diego, CA) to generate 125 bp paired-end reads. Then BWA software [67] was used to align the clean reads of each sample against the reference genome of *Prunus persica* v2.0.a1 [14].

Variants calling were performed for all samples by using GATK3.3 software [68] to identify a total of 2,442,036 SNPs on all 8 chromosomes. The homozygous SNPs between two parents were extracted from the vcf files. The reads depth information for homozygous SNPs above in the offspring pools was gained to calculate the SNP index [69]. The loci whose SNP index was less than 0.3 or greater than 0.7 in both the pools were filtered. The difference of the SNP index of the two pools was calculated as the Δ(SNP index). Finally, we made a graph of Δ(SNP index) of the whole-genome by the sliding window method.

### Promoter activity measurement

A total of five primers upstream and one downstream of the start codon of *Prupe.2G053600* were synthesized and used to amplify a series of 5 indel regions in the *Prupe.2G053600* promoter using PCR amplification. The amplified PCR products were ligated into pGEM-T easy vector and cloned into pBI101 binary vector after being digested by XbaI and BamHI. Furthermore, each of the 5 amplified products was transformed into *A. tunefaciens* (GV1301) cells and collected and resuspended in infiltration buffer and then transformed into 6-week-old tobacco leaves using sterilized syringes. The transiently transformed tobacco plants were grown in a growth chamber for 48 h and the infection sites were cut to measure GUS activity as previously described in Jefferson et al. [70]. The pBI121 vector was used as a positive control.

### Transgenic analysis

The full-length open reading frame of the *Prupe.2G053600* gene was amplified through PCR using cDNA synthesized from RNA that was isolated from root of the “Hong Gen Gan Su Tao 1#” (*P. kansuensis*). The amplified product was cloned into the pEASY vector driven by the cauliflower mosaic virus (CaMV) 35 S promoter. The resulting vector was transformed into *Solanum lycopersicum* cv. Micro-Tom by *A. tumefaciens* C58. The T_0_ plants were generated and inoculated with *M. incognita* to observe resistance and measure gene expression.

Similarly, one candidate gene, *Pmi02g3025*, was cloned from the leaf of “2210-198” (*P. mira*) and ligated to the vector and transformed into *A. thaliana* “Columbia.” When the transformed plants were grown to about 5 leaves, low temperature (0 °C) treatment was applied and samples were collected at 0, 24, and 48 h post treatment. Phenotype was observed and the drooping leaves were used to indicate that the accession was susceptible to low temperature. The proline content of leaves in Arabidopsis was measured according to previous reference as previously described [71].

### Comparative analysis

Protein sequences from 15 plant species including *P. persica* (phytozomev10), *P. ferganensis*, *P. kansuensis*, *P. davidiana*, *P. mira*, *Prunus dulcis* (https://www.rosaceae.org/species/prunus/prunus_dulsis/lauranne/genome_v1.0), *P. armeniaca* (https://www.rosaceae.org/species/prunus_armeniaca/genome_v1.0), *Prunus mume* (http://prunusmumegenome.bjfu.edu.cn/index.jsp), *P. salicina* (NCBI accession: PRJNA574159), *P. avium* (NCBI accession: PRJNA395588), *M. domestica* (NCBI accession: PRJNA534520), *P. bretschneideri* (NCBI accession: PRJNA259338), *Fragaria vesca* (phytozome v10), *A. thaliana* (phytozome v10), and *Vitis vinifera* (phytozome v10) were used to construct orthologous gene families. To remove redundancy caused by alternative splicing, we retained only the gene model at each gene locus that encoded the longest protein. To exclude putative fragmented genes, genes encoding protein sequences shorter than 50 amino acids were filtered out. All-against-all BLASTp was performed for these protein sequences with an E-value cut-off of 1e^−5^. OrthoMCL V1.4 [72] was then used to cluster genes into gene families with the parameter “-inflation 1.5.”

Protein sequences from 830 single-copy gene families were used for phylogenetic tree construction. MUSCLE [51] was used for multiple sequence alignment for protein sequences in each single-copy family with default parameters. The alignments from all single-copy families were then concatenated into a super alignment matrix, which was used for phylogenetic tree construction using the maximum likelihood (ML) method implemented in the RAxML software (http://cme.h-its.org/exelixis/web/software/raxml/index.html). Divergence times between the 15 species were estimated using MCMCTree in PAML software (http://abacus.gene.ucl.ac.uk/software/paml.html) with the options “correlated rates” and “JC69” model. A Markov Chain Monte Carlo analysis was run for 10,000 generations, using a burn-in of 10,000 iterations and sample-frequency of 2. Five calibration points were applied according to the TimeTree database (http://www.timetree.org): *A. thaliana* and *V. vinifera* (106.9-134.7 Mya), *A. thaliana* and the common ancestor of *F. vesca*, *M. domestica*, and other *Prunus* species (97.4-109.3 Mya), *F. vesca* and common ancestor of *M. domestica*, and other *Prunus* species (64.5-75.5 Mya), *M. domestica*, *P. bretschneideri*, and other *Prunus* species (51.7-61.8 Mya), *M. domestica* and *P. bretschneideri* (11.3-26.4 Mya).

To detect the whole genome duplication events, we first identified collinearity blocks using paralogous gene pairs with software MCScanX [62]. Using the sum of transversion of fourfold degenerate site divided by the sum of fourfold degenerate site, we then calculated 4dTv values of each block. In addition, *Ks* values of homologous gene pairs were also calculated using PAML [73] based on the sequence alignments by MUSCLE [51], to validate speciation times.

### Gene family expansions and contractions

Expansion and contractions of orthologous gene families were determined using CAFE [74], which uses a birth and death process to model gene gain and loss over a phylogeny. Significance of changes in gene family size in a phylogeny was tested by calculating the *p*-value on each branch using the Viterbi method with a randomly generated likelihood distribution. This method calculates exact *p*-values for transitions between the parent and child family sizes for all branches of the phylogenetic tree. Enrichment of GO terms and KEGG pathways in the expanded gene families of each of the four wild peach species were identified using the R package clusterProfiler [75].

### Identification of an interspecific hybridization of *P. davidiana*

Using SNPs identified from 126 peach accessions, we performed PCA to evaluate the evolution path using the software GCTA [76].

To trace the origin of genes in *P. davidiana*, each gene was aligned to other genomes to calculate the alignment score. Genes were classified as putatively originating from the specie which had the highest alignment scores. Collinearity among *P. dulcis*, *P. davidiana*, and *P. mira* was plotted based on the identified syntenic blocks.

### Resistance genes

Hidden Markov model search (HMMER; http://hmmer.janelia.org) was used to identify *R* genes in the four wild peach genomes according to the nucleotide-binding site (NBS) domain (PF00931), Toll and interleukin-1 receptor-like (TIR) model (PF01582), and several leucine-rich repeat (LRR) models (PF00560, PF07723, PF07725, PF12799, PF13306, PF13516, PF13504, PF13855, and PF08263) in the Pfam database (http://pfam.sanger.ac.uk). Coiled-coil (CC) motifs were detected using the COILS prediction program 2.2 (https://embnet.vital-it.ch/software/COILS_form.html) with a *p* score cut-off of 0.9.

### Identification of selective sweeps associated with high-altitude adaptation in *P. mira*

Raw genome reads of the 49 accessions of *P. mira* from Tibet, China with different altitudes were processed to remove adaptor, contaminated and low quality sequences, and the cleaned reads were mapped to the assembled *P. mira* genome using BWA v0.7.8 [67]. Based on the alignments, the potential PCR duplicates were removed using the SAMtools command “rmdup.” SNP calling at the population level was performed using SAMtools [77]. The identified SNPs supported by at least five mapped reads, mapping quality ≥ 20, and Phred-scaled genotype quality ≥ 5, and with less than 0.2 missing data were considered high-quality SNPs (1,394,483) and used for subsequent analyses.

A phylogenetic tree constructed using TreeBeST 1.9.2 [78] with 1000 bootstraps and structure analysis was performed for the 49 accessions of *P. mira* using the program frappe [79] with the number of assumed genetic clusters (K) ranging from two to three, and 10,000 iterations for each run.

To identify genome-wide selective sweeps associated with high-altitude adaptation, we scanned the genome in 50-kb sliding windows with a step size of 10 kb and calculated the reduction inπ based on the *P. mira* accessions originating in high-altitude to low-altitude regions (π_high_/π_low_). In addition, Tajima’s *D* and *F*_*ST*_ between the two groups were also calculated. Windows with the top 5% of the π ratios, Tajima’s *D* ratio, and *F*_ST_ values were considered as selective sweeps.

## Supplementary Information

**Additional file 1: Table S1**. Genome survey of four wild peach species (kmer = 17). **Table S2**. Summary of genome sequencing of four wild peach species. **Table S3**. Pseudochromosome lengths (bp) of the *P. mira*, *P. davidiana*, *P. kansuensis*, and *P. ferganensis* assembly. **Table S4**. BUSCO analysis of the genome assemblies of four wild peach species. **Table S5**. Mapping statistics of RNA-Seq reads to the corresponding genome assemblies of four wild peach species. **Table S6**. Statistic of repeat sequences in the assemblies of four wild peach species. **Table S7**. Prediction of protein-coding genes in the genomes of four wild peach species. **Table S8**. Statistics of predicted protein-coding genes in four wild peach species compared to other species. **Table S9**. Statistics of gene functional annotation in the four wild peach species. **Table S10**. Non-coding RNAs identified in genomes of four wild wild peach species. **Table S11**. SNPs identified between genomes of each of the four wild species and *P. persica*. **Table S12**. Small indels (<50 bp) identified between genomes of each of the four wild species and *P. persica*. **Table S13**. Structural variants (≥ 50 bp) between the four wild species and *P. persica*. **Table S14**. Statistics of copy number variations between the four wild species and *P. persica.* **Table S15**. A total of 20 SVs which aligned *P. mira* with *P. persica* genome*.* **Table S16**. The primers which designed to amplify the SVs between *P. mira* and *P. persica* genome*.* **Table S17**. Statistics of resistance genes in the four wild peach species. **Table S18**. List of 175 peach samples used in the study. **Table S19**. The summary statistics of genome resequencing. **Table S20**. Variations in the promoter and mRNA regions of *R* genes on Chr. 2 (5-7 Mb) those were specific to *P. kansuensis*. **Table S21**. The cis-elements in promoter region of *Prupe.2G053600* gene in nematode-resistant and susceptible accessions. **Table S22**. Genes selected between the two subgroups of *P. mira* which originated from high- and low-altitude regions. **Table S23**. Selected genes in the KEGG pathways associated with plateau adaptability. **Table S24**. The genotype of 35 bp deletion in the promoter of *Prupe. 2G053600* in varieties with different root-knot nematode resistance. **Table S25**. The genotype of 19 bp deletion in the promoter of *Pmi02g3025* in varieties with different cold resistance. **Table S26**. The primers used in the study.

**Additional file 2: Fig. S1**. Estimation of genome sizes of *P. mira* (a), *P. davidiana* (b), *P. kansuensis* (c), and *P. ferganensis* (d) based on K-mer analysis. **Fig. S2**. Genome size, annotated gene number, and the length of tandam repeat sequences, interpersed repeat sequences in *P. mira*, *P. davidiana*, *P. kansuensis*, and *P. ferganensis.* **Fig. S3**. Genome variations across the pseudo-chromosomes of four wild peach species compared to the reference (*Prunus persica*). The circles from the outer to the inner (A-P) represent copy number variation (CNV) density in *P. ferganensis* (A), *P. kansuensis* (B), *P. davidiana* (C), and *P. mira* (D), and structure variations (SVs) in *P. ferganensis* (E), *P. kansuensis* (F), *P. davidiana* (G), and *P. mira* (H), and indels in *P. ferganensis* (I), *P. kansuensis* (J), *P. davidiana* (K), and *P. mira* (L), as well as SNPs in *P. ferganensis* (M), *P. kansuensis* (N), *P. davidiana* (O), and *P. mira* (P) in each sliding window of 0.1 Mb. **Fig. S4**. The electrophoresis results of 20 primers which amplified in two varieties (2010-138 and Shen Zhou Li He Shui Mi) belonging to *P. mira* (*Pm*) and *P. persica* (*Pp*), respectively. **Fig. S5**. KEGG pathways enriched in genes comprising large-effect SNPs of between *P. ferganensis* and *P. persica*. **Fig. S6**. KEGG pathways enriched in genes comprising indels in four wild peach species compared to *P. persica*. **Fig. S7**. KEGG pathways enriched in genes comprising structure variations in *P. mira and P. kansuensiss* compared to *P. persica*. **Fig. S8**. KEGG pathways enriched in genes comprising copy number variations in *P. ferganensis* (a), *P. kansuensis* (b), *P. davidiana* (c), and *P. mira* (d) compared to *P. persica*. **Fig. S9**. Venn diagram of gene families identified from the five species of peach*.* **Fig. S10**. Statistics of single-copy orthologs, multiple-copy orthologs, and unique orthologs in 15 species. **Fig. S11**. KEGG pathways enriched in expanded and contracted gene families of the four wild peach species. **Fig. S12**. Whole-genome duplication and speciation events in peach as revealed by the distribution of 4DTv distance among paralogous and orthologs genes in different species. **Fig. S13**. Distribution of resistance (*R*) genes across the 8 chromosomes in five peach species of peach and their overlaps with disease resistance QTLs. **Fig. S14**. Percent of *P. davidiana*-specific contigs covered by reads from different *Prunus* species. **Fig. S15**. Collinearity among *P. davidiana*, *P. mira*, *P. dulcis*, *P. armeniaca*, and *P. avium* genomes. **Fig. S16**. Geographical distribution of *P. mira* (red circle), *P. davidiana* (yellow), and *P. dulcis* (orange) which originated in China. **Fig. S17** .Expression of 19 *R* genes in two accessions (‘Hong Gen Gan Su Tao 1#’ and ‘Bailey’) inoculated with nematode after 0, 6, 12, 36, 60, and 84 hours. **Fig. S18**. The evaluation of resistant to root-knot nematodes in transgenic tomato lines with *Prupe.2G053600* gene and its wild type. **Fig. S19.** The vector construction (a) and promoter activity assay (b) of *Prupe.2G253600* promoter which recovered 35 bp deletion in ‘Hong Gen Gan Su Tao 1#’ peach. **Fig. S20**. The altitude distribution of accessions used to identify altitude adaptability related genes for genome resequencing. **Fig. S21**. Phylogenetic tree of 32 accessions of *P. mira* (a) originating from regions with different altitudes (b) and the population structure (c) when K=2 and 3. **Fig. S22**. Two genome regions associated with high-altitude adaptation. **Fig. S23**. The gene expression of wild-type (WT) and three transgenic plants (OE1-OE3) in Arabidopsis which induced by 4 °C for different period. **Fig. S24**. The proline content of wild-type (WT) and transgenic plant (OE) in Arabidopsis which induced by 4 °C for 24 and 48 hours. **Fig. S25**. A deletion identified in the promoter of *Pmi02g3025* gene in varieties with different altitude.

## Data Availability

Raw resequencing data for 175 peach accessions generated in this study have been deposited into the NCBI database. And the accession numbers of Sequence Read Archive (SRA) were listed in Additional file 1: Table S19. In addition, the RNA-seq data of different tissues of four wild species and *P. mira* which were treated by UV or low temperature also have been deposited into the NCBI database with BioProject under accession PRJNA753201 [80] and PRJNA753549 [81]. The assemblies of four genomes have been uploaded to Genome Database for Rosaceae (https://www.rosaceae.org/Analysis/12080707;https://www.rosaceae.org/Analysis/11857924; https://www.rosaceae.org/Analysis/12080706; https://www.rosaceae.org/Analysis/12080705). The primers used in the study can be found in Additional file 1: Table S26.

## Abbreviations

QTP: Qinghai-Tibet Plateau
Mya: Million years ago
CNVs: Copy number variations
BSA: Bulked segregant analysis
UV: Ultraviolet rays
BUSCO: Benchmarking Universal Single-Copy Orthologs
LTR: Long terminal repeat
TEs: Transposable elements
SNPs: Single nucleotide polymorphisms
Indels: Small insertions and deletions
SVs: Structural variants
CNVs: Copy number variations
PAVs: Presence/absence variations
MCRA: Most recent common ancestor
4DTv: Fourfold synonymous third-codon transversion
*Ks*: Rate of synonymous mutation
PCA: Principal component analysis
BC_1_: Backcross
GUS: β-Glucuronidase
π: Nucleotide diversity
*F*_*ST*_: Fixation index
CBF: C-Repeat binding transcription factor
LEA: Late embryogenesis abundant protein
SMRT: Single molecule real-time
Hi-C: High-throughput chromosome conformation capture
GO: Gene ontology
KEGG: Kyoto Encyclopedia of Genes and Genomes
PCR: Polymerase chain reaction
RNA-Seq: RNA sequencing
CaMV: Cauliflower mosaic virus
ML: Maximum likelihood
NBS: Nucleotide-binding site
LRR: Leucine-rich repeat
TIR: Toll and interleukin-1 receptor-like
CC: Coiled-coil
SRA: Sequence read archive
