# Supplementary material for: Chromosome-level genome assemblies of four wild peach species provide insights into genome evolution and genetic basis of stress resistance
Source: BMC Biol. 2022 Jun 13;20:139. doi: 10.1186/s12915-022-01342-y (PMC9195245; doi:10.1186/s12915-022-01342-y)
Supplement: Supplementary file 1 — Additional file 1: Table S1. Genome survey of four wild peach species (kmer = 17). Table S2. Summary of genome sequencing of four wild peach species. Table S3. Pseudochromosome lengths (bp) of the P. mira, P. davidiana, P. kansuensis, and P. ferganensis assembly. Table S4. BUSCO analysis of the genome assemblies of four wild peach species. Table S5. Mapping statistics of RNA-Seq reads to the corresponding genome assemblies of four wild peach species. Table S6. Statistic of repeat sequences in the assemblies of four wild peach species. Table S7. Prediction of protein-coding genes in the genomes of four wild peach species. Table S8. Statistics of predicted protein-coding genes in four wild peach species compared to other species. Table S9. Statistics of gene functional annotation in the four wild peach species. Table S10. Non-coding RNAs identified in genomes of four wild wild peach species. Table S11. SNPs identified between genomes of each of the four wild species and P. persica. Table S12. Small indels (<50 bp) identified between genomes of each of the four wild species and P. persica. Table S13. Structural variants (≥ 50 bp) between the four wild species and P. persica. Table S14. Statistics of copy number variations between the four wild species and P. persica. Table S15. A total of 20 SVs which aligned P. mira with P. persica genome. Table S16. The primers which designed to amplify the SVs between P. mira and P. persica genome. Table S17. Statistics of resistance genes in the four wild peach species. Table S18. List of 175 peach samples used in the study. Table S19. The summary statistics of genome resequencing. Table S20. Variations in the promoter and mRNA regions of R genes on Chr. 2 (5-7 Mb) those were specific to P. kansuensis. Table S21. The cis-elements in promoter region of Prupe.2G053600 gene in nematode-resistant and susceptible accessions. Table S22. Genes selected between the two subgroups of P. mira which originated from high- and low-altitude regio [file 12915_2022_1342_MOESM1_ESM.docx]

**Supplementary Table 1** Genome survey of four wild peach species (kmer = 17).

| Species | K-mer number | K-mer depth | Genome size (Mb) | Heterozygous ratio (%) | Repeat (%) |
| --- | --- | --- | --- | --- | --- |
| ***P. mira*** | 12,594,677,936 | 51 | 242.94 | 0.76 | 47.51 |
| ***P. davidiana*** | 22,358,636,352 | 93 | 237.29 | 1.10 | 45.09 |
| ***P. kansuensis*** | 14,480,733,904 | 60 | 238.06 | 0.56 | 46.07 |
| ***P. ferganensis*** | 13,266,273,280 | 55 | 237.24 | 0.53 | 45.09 |

Note: all values were calculated according to the curve of depth distribution of K-mer number.

**Supplementary Table 2** Summary of genome sequencing of four wild peach species.

| Pair-end libraries | Insert size of libraries | *P. mira* | | *P. davidiana* | | *P. kansuensis* | | *P. ferganensis* | |
| --- | --- | --- | --- | --- | --- | --- | --- | --- | --- |
|  |  | Total data (Gb) | Coverage (×) | Total data (Gb) | Coverage (×) | Total data (Gb) | Coverage (×) | Total data (G) | Coverage (×) |
| Illumina | 230 bp | 14.99 | 61.70 | 14.30 | 60.26 | 13.8 | 52.08 | 12.9 | 54.38 |
|  | 500 bp | 15.43 | 63.51 | 15.63 | 65.87 | 5.6 | 21.13 | 5.2 | 21.92 |
|  | 2 Kb | 13.41 | 55.20 | 13.46 | 56.72 | 5.8 | 21.89 | 5.8 | 24.45 |
|  | 5 Kb | 6.57 | 27.04 | 9.71 | 40.92 | 6.8 | 25.66 | 6.2 | 26.13 |
|  | 10 Kb | 6.00 | 24.70 | 6.16 | 25.96 |  |  |  |  |
|  | 20 Kb | 2.57 | 10.58 | 3.31 | 13.95 |  |  |  |  |
| PacBio | 15 Kb | 14.75 | 60.71 | 31.13 | 131.18 | 17.21 | 64.95 | 26.54 | 111.88 |
| Hi-C | | 72.14 | 296.93 | 42.71 | 179.98 | 41.94 | 158.28 | 35.38 | 149.14 |
| Total | | 145.86 | 600.37 | 136.41 | 574.84 | 91.15 | 343.99 | 92.02 | 387.9 |

**Supplementary Table 3** Pseudochromosome lengths (bp) of the *P. mira*, *P. davidiana*, *P. kansuensis*, and *P. ferganensis* assembly.

| Chromosome | *P. mira* | *P. davidiana* | *P. kansuensis* | *P. ferganensis* |
| --- | --- | --- | --- | --- |
| Chr.1 | 48,690,834 | 47,219,106 | 49,206,398 | 49,129,830 |
| Chr.2 | 30,829,006 | 30,824,452 | 31,104,671 | 32,643,475 |
| Chr.3 | 27,933,902 | 28,549,251 | 28,885,292 | 28,247,033 |
| Chr.4 | 26,086,476 | 26,367,603 | 26,257,146 | 26,497,910 |
| Chr.5 | 19,785,507 | 20,435,266 | 20,106,291 | 19,782,008 |
| Chr.6 | 32,027,761 | 30,963,822 | 32,902,607 | 32,049,944 |
| Chr.7 | 24,355,622 | 24,106,639 | 24,889,959 | 24,017,386 |
| Chr.8 | 24,364,023 | 23,478,219 | 24,703,867 | 24,200,323 |
| Total | 234,073,131 | 231,944,358 | 238,056,231 | 236,567,909 |

**Supplementary Table 4** BUSCO analysis of the genome assemblies of four wild peach species.

| Species | BUSCO assessment result based on a total of 1440 searched BUSCO groups | | | |
| --- | --- | --- | --- | --- |
|  | **S (%)** | **D (%)** | **F (%)** | **M (%)** |
| *P. mira* | 97.1 | 2.0 | 0.3 | 0.6 |
| *P. davidiana* | 97.1 | 1.9 | 0.3 | 0.7 |
| *P. kansuensis* | 95.6 | 2.0 | 0.8 | 1.6 |
| *P. ferganensis* | 97.2 | 1.9 | 0.2 | 0.7 |

S: Complete and single-copy BUSCOs.

D: Complete and duplicated BUSCOs.

F: Fragmented BUSCOs.

M: Missing BUSCOs.

Note: More than 90% of embryophyta genes were detected in above table, indicating a good completeness of our assembly.

**Supplementary Table 5** Mapping statistics of RNA-Seq reads to the corresponding genome assemblies of four wild peach species.

| Tissue | *P. mira* (%) | *P. davidiana* (%) | *P. kansuensis* (%) | *P. ferganensis* (%) |
| --- | --- | --- | --- | --- |
| **Fruit** | 92.88 | 94.43 | 91.58 | 90.01 |
| **Flower** | 91.28 | 92.16 | 93.59 | 94.18 |
| **Phloem** | 87.19 | 94.01 | 89.51 | 87.02 |
| **Leaf** | 88.95 | 94.53 | 85.66 | 91.40 |
| **Seed** | 88.22 | 93.35 | 88.04 | 86.53 |

Note: the value indicates the percent of mapped reads to total sequenced reads in different accessions and tissues.

**Supplementary Table 6** Statistic of repeat sequences in the assemblies of four wild peach species.

| Category | | *P. mira* | | *P. davidiana* | | *P. kansuensis* | | *P. ferganensis* | |
| --- | --- | --- | --- | --- | --- | --- | --- | --- | --- |
|  |  | **Length (bp)** | **% in genome** | **Length (bp)** | **% in genome** | **Length (bp)** | **% in genome** | **Length (bp)** | **% in genome** |
| Total repeat | | 122,714,191 | 48.38 | 135,524,603 | 52.27 | 124,894,831 | 49.33 | 129,312,773 | 49.49 |
| Tandem repeat | | 15,989,858 | 6.30 | 22,459,590 | 8.66 | 22,076,382 | 8.72 | 23,329,215 | 8.93 |
| Interpersed repeat | DNA | 18,361,411 | 7.24 | 15,981,589 | 6.16 | 12,252,517 | 4.84 | 13,258,226 | 5.07 |
|  | LINE | 1,416,193 | 0.56 | 1,862,902 | 0.72 | 871,492 | 0.34 | 1,107,613 | 0.42 |
|  | SINE | 829,662 | 0.33 | 775,204 | 0.3 | 93,895 | 0.04 | 348,390 | 0.13 |
|  | LTR | 90,329,408 | 35.61 | 110,021,657 | 42.44 | 105,687,225 | 41.75 | 109,368,919 | 41.86 |
|  | Unknown | 15,112,907 | 5.96 | 12,241,106 | 4.72 | 9,355,702 | 3.7 | 9,277,408 | 3.55 |

During the whole genome sequence, we identified 122.71, 135.52, 124.89, and 129.31 Mb repeat sequences using two approaches, De novo and homologous prediction, which represents 48.38%, 52.27%, 49.33%, and 49.49% of the *P. mira*, *P. davidiana*, *P. kansuensis*, and *P. ferganensis* genome. The value showed higher than that of *P. persica* (37.14%, International Peach Genome Initiative, 2013), Kiwifruit (36%, Huang et al., 2013), and grape (41.4%, The French–Italian Public Consortium for Grapevine Genome Characterization, 2007) but lower than that of other fruit crops, such as apple (67.4%, Velasco et al., 2010), pear (53.1%, Wu et al., 2013), and soybean (53.9%, Xie et al., 2019).

**Supplementary Table 7** Prediction of protein-coding genes in the genomes of four wild peach species.

| Method used for gene annotation | | *P. mira* | *P. davidiana* | *P. kansuensis* | *P. ferganensis* |
| --- | --- | --- | --- | --- | --- |
| De novo | Augustus | 25,140 | 20,761 | 20,893 | 23,887 |
|  | Glimmer HMM | 39,964 | 33,272 | 32,832 | 39,724 |
|  | SNAP | 31,084 | 23,553 | 24,465 | 30,277 |
|  | Geneid | 30,935 | 28,715 | 29,441 | 31,372 |
|  | Genscan | 20,435 | 18,819 | 19,380 | 20,083 |
| Homolog | *P. persica* | 25,134 | 25,147 | 24,966 | 25,240 |
|  | *Pyrus bretschneideri* | 22,675 | 23,363 | 21,182 | 22,157 |
|  | *P. mume* | 23,540 | 22,879 | 22,517 | 23,037 |
|  | *Malus domestica* | 27,658 | 27,799 | 27,377 | 26,390 |
|  | *Fragaria vesca* | 21,790 | 21,391 | 20,951 | 21,908 |
|  | *Vitis vinifera* | 24,119 | 23,482 | 23,417 | 23,124 |
|  | *Arabidopsis thaliana* | 25,717 | 25,124 | 25,514 | 25,150 |
| RNA-seq | PASA | 64,099 | 97,570 | 58,147 | 63,129 |
|  | Transcripts | 31,421 | 30,312 | 32,295 | 31,951 |
| Final set | | 28,519 | 27,236 | 26,986 | 28,587 |

Note: In the study, five software programs were used for gene structure *de novo* annotation, such as Augustus, Glimmer HMM, SNAP, Genscan, and Geneid. Meanwhile, *P. persica*, *Pyrus bretschneideri*, *P. mume*, *Malus domestica*, *Fragaria vesca*, *Vitis vinifera*, and *Arabidopsis thaliana* were used for gene annotation by homolog analysis. In addition, RNA-seq data of different tissues were also used for assistance annotation with different tools, such as PASA and Cufflinks.

**Supplementary Table 8** Statistics of predicted protein-coding genes in four wild peach species compared to other species.

| Species | Number | Average transcript length (bp) | Average CDS length (bp) | Average exons per gene | Average exon length (bp) | Average intron length (bp) |
| --- | --- | --- | --- | --- | --- | --- |
| *P. mira* | 28,519 | 2,804.75 | 1,155.06 | 4.72 | 244.95 | 444.01 |
| *P. davidiana* | 27,236 | 2,797.39 | 1,224.50 | 4.82 | 254.15 | 411.96 |
| *P. kansuensis* | 26,986 | 2,856.05 | 1,207.40 | 4.81 | 250.87 | 432.39 |
| *P. ferganensis* | 28,587 | 2,723.74 | 1,198.20 | 4.71 | 254.61 | 411.63 |
| *P. persica* | 27,861 | 2,445.56 | 1,214.65 | 4.91 | 247.53 | 315.04 |
| *P. dulcis* | 27,040 | 2,790.26 | 1,210.30 | 4.85 | 249.79 | 410.89 |
| *P. mume* | 22,779 | 2,899.32 | 1,346.04 | 5.32 | 252.92 | 359.40 |
| *P. salicina* | 24,447 | 2,988.45 | 1,157.42 | 4.97 | 233.09 | 461.72 |
| *P. armeniaca* | 30,433 | 3,214.54 | 1,257.16 | 4.99 | 252.16 | 491.13 |
| *P. avium* | 38,277 | 2,394.33 | 1,075.31 | 4.46 | 241.10 | 381.21 |
| *Pyrus bretschneideri* | 34,359 | 3,060.72 | 1,335.34 | 5.47 | 244.25 | 386.24 |
| *Malus domestica* | 44,599 | 3,457.46 | 1,182.81 | 5.51 | 214.68 | 504.41 |
| *Fragaria vesca* | 64,597 | 2,996.01 | 1,356.55 | 6.08 | 222.99 | 322.50 |
| *Vitis vinifera* | 26,346 | 5,936.60 | 1,137.11 | 5.95 | 191.10 | 969.55 |
| *Arabidopsis thaliana* | 27,310 | 1,887.74 | 1,229.90 | 5.16 | 238.41 | 158.18 |

The gene transcripts had an average length of 2,445.56 ~ 2,856.05 bp, a mean coding sequence (CDS) size of 1,155.06 ~ 1224.50 bp and an average exon length of 244.95 ~ 254.15 bp in *P. persica* and its four wild related species.

**Supplementary Table 9** Statistics of gene functional annotation in the four wild peach species.

| Database | *P. mira* | | *P. davidiana* | | *P. kansuensis* | | *P. ferganensis* | |
| --- | --- | --- | --- | --- | --- | --- | --- | --- |
|  | Number | Percent (%) | Number | Percent (%) | Number | Percent (%) | Number | Percent (%) |
| Swiss-Prot | 20,486 | 71.83 | 20,294 | 74.51 | 20,313 | 75.27 | 21,093 | 73.79 |
| Nr | 27,493 | 96.40 | 26,495 | 97.28 | 26,296 | 97.44 | 27,721 | 96.97 |
| KEGG | 21,662 | 75.96 | 21,005 | 77.12 | 20,991 | 77.78 | 22,057 | 77.16 |
| InterPro | 25,668 | 90.00 | 24,724 | 90.78 | 24,711 | 91.57 | 25,936 | 90.73 |
| GO | 15,234 | 53.42 | 14,913 | 54.75 | 14,837 | 54.98 | 15,441 | 54.01 |
| Pfam | 21,199 | 74.33 | 20,647 | 75.81 | 20,467 | 75.84 | 21,423 | 74.94 |
| Annotated | 27,613 | 96.82 | 26,545 | 97.46 | 26,374 | 97.73 | 27,803 | 97.26 |
| Total | 28,519 | - | 27,236 | - | 26,986 | - | 28,587 | - |

**Supplementary Table 10** Non-coding RNAs identified in genomes of four wild peach species.

| Type | | *P. mira* | | *P. davidiana* | | *P. kansuensis* | | *P. ferganensis* | |
| --- | --- | --- | --- | --- | --- | --- | --- | --- | --- |
|  |  | Copy | Average length (bp) | Copy | Average length (bp) | Copy | Average length (bp) | Copy | Average length (bp) |
| miRNA | | 693 | 131.85 | 563 | 143.37 | 514 | 146.69 | 529 | 146.53 |
| tRNA | | 1,417 | 75.85 | 1,564 | 75.89 | 1,377 | 75.73 | 1,226 | 75.87 |
| rRNA | Total | 8,171 | 385.52 | 15,770 | 394.59 | 8,539 | 373.96 | 10,633 | 384.19 |
|  | 18S | 1,267 | 1,705.57 | 2,461 | 1,750.29 | 1,244 | 1,736.70 | 1,642 | 1,703.56 |
|  | 28S | 4,767 | 142.46 | 9,495 | 142.34 | 4,708 | 141.79 | 6,180 | 142.56 |
|  | 5.8S | 1,192 | 162.23 | 2,357 | 162.55 | 1,172 | 162.92 | 1,544 | 162.19 |
|  | 5S | 945 | 123.4 | 1,457 | 123.97 | 1,415 | 123.22 | 1,267 | 123.43 |
| snRNA | snRNA | 543 | 124.38 | 518 | 125.19 | 753 | 118.31 | 514 | 121.95 |
|  | CD-box | 385 | 116.51 | 354 | 117.16 | 598 | 111.62 | 355 | 114.55 |
|  | HACA-box | 39 | 133.59 | 40 | 133 | 40 | 136.68 | 38 | 130.39 |
|  | Splicing | 118 | 146.82 | 123 | 145.18 | 113 | 145.54 | 120 | 140.51 |
|  | scaRNA | 1 | 148 | 1 | 193 | 2 | 213.5 | 1 | 203 |

**Supplementary Table 11** SNPs identified between genomes of each of the four wild species and *P. persica*.

| Chromosome | *P. mira* | | *P. davidiana* | | *P. kansuensis* | | *P. ferganensis* | |
| --- | --- | --- | --- | --- | --- | --- | --- | --- |
|  | Number | Density  (bp/SNP) | Number | Density  (bp/SNP) | Number | Density  (bp/SNP) | Number | Density  (bp/SNP) |
| Chr.1 | 640,519 | 74.71 | 609,159 | 78.55 | 526,425 | 90.90 | 78,442 | 610.02 |
| Chr.2 | 363,427 | 83.66 | 350,354 | 86.79 | 298,228 | 101.96 | 100,171 | 303.54 |
| Chr.3 | 362,350 | 75.53 | 350,680 | 78.04 | 317,195 | 86.28 | 54,807 | 499.35 |
| Chr.4 | 339,989 | 76.01 | 286,057 | 90.34 | 280,431 | 92.16 | 99,153 | 260.64 |
| Chr.5 | 250,802 | 73.75 | 251,429 | 73.57 | 225,115 | 82.17 | 37,606 | 491.85 |
| Chr.6 | 408,825 | 75.26 | 402,060 | 76.52 | 343,270 | 89.63 | 67,139 | 458.26 |
| Chr.7 | 296,738 | 75.45 | 301,251 | 74.32 | 265,817 | 84.23 | 50,209 | 445.91 |
| Chr.8 | 298,697 | 75.57 | 299,258 | 75.43 | 271,796 | 83.05 | 50,401 | 447.89 |
| Others | 10,640 |  | 13,913 |  | 15,659 |  | 10,070 |  |
| Total | 2,971,987 |  | 2,864,161 |  | 2,543,936 |  | 547,998 |  |

Note: ‘Others’ indicated the SNPs which not aligned with 8 chromosomes.

**Supplementary Table 12** Small indels (<50 bp) identified between genomes of each of the four wild species and *P. persica*.

| Species | Insertion | | | Deletion | | | Total gene |
| --- | --- | --- | --- | --- | --- | --- | --- |
|  | Length (bp) | Number | Affected gene number | Length (bp) | Number | Affected gene number |  |
| *P. mira* | 1,181,406 | 252,705 | 3,220 | 1,088,234 | 237,716 | 3,180 | 5,452 |
| *P. davidiana* | 1,125,572 | 240,039 | 3,015 | 1,012,328 | 227,990 | 2,955 | 5,073 |
| *P. kansuensis* | 972,355 | 211,569 | 2,581 | 983,299 | 210,538 | 2,648 | 4,506 |
| *P. ferganensis* | 240,557 | 54,781 | 822 | 228,480 | 51,655 | 758 | 1,341 |

**Supplementary Table 13** Structural variants (≥ 50 bp) between the four wild species and *P. persica*.

| Types | | *P. mira* | *P. davidiana* | *P. kansuensis* | *P. ferganensis* |
| --- | --- | --- | --- | --- | --- |
| Inertion | Length (bp) | 1,738,918 | 1,420,361 | 1,902,485 | 953,659 |
|  | Number | 2,985 | 2,667 | 2,548 | 904 |
|  | Affected gene number | 1,743 | 1,497 | 1,454 | 483 |
| Deletion | Length (bp) | 1,222,483 | 1,219,308 | 1,152,917 | 602,239 |
|  | Number | 2,170 | 2,159 | 2,074 | 802 |
|  | Affected gene number | 1,361 | 1,405 | 1,331 | 439 |
| Tandem expansion | Length (bp) | 652,696 | 677,170 | 436,173 | 521,968 |
|  | Number | 200 | 203 | 172 | 227 |
|  | Affected gene number | 171 | 160 | 147 | 120 |
| Tandem contraction | Length (bp) | 23,527 | 24,445 | 31,247 | 66,149 |
|  | Number | 25 | 32 | 26 | 68 |
|  | Affected gene number | 18 | 28 | 27 | 22 |
| Repeat expansion | Length (bp) | 5,446,439 | 4,651,448 | 5,308,701 | 2,215,725 |
|  | Number | 2,446 | 2,161 | 2,172 | 971 |
|  | Affected gene number | 3,211 | 2,776 | 2,905 | 1071 |
| Repeat contraction | Length (bp) | 7,802,787 | 7,881,727 | 6,885,589 | 2,174,265 |
|  | Number | 3,168 | 3,078 | 2,705 | 898 |
|  | Affected gene number | 4,442 | 4,471 | 4,135 | 1,086 |
| Total | Length (bp) | 16,886,850 | 15,874,459 | 15,717,112 | 6,534,005 |
|  | Number | 10,994 | 10,300 | 9,697 | 3,870 |
|  | Affected gene number | 10,126 | 9,599 | 9,333 | 3,094 |

**Supplementary Table 14** Statistics of copy number variations between the four wild species and *P. persica.*

| Species | Deletion | | | Duplication | | |
| --- | --- | --- | --- | --- | --- | --- |
|  | Length (bp) | Number | Affected gene number | Length (bp) | Number | Affected gene number |
| *P. mira* | 46,605,900 | 4,624 | 2,484 | 22,404,300 | 2,185 | 3,549 |
| *P. davidiana* | 44,912,500 | 5,886 | 2,047 | 5,369,100 | 954 | 705 |
| *P. kansuensis* | 35,776,800 | 3,841 | 1,713 | 14,856,600 | 1,303 | 1,958 |
| *P. ferganensis* | 15,076,000 | 2,423 | 672 | 6,608,500 | 921 | 354 |

**Supplementary Table 15** A total of 20 SVs which aligned *P. mira* with *P. persica* genome*.*

| Variation number | Reference Chr. | Reference Start (bp) | Reference End (bp) | Target Chr. | Target Start (bp) | Target End (bp) | Size (bp) | Type |
| --- | --- | --- | --- | --- | --- | --- | --- | --- |
| Vd-1 | Pp01 | 5,248,238 | 5,248,304 | Pmi01 | 5,468,096 | 5,468,096 | 66 | Deletion |
| Vd-2 | Pp01 | 25,166,544 | 25,166,609 | Pmi01 | 25,288,014 | 25,288,014 | 65 | Deletion |
| Vd-3 | Pp01 | 35,564,287 | 35,564,360 | Pmi01 | 35,858,331 | 35,858,331 | 73 | Deletion |
| Vd-4 | Pp02 | 5,313,973 | 5,314,027 | Pmi02 | 5,133,879 | 5,133,879 | 54 | Deletion |
| Vd-5 | Pp02 | 25,103,035 | 25,103,086 | Pmi02 | 25,277,554 | 25,277,554 | 51 | Deletion |
| Vd-6 | Pp03 | 25,004,173 | 25,004,223 | Pmi03 | 25,439,338 | 25,439,338 | 50 | Deletion |
| Vd-7 | Pp04 | 5,076,198 | 5,076,253 | Pmi04 | 5,368,870 | 5,368,870 | 55 | Deletion |
| Vd-8 | Pp04 | 16,008,862 | 16,008,940 | Pmi04 | 16,839,912 | 16,839,912 | 78 | Deletion |
| Vd-9 | Pp05 | 15,130,314 | 15,130,375 | Pmi05 | 16,263,242 | 16,263,242 | 61 | Deletion |
| Vd-10 | Pp06 | 5,069,902 | 5,069,970 | Pmi06 | 5,284,316 | 5,284,316 | 68 | Deletion |
| Vd-11 | Pp06 | 25,387,116 | 25,387,167 | Pmi06 | 25,692,278 | 25,692,278 | 51 | Deletion |
| Vd-12 | Pp08 | 5,419,533 | 5,419,585 | Pmi08 | 5,691,489 | 5,691,489 | 52 | Deletion |
| Vi-1 | Pp01 | 22,142,689 | 22,142,689 | Pmi01 | 22,603,712 | 22,603,780 | 68 | Insertion |
| Vi-2 | Pp02 | 10,051,703 | 10,051,703 | Pmi02 | 10,539,301 | 10,539,367 | 66 | Insertion |
| Vi-3 | Pp04 | 10,103,217 | 10,103,217 | Pmi04 | 10,829,812 | 10,829,865 | 53 | Insertion |
| Vi-4 | Pp05 | 18,160,850 | 18,160,850 | Pmi05 | 19,447,850 | 19,447,900 | 50 | Insertion |
| Vi-5 | Pp06 | 10,001,119 | 10,001,119 | Pmi06 | 10,426,411 | 10,426,467 | 56 | Insertion |
| Vi-6 | Pp06 | 20,001,567 | 20,001,567 | Pmi06 | 20,578,595 | 20,578,649 | 54 | Insertion |
| Vi-7 | Pp07 | 20,131,127 | 20,131,127 | Pmi07 | 22,040,284 | 22,040,338 | 54 | Insertion |
| Vi-8 | Pp08 | 10,171,823 | 10,171,823 | Pmi08 | 10,279,118 | 10,279,171 | 53 | Insertion |

**Supplementary Table 16** The primers which designed to amplify the SVs between *P. mira* and *P. persica* genome*.*

| Primer accession | Forward primer (5-3) | Reverse primer (5-3) |
| --- | --- | --- |
| Vd-1 | CGATCCGGAGTGGATTGGAG | CCAACCAGAGAGGTCAGTTCC |
| Vd-2 | AGTGGACAAAAGGGGAGTGG | CTGATGACCTTGCCTGAGTGA |
| Vd-3 | GTTTGGGCTCGTTCTTGTCG | TCCCCAAAACAAGCAGGACT |
| Vd-4 | GGAGAGGCACTTCAACACCT | AGAGAAAAAGCAGTAAAAGCGG |
| Vd-5 | AGGGCCAAACGGCTAATTCA | GCTTCAGCTTCCTCTCCCTG |
| Vd-6 | TTTCTCACACTGCCGAACCT | TTTACAGCGCCAGATCAAGG |
| Vd-7 | TGGCCAAAAATGGCAGCATC | CCTTTGACTGAGTGCCGAGT |
| Vd-8 | GCGTTACAGGGAGGGCTCTA | AGAAACGTGTGCTTGCTGGA |
| Vd-9 | CACCATTGACAATCATGGCCG | TGCCACCAAACTTGAGGCTT |
| Vd-10 | TGGTTGTCGGTTTTATGCCA | GAGTAATGCTAGGGAGACCACA |
| Vd-11 | GCTGTTAGATCGACTGGTTCAC | CATGGCCCTCAATTTCCCAG |
| Vd-12 | TTTCCGCCATCAACGCTCTC | TGGAAGCACAAACCTGGAAC |
| Vi-1 | GCCTCTCTAACATGGGCTTT | TTGGGGCGAATTTGGTTAAGG |
| Vi-2 | GGGGCGCAAAACAGAAAAGT | TGCCAACATGCACTCGTTTC |
| Vi-3 | GAGCGATGGGACTTGCCTAA | TGTTGTTGTGCTGCGTTGTT |
| Vi-4 | CGTTTAAGGTCCCATGCCATT | GACGGTGACCTTTCTGGTGA |
| Vi-5 | CCTACTGTGGTTGCTGCGTT | CCGCAAACCAAATGTCGGAA |
| Vi-6 | GCGTGGGAGATATTGGGTGA | ATTTTCCTTTCGTGTTGAGCCG |
| Vi-7 | AGAGGACAATCTTGGCAGACC | AATCGCTCTTCTCTGCTCCTT |
| Vi-8 | TTTTTGCATCTCACCGCCCA | CCTAGGCCAGTAAGCGATTGT |

**Supplementary Table 17** Statistics of resistance genes in the four wild peach species.

| Categories | | *P. mira* | *P. davidiana* | *P. kasuensis* | *P. ferganensis* |
| --- | --- | --- | --- | --- | --- |
| CNL | CC-NBS | 5 | 12 | 8 | 8 |
|  | CC-NBS-LRR | 54 | 55 | 45 | 72 |
| TNL | TIR-NBS | 10 | 23 | 14 | 15 |
|  | TIR-NBS-LRR | 82 | 103 | 86 | 80 |
| Unclassified | NBS | 43 | 41 | 35 | 38 |
|  | NBS-LRR | 99 | 107 | 121 | 108 |
|  | LRR-NBS | 1 | 0 | 0 | 0 |
|  | TIR-CC-NBS-LRR | 1 | 0 | 1 | 0 |
|  | TIR-NBS-LRR-TIR-NBS-LRR | 0 | 0 | 0 | 1 |
| Total genes | | 295 | 341 | 310 | 322 |

**Supplementary Table 18** List of 175 peach samples used in the study.

| Accession No. | Accession name | Systematic name | Origin | Population | Geographic groups of landraces | Breeding lines of improved varieties group | Linalool content in 2015 (peak area) | Linalool content in 2016 (peak area) |
| --- | --- | --- | --- | --- | --- | --- | --- | --- |
| 1 | Zao Shu Huang Gan | *P. persica* | Gansu, PRC | Edible landrace | Northwest China |  | 17761 | 311374 |
| 2 | Kashi Huang Rou Li Guang | *P. persica* | Sinkiang, PRC | Edible landrace | Northwest China |  | 12575595 | 2903576 |
| 3 | Jin Ta Hong Guang | *P. persica* | Gansu, PRC | Edible landrace | Northwest China |  |  |  |
| 4 | Jin Ta You Pan Tao | *P. persica* | Gansu, PRC | Edible landrace | Northwest China |  |  |  |
| 5 | Qing Si | *P. persica* | Yunnan, PRC | Edible landrace | YunGui plateau |  |  |  |
| 6 | Huo Lian Jin Dan | *P. persica* | Yunnan, PRC | Edible landrace | YunGui plateau |  | 125661 | 4902499 |
| 7 | Xiao Jin Dan | *P. persica* | Yunnan, PRC | Edible landrace | YunGui plateau |  | 226748 | 357490 |
| 8 | Yilixian Huang Rou Tao | *P. persica* | Sinkiang, PRC | Edible landrace | Northwest China |  |  |  |
| 9 | Maoshan Gong Tao | *P. persica* | Chongqing, PRC | Edible landrace | The middle and lower reaches of the Changjiang River |  |  |  |
| 10 | Long 1-2-4 | *P. persica* | Gansu, PRC | Edible landrace | Northwest China |  |  |  |
| 11 | Chen Pu Pan Tao | *P. persica* | Shanghai, PRC | Edible landrace | The middle and lower reaches of the Changjiang River |  | 1420067 | 420556 |
| 12 | Sa Hua Hong Pan Tao | *P. persica* | Shanghai, PRC | Edible landrace | The middle and lower reaches of the Changjiang River |  | 2131240 | 3140878 |
| 13 | Huang Jin Pan Tao | *P. persica* | Shanghai, PRC | Edible landrace | The middle and lower reaches of the Changjiang River |  | 318271 | 2035304 |
| 14 | Wanzhou Suan Tao | *P. persica* | Chongqing, PRC | Edible landrace | The middle and lower reaches of the Changjiang River |  | 0 | 0 |
| 15 | Nanshan Tian Tao | *P. persica* | Guangdong, PRC | Edible landrace | Southern China |  | 18444 | 123829 |
| 16 | Ying Ge Tao | *P. persica* | Taiwan, PRC | Edible landrace | Southern China |  | 9286 | 861149 |
| 17 | Tianjin Shui Mi | *P. persica* | Tianjin, PRC | Edible landrace | Northern China |  | 0 | 149513 |
| 18 | Wu Hei Ji Rou Tao | *P. persica* | Anhui, PRC | Edible landrace | The middle and lower reaches of the Changjiang River |  |  |  |
| 19 | Jilin 8903# | *P. persica* | Jilin, PRC | Edible landrace | Northeast China |  |  |  |
| 20 | Ping Bei Zi | *P. persica* | Jiangsu, PRC | Edible landrace | The middle and lower reaches of the Changjiang River |  | 11632 | 395914 |
| 21 | Shandong Si Yue Ban | *P. persica* | Shandong, PRC | Edible landrace | Northern China |  | 8854 | 24646 |
| 22 | Hong Ya Zui | *P. persica* | Hebei, PRC | Edible landrace | Northern China |  | 24873 | 1071185 |
| 23 | Da Hong Pao | *P. persica* | Hubei, PRC | Edible landrace | The middle and lower reaches of the Changjiang River |  | 6752 | 150001 |
| 24 | Diao Zhi Bai | *P. persica* | Anhui, PRC | Edible landrace | The middle and lower reaches of the Changjiang River |  | 46830 | 312968 |
| 25 | Xiao Bai Tao | *P. persica* | Henan, PRC | Edible landrace | Northern China |  |  |  |
| 26 | Ge Gu | *P. persica* | Hebei, PRC | Edible landrace | Northern China |  | 0 | 0 |
| 27 | Ying Zui | *P. persica* | Anhui, PRC | Edible landrace | The middle and lower reaches of the Changjiang River |  | 174712 | 46303 |
| 28 | Hun Chun Tao | *P. persica* | Jilin, PRC | Edible landrace | Northeast China |  |  |  |
| 29 | Jilin 8501# | *P. persica* | Jilin, PRC | Edible landrace | Northeast China |  |  |  |
| 30 | Jilin 8601# | *P. persica* | Jilin, PRC | Edible landrace | Northeast China |  |  |  |
| 31 | Bai Li Hu | *P. persica* | Yunnan, PRC | Edible landrace | YunGui plateau |  |  |  |
| 32 | Qing Tao | *P. persica* | Guizhou, PRC | Edible landrace | YunGui plateau |  | 4908662 | 2529296 |
| 33 | Xian Tao | *P. persica* | Guangxi, PRC | Edible landrace | Southern China |  |  |  |
| 34 | Xingyi Wu YueTao | *P. persica* | Guizhou, PRC | Edible landrace | YunGui plateau |  |  |  |
| 35 | Li He Tian Ren | *P. persica* | Sinkiang, PRC | Edible landrace | Northwest China |  |  |  |
| 36 | Kashi 4# | *P. persica* | Sinkiang, PRC | Edible landrace | Northwest China |  | 743861 | 9107126 |
| 37 | Fertilia Morettini | *P. persica* | Italy | Improved variety |  | Europe | 0 | 0 |
| 38 | Croce Decsus | *P. persica* | America | Improved variety |  | America |  |  |
| 39 | Flordadawn | *P. persica* | America | Improved variety |  | America |  | 3397921 |
| 40 | Mai Huang Pan Tao | *P. persica* | Henan, PRC | Improved variety |  | China | 252072 | 348714 |
| 41 | Redhaven | *P. persica* | America | Improved variety |  | America |  | 6942601 |
| 42 | Su Lian Pan Tao | *P. persica* | The Soviet Union | Improved variety |  | Europe | 5486832 | 4400219 |
| 43 | Qian Nian Hong | *P. persica* | Henan, PRC | Improved variety |  | China |  |  |
| 44 | Hua Guang | *P. persica* | Henan, PRC | Improved variety |  | China | 18571 | 115737 |
| 45 | Shu Guang | *P. persica* | Henan, PRC | Improved variety |  | China | 1883842 | 1338614 |
| 46 | Zhong You Tao 5# | *P. persica* | Henan, PRC | Improved variety |  | China | 987171 | 285840 |
| 47 | Shuang Xi Hong | *P. persica* | Henan, PRC | Improved variety |  | China | 395136 | 1835729 |
| 48 | Rui Guang 18# | *P. persica* | Beijing, PRC | Improved variety |  | China | 7218866 | 5339603 |
| 49 | Harmony | *P. persica* | America | Improved variety |  | America | 2421343 | 5601020 |
| 50 | Xia Ye Tao | *P. persica* | Henan, PRC | Improved variety |  | China | 0 | 388333 |
| 51 | May Fire | *P. persica* | America | Improved variety |  | America |  | 2873785 |
| 52 | Nectagrand 1# | *P. persica* | Italy | Improved variety |  | Europe |  |  |
| 53 | Flavortop | *P. persica* | America | Improved variety |  | America | 10520673 | 7028863 |
| 54 | Nectagrand 7# | *P. persica* | Italy | Improved variety |  | Europe |  |  |
| 55 | Okitsu | *P. persica* | Japan | Improved variety |  | Japan and South Korea | 828271 | 604571 |
| 56 | Fuzalode | *P. persica* | France | Improved variety |  | Europe |  |  |
| 57 | Chun Lei | *P. persica* | Shanghai, PRC | Improved variety |  | China |  | 360075 |
| 58 | Okayama Wase | *P. persica* | Japan | Improved variety |  | Japan and South Korea | 78701 | 126791 |
| 59 | Fay Elberta | *P. persica* | America | Improved variety |  | America | 2974665 | 3150119 |
| 60 | Kurakata Wase | *P. persica* | Japan | Improved variety |  | Japan and South Korea | 30182 | 17718 |
| 61 | Denjiulo | *P. persica* | Japan | Improved variety |  | Japan and South Korea | 57098 | 339066 |
| 62 | Hua Yu Lu | *P. persica* | Zhejiang, PRC | Improved variety |  | China | 332336 | 183961 |
| 63 | Jing Yu | *P. persica* | Beijing, PRC | Improved variety |  | China | 283817 | 369576 |
| 64 | Hakuho | *P. persica* | Japan | Improved variety |  | Japan and South Korea | 146052 | 1070275 |
| 65 | Abehakuto | *P. persica* | Japan | Improved variety |  | Japan and South Korea | 90305 | 1923721 |
| 66 | Asama Hakuto | *P. persica* | Japan | Improved variety |  | Japan and South Korea | 79222 | 214284 |
| 67 | Benishimizu | *P. persica* | Japan | Improved variety |  | Japan and South Korea |  |  |
| 68 | Spring prince | *P. persica* | America | Improved variety |  | America | 6401026 | 15336271 |
| 69 | Okubo | *P. persica* | Japan | Improved variety |  | Japan and South Korea |  |  |
| 70 | Zao Shanghai Shui Mi | *P. persica* | Jiangsu, PRC | Improved variety |  | China |  |  |
| 71 | Bai Hua | *P. persica* | Jiangsu, PRC | Improved variety |  | China |  | 64949 |
| 72 | Qin Wang | *P. persica* | Shanxi, PRC | Improved variety |  | China | 22251 | 229752 |
| 73 | Zhong Hua Shou Tao | *P. persica* | Shandong, PRC | Improved variety |  | China |  |  |
| 74 | NJC77 | *P. persica* | America | Improved variety |  | America | 692089 | 5039211 |
| 75 | Babygold 6# | *P. persica* | America | Improved variety |  | America | 189475 | 586312 |
| 76 | Maria Serena | *P. persica* | Italy | Improved variety |  | Europe | 1648405 | 5943410 |
| 77 | Jin Feng | *P. persica* | Henan, PRC | Improved variety |  | China |  | 340267 |
| 78 | Reddomun | *P. persica* | Japan | Improved variety |  | Japan and South Korea | 503517 | 207164 |
| 79 | Kanto 5# | *P. persica* | Japan | Improved variety |  | Japan and South Korea | 2455378 | 375738 |
| 80 | Maria Laura | *P. persica* | Italy | Improved variety |  | Europe |  |  |
| 81 | Ju Hua Tao | *P. persica* | PRC | Ornamental line |  | China |  |  |
| 82 | NJ271 | *P. persica* | America | Ornamental line |  | America |  |  |
| 83 | Fen Shou Xing | *P. persica* | Jiangsu, PRC | Ornamental line |  | China | 27184 | 448052 |
| 84 | S1 | *P. persica* | Beijing, PRC | Ornamental line |  | China |  |  |
| 85 | S2 | *P. persica* | Beijing, PRC | Ornamental line |  | China |  |  |
| 86 | Crimson Cascade | *P. persica* | America | Ornamental line |  | America |  |  |
| 87 | Hong Chui Zhi | *P. persica* | PRC | Ornamental line |  | China |  |  |
| 88 | Hong Ye Tao | *P. persica* | PRC | Ornamental line |  | China |  |  |
| 89 | Tsukuba 86# | *P. persica* | Japan | Wild *P. persica* |  | Japan and South Korea |  |  |
| 90 | Tsukuba 88# | *P. persica* | Japan | Wild *P. persica* |  | Japan and South Korea |  |  |
| 91 | Tsukuba 2# | *P. persica* | Japan | Wild *P. persica* |  | Japan and South Korea |  |  |
| 92 | Tsukuba 3# | *P. persica* | Japan | Wild *P. persica* |  | Japan and South Korea |  |  |
| 93 | Harrow Blood | *P. persica* | Canada | Wild *P. persica* |  | America |  | 5075833 |
| 94 | Siberian C | *P. persica* | Canada | Wild *P. persica* |  | America |  |  |
| 95 | Okinawa | *P. persica* | America | Wild *P. persica* |  | America |  |  |
| 96 | Nemaguard | *P. persica* | America | Wild *P. persica* |  | America |  |  |
| 97 | Chinese Cling | *P. persica* | Shanghai, PRC | Edible landrace | The middle and lower reaches of the Changjiang River |  | 139556 | 593897 |
| 98 | Shenzhou Li He Shui Mi | *P. persica* | Hebei, PRC | Edible landrace | Northern China |  |  |  |
| 99 | Qingzhou Bai Pi Mi Tao | *P. persica* | Shandong, PRC | Edible landrace | Northern China |  |  |  |
| 100 | Fei Cheng Bai Li 10# | *P. persica* | Shandong, PRC | Edible landrace | Northern China |  |  |  |
| 101 | Xinjiang Huang Rou | *P. ferganensis* | Sinkiang, PRC | Edible landrace | Northwest China |  |  |  |
| 102 | Tian Ren Tao | *P. ferganensis* | Sinkiang, PRC | Edible landrace | Northwest China |  |  |  |
| 103 | Liao Xing 1# | *P. mandshurica* | Jilin, PRC | Wild relatives |  |  |  |  |
| 104 | Li He | *P. salicina* | Jilin, PRC | Wild relatives |  |  |  |  |
| 105 | Luo Tuo Huang | *P. armeniaca* | Beijing, PRC | Wild relatives |  |  |  |  |
| 106 | B1-10-2-1 | *P. sibirica* | Liaoning, PRC | Wild relatives |  |  |  |  |
| 107 | Wusuli Li 1# | *P. ussuriensis* | Heilongjiang, PRC | Wild relatives |  |  |  |  |
| 108 | Xinjiang Ying Tao Li | *P. cerasifera* | Sinkiang, PRC | Wild relatives |  |  |  |  |
| 109 | Xinjiang Kao Cha Bian Tao 3# | *P. dulcis* | Sinkiang, PRC | Wild relatives |  |  |  |  |
| 110 | Tao Xing Bian Tao | *P. dulcis* | Sinkiang, PRC | Wild relatives |  |  |  |  |
| 111 | Lawu Cun 3# | *P. mira* | Tibet, PRC | Wild relatives |  |  |  |  |
| 112 | Yuxu Xiang 4# | *P. mira* | Tibet, PRC | Wild relatives |  |  |  |  |
| 113 | Runa Cun 1# | *P. mira* | Tibet, PRC | Wild relatives |  |  |  |  |
| 114 | Brooks | *P. avium* | America | Wild relatives |  |  |  |  |
| 115 | Mahali | *P. mahaleb* | Europe | Wild relatives |  |  |  |  |
| 116 | Mazzard | *P. avium* | Europe | Wild relatives |  |  |  |  |
| 117 | Gan Su Tao (Hong Jing) | *P. kansuensis* | Gansu, PRC | Wild relatives |  |  |  |  |
| 118 | Xi Kang Bian Tao 1# | *P. tangutica* | PRC | Wild relatives |  |  |  |  |
| 119 | Bai Hua Shan Tao 1# | *P. davidiana* | PRC | Wild relatives |  |  |  |  |
| 120 | Hong Hua Shan Tao 1# | *P. davidiana* | Liaoning, PRC | Wild relatives |  |  |  |  |
| 121 | Shan Gan Shan Tao 1# | *P. davidiana* var. *potaninii* | PRC | Wild relatives |  |  |  |  |
| 122 | Bai Gen Gan Su Tao 1# | *P. kansuensis* | Gansu, PRC | Wild relatives |  |  |  |  |
| 123 | 2010-138 | *P. mira* | Tibet, PRC | Wild relatives |  |  |  |  |
| 124 | Hong Gen Gan Su Tao 1# | *P. kansuensis* | Gansu, PRC | Wild relatives |  |  |  |  |
| 125 | Kashi 1# | *P. ferganensis* | Sinkiang, PRC | Edible landrace | Northwest China |  |  |  |
| 126 | Zhou Xing Shan Tao 1# | *P. davidiana* | PRC | Wild relatives |  |  |  |  |
| 127 | 2010xizang 15# | *P. mira* | Tibet, PRC | Wild relatives |  |  |  |  |
| 128 | 2010xizang 17# | *P. mira* | Tibet, PRC | Wild relatives |  |  |  |  |
| 129 | 2010xizang 26# | *P. mira* | Tibet, PRC | Wild relatives |  |  |  |  |
| 130 | 2010xizang 28# | *P. mira* | Tibet, PRC | Wild relatives |  |  |  |  |
| 131 | 2010xizang 29# | *P. mira* | Tibet, PRC | Wild relatives |  |  |  |  |
| 132 | 2010xizang 32# | *P. mira* | Tibet, PRC | Wild relatives |  |  |  |  |
| 133 | 2010xizang 54# | *P. mira* | Tibet, PRC | Wild relatives |  |  |  |  |
| 134 | 2010xizang 55# | *P. mira* | Tibet, PRC | Wild relatives |  |  |  |  |
| 135 | 2010xizang 57# | *P. mira* | Tibet, PRC | Wild relatives |  |  |  |  |
| 136 | Daze 2# | *P. mira* | Tibet, PRC | Wild relatives |  |  |  |  |
| 137 | Guanghetao 001# | *P. mira* | Tibet, PRC | Wild relatives |  |  |  |  |
| 138 | Guanghetao 002# | *P. mira* | Tibet, PRC | Wild relatives |  |  |  |  |
| 139 | Guanghetao 003# | *P. mira* | Tibet, PRC | Wild relatives |  |  |  |  |
| 140 | Guanghetao 004# | *P. mira* | Tibet, PRC | Wild relatives |  |  |  |  |
| 141 | Guanghetao 008# | *P. mira* | Tibet, PRC | Wild relatives |  |  |  |  |
| 142 | Guanghetao 012# | *P. mira* | Tibet, PRC | Wild relatives |  |  |  |  |
| 143 | Guanghetao 013# | *P. mira* | Tibet, PRC | Wild relatives |  |  |  |  |
| 144 | Guanghetao 015# | *P. mira* | Tibet, PRC | Wild relatives |  |  |  |  |
| 145 | Guanghetao 017# | *P. mira* | Tibet, PRC | Wild relatives |  |  |  |  |
| 146 | Guanghetao 025# | *P. mira* | Tibet, PRC | Wild relatives |  |  |  |  |
| 147 | Guanghetao 026# | *P. mira* | Tibet, PRC | Wild relatives |  |  |  |  |
| 148 | Guanghetao 027# | *P. mira* | Tibet, PRC | Wild relatives |  |  |  |  |
| 149 | Guanghetao 028# | *P. mira* | Tibet, PRC | Wild relatives |  |  |  |  |
| 150 | Guanghetao 029# | *P. mira* | Tibet, PRC | Wild relatives |  |  |  |  |
| 151 | Guanghetao 031# | *P. mira* | Tibet, PRC | Wild relatives |  |  |  |  |
| 152 | Guanghetao 032# | *P. mira* | Tibet, PRC | Wild relatives |  |  |  |  |
| 153 | Guanghetao 033# | *P. mira* | Tibet, PRC | Wild relatives |  |  |  |  |
| 154 | Guanghetao 034# | *P. mira* | Tibet, PRC | Wild relatives |  |  |  |  |
| 155 | Guanghetao 035# | *P. mira* | Tibet, PRC | Wild relatives |  |  |  |  |
| 156 | Guanghetao 039# | *P. mira* | Tibet, PRC | Wild relatives |  |  |  |  |
| 157 | Guanghetao 040# | *P. mira* | Tibet, PRC | Wild relatives |  |  |  |  |
| 158 | Guanghetao 042# | *P. mira* | Tibet, PRC | Wild relatives |  |  |  |  |
| 159 | Guanghetao 051# | *P. mira* | Tibet, PRC | Wild relatives |  |  |  |  |
| 160 | Guanghetao 052# | *P. mira* | Tibet, PRC | Wild relatives |  |  |  |  |
| 161 | Guanghetao 053# | *P. mira* | Tibet, PRC | Wild relatives |  |  |  |  |
| 162 | Guanghetao 054# | *P. mira* | Tibet, PRC | Wild relatives |  |  |  |  |
| 163 | Guanghetao 055# | *P. mira* | Tibet, PRC | Wild relatives |  |  |  |  |
| 164 | Guanghetao 057# | *P. mira* | Tibet, PRC | Wild relatives |  |  |  |  |
| 165 | Guanghetao (Aba) | *P. mira* | Tibet, PRC | Wild relatives |  |  |  |  |
| 166 | Guanghetao (Rikaza) | *P. mira* | Tibet, PRC | Wild relatives |  |  |  |  |
| 167 | Lawucun 1# | *P. mira* | Tibet, PRC | Wild relatives |  |  |  |  |
| 168 | Lawucun 2# | *P. mira* | Tibet, PRC | Wild relatives |  |  |  |  |
| 169 | Lawucun 4# | *P. mira* | Tibet, PRC | Wild relatives |  |  |  |  |
| 170 | Runacun 1# | *P. mira* | Tibet, PRC | Wild relatives |  |  |  |  |
| 171 | Tongmai 2# | *P. mira* | Tibet, PRC | Wild relatives |  |  |  |  |
| 172 | Yuxu 1# | *P. mira* | Tibet, PRC | Wild relatives |  |  |  |  |
| 173 | Yuxu 2# | *P. mira* | Tibet, PRC | Wild relatives |  |  |  |  |
| 174 | Yuxu 3# | *P. mira* | Tibet, PRC | Wild relatives |  |  |  |  |
| 175 | Yuxu 4# | *P. mira* | Tibet, PRC | Wild relatives |  |  |  |  |

**Supplementary Table 19** The summary statistics of genome resequencing.

| Accession No. | Average sequencing depth (×) | Coverage (%) | SRA accessions |  | Accession No. | Average sequencing depth (×) | Coverage (%) | SRA accessions |
| --- | --- | --- | --- | --- | --- | --- | --- | --- |
| 1 | 43.61 | 98.1 | SRX4994085 |  | **91** | 52.35 | 98.91 | SRX5118005 |
| 2 | 44.15 | 99.04 | SRX8233324 |  | **92** | 46.92 | 98.71 | SRX5118006 |
| 3 | 45.73 | 98.39 | SAMN15501488 |  | **93** | 51.07 | 98.87 | SRX8233361 |
| 4 | 46.05 | 98.03 | SRX8233346 |  | **94** | 44.53 | 97.43 | SRX2914753 |
| 5 | 54.14 | 98.3 | SRX2914803 |  | **95** | 54.07 | 97.42 | SRX2914754 |
| 6 | 54.86 | 98.35 | SRX4994096 |  | **96** | 47.83 | 98.27 | SRX2914755 |
| 7 | 51.69 | 98.12 | SRX2914806 |  | **97** | 51.06 | 98.34 | SRX4994080 |
| 8 | 55.08 | 97.81 | SAMN15501456 |  | **98** | 51.93 | 99.05 | SRX2914889 |
| 9 | 43.55 | 98.73 | SAMN15501476 |  | **99** | 50.61 | 97.57 | SRX8233292 |
| 10 | 47.89 | 97.96 | SRX8233350 |  | **100** | 47.11 | 98.18 | SRX4994104 |
| 11 | 49.21 | 97.97 | SRX8233147 |  | **101** | 51.61 | 97.64 | SRX4994106 |
| 12 | 54.72 | 97.99 | SRX8233325 |  | **102** | 55.02 | 97.64 | SRX4994111 |
| 13 | 54.46 | 98.32 | SRX8233287 |  | **103** | 39.18 | 78.52 | SAMN15501425 |
| 14 | 50.34 | 98.66 | SRX2914974 |  | **104** | 34.35 | 76.85 | SAMN15501426 |
| 15 | 43.51 | 97.68 | SRX4994088 |  | **105** | 44.26 | 78.92 | SAMN15501417 |
| 16 | 43.56 | 99.37 | SRX4994089 |  | **106** | 34.23 | 76.8 | SAMN15501427 |
| 17 | 46.3 | 97.7 | SRX4994082 |  | **107** | 32.42 | 76.48 | SAMN15501428 |
| 18 | 45.81 | 97.34 | SRX8233356 |  | **108** | 39.18 | 78.09 | SAMN15501420 |
| 19 | 45.49 | 98.49 | SRX4994110 |  | **109** | 34.01 | 89.52 | SAMN15501422 |
| 20 | 48.46 | 98.47 | SRX8233352 |  | **110** | 34.49 | 98.85 | SAMN15501421 |
| 21 | 51.95 | 97.99 | SRX8233263 |  | **111** | 39.82 | 90.92 | SAMN15501442 |
| 22 | 46.05 | 98.48 | SRX8233330 |  | **112** | 33.94 | 90.3 | SAMN15501444 |
| 23 | 43.2 | 98.59 | SRX8233355 |  | **113** | 40.72 | 92.45 | SAMN15501443 |
| 24 | 53.24 | 98.61 | SRX8233284 |  | **114** | 32.49 | 71.39 | SAMN15501418 |
| 25 | 47.31 | 98.75 | SRX2914898 |  | **115** | 41.01 | 72.53 | SAMN15501424 |
| 26 | 42.86 | 98.2 | SRX2914905 |  | **116** | 34.33 | 71.59 | SAMN15501419 |
| 27 | 49.2 | 98.55 | SRX4994081 |  | **117** | 58.56 | 94.47 | SAMN15501423 |
| 28 | 50.5 | 97.3 | SRX4994109 |  | **118** | 48.77 | 91.98 | SRX5117991 |
| 29 | 55.94 | 98.24 | SRX4994103 |  | **119** | 52.04 | 93.03 | SRX8233065 |
| 30 | 53.02 | 97.39 | SAMN15501489 |  | **120** | 47.54 | 92.45 | SRX8233154 |
| 31 | 60.89 | 98.64 | SRX2914784 |  | **121** | 52.66 | 93.86 | SRX8233176 |
| 32 | 53.3 | 98.7 | SRX4994095 |  | **122** | 49.4 | 93.07 | SRX8233087 |
| 33 | 45.76 | 97.19 | SRX4994087 |  | **123** | 36.1 | 88.85 | SRX2915006 |
| 34 | 56.52 | 98.74 | SAMN15501454 |  | **124** | 53.69 | 92.24 | SAMN15501398 |
| 35 | 49.28 | 97.63 | SRX8233362 |  | **125** | 47.69 | 97.49 | SRX8233329 |
| 36 | 53.57 | 97.81 | SRX2914839 |  | **126** | 52.95 | 92.54 | SRX8233076 |
| 37 | 44.32 | 99.47 | SRX2914602 |  | **127** | 6.15 | 93.5 | SRX2915027 |
| 38 | 47.95 | 98.52 | SRX2914757 |  | **128** | 6.46 | 94.17 | SRX2915028 |
| 39 | 50.91 | 98.99 | SRX5118026 |  | **129** | 6.37 | 93.38 | SRX2915029 |
| 40 | 49.12 | 98.99 | SRX8233322 |  | **130** | 6.74 | 90.29 | SRX2915030 |
| 41 | 47.11 | 99.41 | SRX4994101 |  | **131** | 6.97 | 86.96 | SRX2915031 |
| 42 | 43.16 | 99.5 | SRX2914730 |  | **132** | 6.71 | 91.66 | SRX2915032 |
| 43 | 53.63 | 99.04 | SRX2914731 |  | **133** | 6.49 | 92.36 | SRX2915033 |
| 44 | 45.08 | 99.3 | SRX8233258 |  | **134** | 6.5 | 91.6 | SRX2915034 |
| 45 | 43.8 | 99.36 | SRX8233248 |  | **135** | 7.1 | 92.69 | SRX2915035 |
| 46 | 52.35 | 99.2 | SRX1007913 |  | **136** | 8.13 | 95.95 | SRX2915020 |
| 47 | 45.13 | 99.39 | SRX8233255 |  | **137** | 37.8 | 97.31 | SRX5117995 |
| 48 | 51.86 | 99.32 | SRX2914739 |  | **138** | 36.91 | 97.01 | SRX5117998 |
| 49 | 52.2 | 99.11 | SRX2914693 |  | **139** | 36.69 | 98.7 | SRX5117999 |
| 50 | 56.1 | 98.57 | SRX5118036 |  | **140** | 40.24 | 97.12 | SRX5117996 |
| 51 | 42.15 | 98.51 | SRX2914574 |  | **141** | 42.99 | 98.68 | SAMN15501495 |
| 52 | 40.77 | 98.92 | SRX2914742 |  | **142** | 34.64 | 98.51 | SAMN15501494 |
| 53 | 44.26 | 99.26 | SRX2914747 |  | **143** | 37.11 | 98.54 | SRX5117997 |
| 54 | 49.54 | 99.19 | SRX2914746 |  | **144** | 35.66 | 97.48 | SAMN20718670 |
| 55 | 54.32 | 98.76 | SRX4994091 |  | **145** | 43.38 | 97.1 | SAMN20718671 |
| 56 | 52.38 | 99.33 | SRX2914632 |  | **146** | 7.51 | 93.74 | SRX5118002 |
| 57 | 49.43 | 98.56 | SRX2914636 |  | **147** | 41.24 | 98.89 | SAMN20718672 |
| 58 | 54.44 | 98.58 | SRX2914650 |  | **148** | 32.52 | 98.02 | SRX5118003 |
| 59 | 43.64 | 99.36 | SRX2914689 |  | **149** | 42.58 | 94.57 | SAMN20718673 |
| 60 | 43.93 | 98.67 | SRX2914656 |  | **150** | 37.41 | 96.89 | SAMN20718674 |
| 61 | 48.66 | 98.47 | SRX2914657 |  | **151** | 35.25 | 97.78 | SRX5118000 |
| 62 | 49.63 | 98.15 | SRX5118038 |  | **152** | 50.11 | 99.09 | SAMN20718675 |
| 63 | 40.01 | 98.93 | SRX4994098 |  | **153** | 38.53 | 98.81 | SAMN15501435 |
| 64 | 50.37 | 98.44 | SRX4994092 |  | **154** | 43.46 | 97.15 | SRX5118001 |
| 65 | 49.75 | 97.71 | SRX2914668 |  | **155** | 38.13 | 99.03 | SAMN15501493 |
| 66 | 46.75 | 98 | SRX2914665 |  | **156** | 44.09 | 97.67 | SRX5118015 |
| 67 | 51.98 | 98.42 | SRX2914667 |  | **157** | 39.56 | 97.73 | SAMN15501492 |
| 68 | 46.75 | 98.1 | SRX2914702 |  | **158** | 7.28 | 93.05 | SAMN20718679 |
| 69 | 47.53 | 98.58 | SRX4994090 |  | **159** | 34.52 | 97.69 | SRX5118014 |
| 70 | 44.64 | 99.54 | SRX8233239 |  | **160** | 37.41 | 97.71 | SRX5118017 |
| 71 | 45.26 | 97.88 | SRX8233333 |  | **161** | 36.78 | 96.73 | SAMN15501490 |
| 72 | 44.65 | 97.59 | SRX2914678 |  | **162** | 32.2 | 96.74 | SAMN20718676 |
| 73 | 44.06 | 97.63 | SRX8233363 |  | **163** | 37.78 | 98.13 | SAMN20718677 |
| 74 | 42.03 | 98.71 | SRX8233366 |  | **164** | 38.86 | 90.36 | SAMN20718678 |
| 75 | 45.24 | 99.31 | SRX2914717 |  | **165** | 7.57 | 95.38 | SRX5118037 |
| 76 | 46.19 | 98.72 | SRX4994099 |  | **166** | 6.27 | 88.95 | SRX4994105 |
| 77 | 55.43 | 99.21 | SRX8233193 |  | **167** | 6.24 | 91.09 | SRX2915000 |
| 78 | 58.37 | 98.21 | SRX2914684 |  | **168** | 6.79 | 94.36 | SRX2915001 |
| 79 | 49.56 | 99.34 | SRX2914720 |  | **169** | 9.3 | 89.78 | SRX2915003 |
| 80 | 64.35 | 98.97 | SRX2914756 |  | **170** | 36.75 | 98.92 | SRX2915012 |
| 81 | 50.58 | 97.91 | SRX8233259 |  | **171** | 7.34 | 93.17 | SRX2915014 |
| 82 | 44.04 | 98.76 | SRX8233205 |  | **172** | 8.79 | 94.03 | SRX2915008 |
| 83 | 50.37 | 97.44 | SRX8233265 |  | **173** | 7.68 | 92.09 | SRX2915009 |
| 84 | 46.87 | 98.37 | SRX5117980 |  | **174** | 6.77 | 96.46 | SRX2915010 |
| 85 | 46.45 | 98.58 | SRX5117979 |  | **175** | 31.18 | 97.26 | SRX2915011 |
| 86 | 52.04 | 98.89 | SRX5117978 |  |  |  |  |  |
| 87 | 42.02 | 97.86 | SRX268370 |  |  |  |  |  |
| 88 | 43.92 | 99.04 | SRX8233204 |  |  |  |  |  |
| 89 | 46.97 | 98.86 | SRX5117982 |  |  |  |  |  |
| 90 | 52.53 | 99.33 | SRX5117981 |  |  |  |  |  |

**Supplementary Table 20** Variations in the promoter and mRNA regions of *R* genes on Chr. 2 (5-7 Mb) those were specific to *P. kansuensis*.

| ***R* gene** | | | **Variations** | | | | |
| --- | --- | --- | --- | --- | --- | --- | --- |
| **Accession name** | Start position (bp) | End position (bp) | Start position (bp) | End position (bp) | Size (bp) | Variation type | Annotation |
| ***Prupe.2G045200*** | 5,054,083 | 5,060,312 | 5,055,163 | 5,055,163 | 1 | Deletion | exonic |
|  |  |  | 5,057,124 | 5,057,129 | 6 | Deletion | intronic |
|  |  |  | 5,022,478 | 5,069,964 | 4901 | Repeat contraction | exonic |
| ***Prupe.2G046000*** | 5,254,014 | 5,257,565 | 5,254,422 | 5,254,422 | 1 | Deletion | exonic |
|  |  |  | 5,257,953 | 5,257,955 | 3 | Deletion | intergenic |
|  |  |  | 5,022,478 | 5,069,964 | 4901 | Repeat contraction | exonic |
| ***Prupe.2G046300*** | 5,308,864 | 5,313,947 | 5,308,138 | 5,308,138 | 1 | Deletion | upstream |
| ***Prupe.2G046400*** | 5,370,808 | 5,377,086 | 5,376,152 | 5,376,152 | 1 | Deletion | downstream |
| ***Prupe.2G046700*** | 5,408,587 | 5,413,667 | 5,408,165 | 5,408,165 | 1 | Deletion | upstream;downstream |
| ***Prupe.2G046900*** | 5,445,798 | 5,452,769 | 5,445,997 | 5,445,997 | 1 | Deletion | five_prime_UTR |
|  |  |  | 5,447,315 | 5,447,320 | 6 | Deletion | exonic |
|  |  |  | 5,451,461 | 5,451,464 | 4 | Deletion | intronic |
|  |  |  | 5,451,702 | 5,451,707 | 6 | Deletion | intronic |
|  |  |  | 5,452,259 | 5,452,259 | 1 | Deletion | 3'UTR |
|  |  |  | 5,430,139 | 5,446,746 | 1741 | Repeat contraction | 5'UTR+upstream |
|  |  |  | 5,451,242 | 5,460,975 | 8938 | Repeat contraction | instron+exonic+3'UTR+downstream |
| ***Prupe.2G051100*** | 5,902,195 | 5,905,326 |  |  |  |  |  |
| ***Prupe.2G052200*** | 6,060,321 | 6,064,679 |  |  |  |  |  |
| ***Prupe.2G053600*** | 6,212,618 | 6,217,340 | 6,212,387 | 6,212,399 | 13 | Deletion | upstream |
|  |  |  | 6,212,403 | 6,212,422 | 20 | Deletion | upstream |
|  |  |  | 6,212,424 | 6,212,425 | 2 | Deletion | upstream |
|  |  |  | 6,217,200 | 6,217,201 | 2 | Deletion | 3'UTR |
| ***Prupe.2G053700*** | 6,258,789 | 6,263,801 | 6,241,077 | 6,292,582 | 2472 | Repeat contraction | exonic |
| ***Prupe.2G053800*** | 6,279,090 | 6,292,484 | 6,277,608 | 6,277,619 | 12 | Deletion | upstream |
|  |  |  | 6,277,871 | 6,277,871 | 1 | Deletion | upstream |
|  |  |  | 6,277,875 | 6,277,875 | 1 | Deletion | upstream |
|  |  |  | 6,277,877 | 6,277,877 | 1 | Deletion | upstream |
|  |  |  | 6,277,879 | 6,277,879 | 1 | Deletion | upstream |
|  |  |  | 6,277,882 | 6,277,883 | 2 | Deletion | upstream |
|  |  |  | 6,277,891 | 6,277,891 | 1 | Deletion | upstream |
|  |  |  | 6,277,896 | 6,277,898 | 3 | Deletion | upstream |
|  |  |  | 6,277,900 | 6,277,900 | 1 | Deletion | upstream |
|  |  |  | 6,277,903 | 6,277,906 | 4 | Deletion | upstream |
|  |  |  | 6,277,914 | 6,277,916 | 3 | Deletion | upstream |
|  |  |  | 6,277,918 | 6,277,918 | 1 | Deletion | upstream |
|  |  |  | 6,278,160 | 6,278,160 | 1 | Deletion | upstream |
|  |  |  | 6,280,799 | 6,280,799 | 1 | Deletion | exonic |
|  |  |  | 6,290,674 | 6,290,674 | 1 | Deletion | intronic |
|  |  |  | 6,290,859 | 6,290,859 | 1 | Deletion | intronic |
|  |  |  | 6,291,194 | 6,291,194 | 1 | Deletion | intronic |
|  |  |  | 6,241,077 | 6,292,582 | 2472 | Repeat contraction | exonic |
|  |  |  | 6,291,457 | 6,292,975 | 8570 | Tandem expansion | intron+3'UTR+downstream |
| ***Prupe.2G053900*** | 6,309,628 | 6,311,069 |  |  |  |  |  |
| ***Prupe.2G054000*** | 6,311,430 | 6,313,309 |  |  |  |  |  |
| ***Prupe.2G054100*** | 6,330,046 | 6,330,862 |  |  |  |  |  |
| ***Prupe.2G054300*** | 6,369,296 | 6,375,443 | 6,375,328 | 6,375,341 | 14 | Deletion | 3'UTR |
| ***Prupe.2G054500*** | 6,428,085 | 6,431,952 | 6,426,304 | 6,426,304 | 1 | Deletion | upstream |
|  |  |  | 6,426,997 | 6,426,997 | 1 | Deletion | upstream |
|  |  |  | 6,427,436 | 6,427,440 | 5 | Deletion | upstream |
| ***Prupe.2G054700*** | 6,460,427 | 6,466,614 |  |  |  |  |  |
| ***Prupe.2G055200*** | 6,542,068 | 6,547,629 | 6,541,428 | 6,541,429 | 2 | Deletion | upstream |
|  |  |  | 6,541,858 | 6,541,858 | 1 | Deletion | upstream |
|  |  |  | 6,542,584 | 6,542,621 | 38 | Deletion | 5'UTR |
|  |  |  | 6,543,587 | 6,543,588 | 2 | Deletion | intronic |
|  |  |  | 6,543,601 | 6,543,601 | 1 | Deletion | intronic |
|  |  |  | 6,545,180 | 6,545,180 | 1 | Deletion | intronic |
|  |  |  | 6,546,624 | 6,546,629 | 6 | Deletion | exonic |
| ***Prupe.2G055500*** | 6,600,515 | 6,601,705 | 6,598,964 | 6,598,964 | 1 | Deletion | upstream |
|  |  |  | 6,599,143 | 6,599,149 | 7 | Deletion | upstream |
|  |  |  | 6,599,676 | 6,599,676 | 1 | Deletion | upstream |
|  |  |  | 6,599,949 | 6,599,950 | 2 | Deletion | upstream |
|  |  |  | 6,599,953 | 6,599,958 | 6 | Deletion | upstream |
|  |  |  | 6,599,961 | 6,599,961 | 1 | Deletion | upstream |
|  |  |  | 6,599,963 | 6,599,963 | 1 | Deletion | upstream |
|  |  |  | 6,600,058 | 6,600,074 | 17 | Deletion | upstream |
|  |  |  | 6,600,585 | 6,600,670 | 86 | Deletion | exonic |
|  |  |  | 6,600,954 | 6,600,954 | 1 | Deletion | exonic |
| ***Prupe.2G055600*** | 6,636,553 | 6,641,016 | 6,637,372 | 6,637,372 | 1 | Deletion | intronic |
|  |  |  | 6,638,972 | 6,638,972 | 1 | Deletion | intronic |
| ***Prupe.2G055700*** | 6,647,990 | 6,652,171 | 6,646,004 | 6,646,005 | 2 | Deletion | upsteam |
|  |  |  | 6,646,583 | 6,646,583 | 1 | Deletion | upsteam |
|  |  |  | 6,646,588 | 6,646,588 | 1 | Deletion | upsteam |
|  |  |  | 6,646,835 | 6,646,837 | 3 | Deletion | upsteam |
|  |  |  | 6,647,049 | 6,647,051 | 3 | Deletion | upsteam |
|  |  |  | 6,647,100 | 6,647,103 | 4 | Deletion | upsteam |
|  |  |  | 6,649,677 | 6,649,679 | 3 | Deletion | exonic |
|  |  |  | 6,650,005 | 6,650,005 | 1 | Deletion | exonic |
|  |  |  | 6,650,923 | 6,650,923 | 1 | Deletion | exonic |
|  |  |  | 6,651,512 | 6,651,519 | 8 | Deletion | intronic |
|  |  |  | 6,652,031 | 6,652,031 | 1 | Deletion | exonic |
|  |  |  | 6,645,178 | 6,650,755 | 264 | Repeat expansion | intron+exonic+upstream |
| ***Prupe.2G056100*** | 6,680,147 | 6,684,535 | 6,678,837 | 6,678,848 | 12 | Deletion | upstream |
|  |  |  | 6,681,306 | 6,681,310 | 5 | Deletion | exonic |
|  |  |  | 6,684,465 | 6,684,468 | 4 | Deletion | 3'UTR |
|  |  |  | 6,684,496 | 6,684,500 | 5 | Deletion | 3'UTR |
| ***Prupe.2G057000*** | 6,859,284 | 6,859,837 | 6,859,898 | 6,859,898 | 1 | Deletion | upstream |
|  |  |  | 6,860,914 | 6,860,914 | 1 | Deletion | upstream |
|  |  |  | 6,861,277 | 6,861,277 | 1 | Deletion | intergenic |
|  |  |  | 6,861,281 | 6,914,800 | 9125 | Repeat expansion | exonic |
| ***Prupe.2G057100*** | 6,901,141 | 6,907,913 | 6,902,955 | 6,902,955 | 1 | Deletion | exonic |
|  |  |  | 6,903,576 | 6,903,576 | 1 | Deletion | exonic |
|  |  |  | 6,904,384 | 6,904,384 | 1 | Deletion | intronic |
|  |  |  | 6,906,046 | 6,906,051 | 6 | Deletion | intronic |
|  |  |  | 6,906,904 | 6,906,931 | 28 | Deletion | intronic |
|  |  |  | 6,907,277 | 6,907,277 | 1 | Deletion | intronic |
|  |  |  | 6,908,345 | 6,908,358 | 14 | Deletion | 5'UTR |
|  |  |  | 6,908,372 | 6,908,377 | 6 | Deletion | 5'UTR |
|  |  |  | 6,908,455 | 6,908,460 | 6 | Deletion | upstream |

**Supplementary Table 21** The cis-elements in promoter region of *Prupe.2G053600* gene in nematode-resistant and susceptible accessions.

| Cis-element | Sequence | The element number in ‘Hong Gen Gan Su Tao’ peach | The element number in ‘Bailey’ peach | Function |
| --- | --- | --- | --- | --- |
| A-box | CCGTCC | 1 | 1 | cis-acting regulatory element |
| AC-I | (T/C)C(T/C)(C/T)ACC(T/C)ACC | 1 | 1 |  |
| AE-box | AGAAACAA | 1 | 1 | part of a module for light response |
| ARE | AAACCA | 2 | 2 | cis-acting regulatory element essential for the anaerobic induction |
| as-1 | TGACG | 3 | 2 |  |
| AT~TATA-box | TATATA | 2 | 2 |  |
| Box 4 | ATTAAT | 1 | 1 | part of a conserved DNA module involved in light responsiveness |
| CAAT-box | CAAAT | 41 | 38 | common cis-acting element in promoter and enhancer regions |
| CAT-box | GCCACT | 2 | 2 | cis-acting regulatory element related to meristem expression |
| CCGTCC motif | CCGTCC | 1 | 1 |  |
| CCGTCC-box | CCGTCC | 1 | 1 |  |
| CGTCA-motif | CGTCA | 3 | 3 | cis-acting regulatory element involved in the MeJA-responsiveness |
| chs-CMA2a | TCACTTGA | 0 | 1 |  |
| CTAG-motif | ACTAGCAGAA | 1 | 2 |  |
| GARE-motif | TCTGTTG | 1 | 1 | gibberellin-responsive element |
| GCN4_motif | TGAGTCA | 1 | 1 | cis-regulatory element involved in endosperm expression |
| GT1-motif | GGTTAA | 3 | 3 | light responsive element |
| LTR | CCGAAA | 1 | 2 | cis-acting element involved in low-temperature responsiveness |
| MBS | CAACTG | 2 | 2 | MYB binding site involved in drought-inducibility |
| MBSI | aaaAaaC(G/C)GTTA | 1 | 1 | MYB binding site involved in flavonoid biosynthetic genes regulation |
| MYB | TAACCA | 14 | 14 |  |
| MYC | CATTTG | 3 | 2 |  |
| STRE | AGGGG | 5 | 7 |  |
| TATA-box | TATA | 29 | 33 | core promoter element around -30 of transcription start |
| TCA-element | TCAGAAGAGG | 1 | 2 | cis-acting element involved in salicylic acid responsiveness |
| TCCC-motif | TCTCCCT | 1 | 1 | part of a light responsive element |
| TGACG-motif | TGACG | 3 | 3 | cis-acting regulatory element involved in the MeJA-responsiveness |
| TGA-element | AACGAC | 2 | 1 | auxin-responsive element |
| W box | TTGACC | 1 | 1 |  |
| WRE3 | CCACCT | 0 | 1 |  |
| WUN-motif | AAATTACT | 1 | 1 |  |

**Supplementary Table 22** Genes selected between the two subgroups of *P. mira* which originated from high- and low-altitude regions.

| Gene ID | Chr. | Start (bp) | End (bp) | Function annotated by NR or Swiss-Prot database |
| --- | --- | --- | --- | --- |
| *Pmi01g1849* | Pmi01 | 15704792 | 15709171 | TATA box-binding protein-associated factor RNA polymerase I subunit B |
| *Pmi01g1850* | Pmi01 | 15710175 | 15717220 | MACPF domain-containing protein At1g14780 |
| *Pmi01g1851* | Pmi01 | 15720062 | 15721783 | Rho GDP-dissociation inhibitor 1 |
| *Pmi01g1852* | Pmi01 | 15723951 | 15736825 | Phosphoglucan, water dikinase, chloroplastic |
| *Pmi01g1853* | Pmi01 | 15765372 | 15770023 | NA |
| *Pmi01g1854* | Pmi01 | 15770487 | 15771110 | NA |
| *Pmi01g1855* | Pmi01 | 15772011 | 15776470 | NA |
| *Pmi01g1856* | Pmi01 | 15776411 | 15777278 | NA |
| *Pmi01g1857* | Pmi01 | 15798102 | 15811790 | NA |
| *Pmi01g1872* | Pmi01 | 16019997 | 16024843 | NA |
| *Pmi01g1873* | Pmi01 | 16026839 | 16028161 | NA |
| *Pmi01g1874* | Pmi01 | 16043649 | 16043836 | NA |
| *Pmi01g1875* | Pmi01 | 16046888 | 16047496 | uncharacterized protein LOC109946549 |
| *Pmi01g1876* | Pmi01 | 16072937 | 16083293 | Fanconi-associated nuclease 1 homolog |
| *Pmi01g1877* | Pmi01 | 16086194 | 16087645 | Protein WHAT'S THIS FACTOR 1 homolog, chloroplastic |
| *Pmi01g1878* | Pmi01 | 16087723 | 16088022 | NA |
| *Pmi01g1879* | Pmi01 | 16092242 | 16097640 | Uncharacterized WD repeat-containing protein C2A9.03 |
| *Pmi01g1880* | Pmi01 | 16105182 | 16108756 | Probably inactive leucine-rich repeat receptor-like protein kinase At3g28040 |
| *Pmi01g1881* | Pmi01 | 16111365 | 16116857 | Eukaryotic translation initiation factor 3 subunit H |
| *Pmi01g1882* | Pmi01 | 16131001 | 16133034 | Amino acid transporter AVT1H |
| *Pmi01g1883* | Pmi01 | 16140336 | 16142194 | CRIB domain-containing protein RIC4 |
| *Pmi01g1884* | Pmi01 | 16144171 | 16149933 | Probable protein phosphatase 2C 33 |
| *Pmi01g2177* | Pmi01 | 19594210 | 19603931 | NA |
| *Pmi01g2178* | Pmi01 | 19604361 | 19609419 | NA |
| *Pmi01g2179.1* | Pmi01 | 19609561 | 19616695 | Calreticulin-3 |
| *Pmi01g2180* | Pmi01 | 19617877 | 19624583 | uncharacterized protein LOC18789039 |
| *Pmi01g2181* | Pmi01 | 19627901 | 19632490 | Protein TRIGALACTOSYLDIACYLGLYCEROL 2, chloroplastic |
| *Pmi01g2182* | Pmi01 | 19646085 | 19650904 | NA |
| *Pmi01g2183* | Pmi01 | 19651799 | 19652397 | NA |
| *Pmi01g2184* | Pmi01 | 19677152 | 19678099 | Metalloendoproteinase 2-MMP |
| *Pmi01g2185* | Pmi01 | 19682846 | 19684907 | 60S ribosomal protein L38 |
| *Pmi01g2186* | Pmi01 | 19686286 | 19687514 | NA |
| *Pmi01g2187* | Pmi01 | 19688764 | 19691044 | Putative BPI/LBP family protein At1g04970 |
| *Pmi01g2188* | Pmi01 | 19691826 | 19692137 | Putative BPI/LBP family protein At3g20270 |
| *Pmi02g0624* | Pmi02 | 6977536 | 6980794 | Protein ETHYLENE INSENSITIVE 3 |
| *Pmi02g0625* | Pmi02 | 6985623 | 6988616 | Calcium and calcium/calmodulin-dependent serine/threonine-protein kinase DMI-3 |
| *Pmi02g0626* | Pmi02 | 6989641 | 6994482 | NA |
| *Pmi02g0627* | Pmi02 | 7001166 | 7006343 | Histone-lysine N-methyltransferase SUVR4 |
| *Pmi02g0628* | Pmi02 | 7007145 | 7008115 | NA |
| *Pmi02g0629* | Pmi02 | 7023072 | 7028847 | TMV resistance protein N |
| *Pmi02g2006* | Pmi02 | 22251078 | 22253252 | Reticulon-like protein B5 |
| *Pmi02g2007* | Pmi02 | 22253915 | 22262041 | Golgin candidate 4 |
| *Pmi02g2008* | Pmi02 | 22264443 | 22267077 | U11/U12 small nuclear ribonucleoprotein 59 kDa protein |
| *Pmi02g2009* | Pmi02 | 22268993 | 22271586 | Delta(8)-fatty-acid desaturase |
| *Pmi02g2010* | Pmi02 | 22273922 | 22275581 | Acidic endochitinase |
| *Pmi02g2011* | Pmi02 | 22276503 | 22278511 | Delta(8)-fatty-acid desaturase 1 |
| *Pmi02g2012* | Pmi02 | 22282504 | 22284885 | uncharacterized protein LOC18786326 |
| *Pmi02g2013* | Pmi02 | 22287780 | 22291173 | Protein ABIL1 |
| *Pmi02g2014* | Pmi02 | 22291249 | 22294335 | UDP-D-xylose:L-fucose alpha-1,3-D-xylosyltransferase MGP4 |
| *Pmi02g2015* | Pmi02 | 22295729 | 22304196 | uncharacterized protein LOC18786949 isoform X1 |
| *Pmi02g3012* | Pmi02 | 28549644 | 28555768 | Pentatricopeptide repeat-containing protein At4g38010 |
| *Pmi02g3013* | Pmi02 | 28552934 | 28554853 | Molybdenum cofactor sulfurase |
| *Pmi02g3014* | Pmi02 | 28560233 | 28562837 | Protein HOTHEAD |
| *Pmi02g3015* | Pmi02 | 28567001 | 28567826 | NA |
| *Pmi02g3016* | Pmi02 | 28569822 | 28574783 | Pentatricopeptide repeat-containing protein At2g29760, chloroplastic |
| *Pmi02g3017* | Pmi02 | 28576958 | 28579024 | Sorbitol dehydrogenase |
| *Pmi02g3018* | Pmi02 | 28580531 | 28584711 | Zinc finger CCCH domain-containing protein 48 |
| *Pmi02g3019* | Pmi02 | 28586538 | 28590645 | Calcium-dependent protein kinase 17 |
| *Pmi02g3020* | Pmi02 | 28590738 | 28590966 | NA |
| *Pmi02g3021* | Pmi02 | 28591125 | 28592350 | uncharacterized protein LOC18787055 |
| *Pmi02g3022* | Pmi02 | 28591125 | 28598298 | ABC transporter B family member 28 |
| *Pmi02g3023* | Pmi02 | 28598499 | 28601435 | Post-GPI attachment to proteins factor 3 |
| *Pmi02g3024* | Pmi02 | 28603535 | 28611294 | Valine--tRNA ligase, mitochondrial 1 |
| *Pmi02g3025* | Pmi02 | 28611673 | 28612359 | Dehydration-responsive element-binding protein 1D |
| *Pmi02g3026* | Pmi02 | 28619036 | 28620262 | Ethylene-responsive transcription factor ERF025 |
| *Pmi02g3027* | Pmi02 | 28620694 | 28623070 | TraB domain-containing protein |
| *Pmi02g3028.1* | Pmi02 | 28624862 | 28635853 | Serine/arginine-rich splicing factor RS40 |
| *Pmi02g3029* | Pmi02 | 28640132 | 28642244 | Protein DETOXIFICATION 49 |
| *Pmi02g3030* | Pmi02 | 28654494 | 28657223 | BAG family molecular chaperone regulator 1 |
| *Pmi02g3031* | Pmi02 | 28661069 | 28663316 | Dihydrodipicolinate reductase-like protein CRR1, chloroplastic |
| *Pmi02g3032* | Pmi02 | 28664505 | 28670674 | Probable transcriptional regulator SLK3 |
| *Pmi02g3033* | Pmi02 | 28671195 | 28672326 | F-box protein PP2-A13 |
| *Pmi02g3034* | Pmi02 | 28673879 | 28674085 | NA |
| *Pmi02g3035.1* | Pmi02 | 28675036 | 28676185 | E3 ubiquitin ligase BIG BROTHER-related |
| *Pmi02g3036* | Pmi02 | 28685626 | 28686747 | F-box protein SKIP23 |
| *Pmi02g3037* | Pmi02 | 28688252 | 28691264 | Probable methyltransferase PMT16 |
| *Pmi02g3038* | Pmi02 | 28691666 | 28692073 | Protein MEN-8 |
| *Pmi02g3039* | Pmi02 | 28693269 | 28693899 | Putative F-box protein At5g55150 |
| *Pmi02g3040* | Pmi02 | 28696258 | 28700268 | Protein FAR1-RELATED SEQUENCE 5 |
| *Pmi02g3041* | Pmi02 | 28701089 | 28704245 | Protein IQ-DOMAIN 14 |
| *Pmi02g3042* | Pmi02 | 28705381 | 28705762 | Putative non-specific lipid-transfer protein 14 |
| *Pmi02g3043* | Pmi02 | 28706090 | 28709226 | RHOMBOID-like protein 2 |
| *Pmi02g3044* | Pmi02 | 28715341 | 28719913 | Homeobox-leucine zipper protein ANTHOCYANINLESS 2 |
| *Pmi02g3045* | Pmi02 | 28720919 | 28722265 | Protein ROOT PRIMORDIUM DEFECTIVE 1 |
| *Pmi02g3046* | Pmi02 | 28727698 | 28730043 | S-type anion channel SLAH3 |
| *Pmi02g3047* | Pmi02 | 28733374 | 28737413 | E3 ubiquitin-protein ligase XBAT33 |
| *Pmi02g3048* | Pmi02 | 28738314 | 28739320 | CEN-like protein 1 |
| *Pmi02g3049* | Pmi02 | 28745227 | 28749344 | Leucine-rich repeat receptor protein kinase EMS1 |
| *Pmi03g0772* | Pmi03 | 5848036 | 5865734 | Small subunit processome component 20 homolog |
| *Pmi03g0773* | Pmi03 | 5875562 | 5878455 | Molybdate transporter 2 |
| *Pmi03g0774* | Pmi03 | 5885257 | 5888788 | NA |
| *Pmi03g0775* | Pmi03 | 5889222 | 5892507 | Pentatricopeptide repeat-containing protein At1g15510, chloroplastic |
| *Pmi03g0776* | Pmi03 | 5892845 | 5895435 | Coleoptile phototropism protein 1 |
| *Pmi03g0777* | Pmi03 | 5897901 | 5901882 | Plastidic ATP/ADP-transporter |
| *Pmi03g0778* | Pmi03 | 5915747 | 5915953 | NA |
| *Pmi03g0779* | Pmi03 | 5917515 | 5924413 | Uncharacterized hydrolase YugF |
| *Pmi03g0780* | Pmi03 | 5929420 | 5930948 | NA |
| *Pmi03g0781* | Pmi03 | 5936299 | 5943415 | Protein ANTI-SILENCING 1 |
| *Pmi03g0782* | Pmi03 | 5948712 | 5948894 | NA |
| *Pmi03g0783* | Pmi03 | 5951862 | 5957051 | Boron transporter 4 |
| *Pmi03g0784* | Pmi03 | 5957588 | 5961088 | Translocase of chloroplast 34 |
| *Pmi03g0785* | Pmi03 | 5977381 | 5984958 | Chaperone protein dnaJ A6, chloroplastic |
| *Pmi03g0786* | Pmi03 | 6002074 | 6002433 | NA |
| *Pmi03g0787* | Pmi03 | 6005807 | 6009465 | NA |
| *Pmi03g0788* | Pmi03 | 6010760 | 6015633 | (6-4)DNA photolyase |
| *Pmi03g0789* | Pmi03 | 6016256 | 6020205 | Cytochrome c oxidase subunit 5b-2, mitochondrial |
| *Pmi03g0790* | Pmi03 | 6026803 | 6032054 | Acyl-protein thioesterase 2 |
| *Pmi03g0791* | Pmi03 | 6033167 | 6036538 | CRC domain-containing protein TSO1 |
| *Pmi03g0792* | Pmi03 | 6036557 | 6039089 | CRC domain-containing protein TSO1 |
| *Pmi03g0793* | Pmi03 | 6040450 | 6047112 | Periodic tryptophan protein 2 |
| *Pmi03g0794* | Pmi03 | 6047449 | 6049946 | Monothiol glutaredoxin-S15, mitochondrial |
| *Pmi03g0795* | Pmi03 | 6050441 | 6066749 | NA |
| *Pmi03g0796* | Pmi03 | 6082240 | 6084584 | Probable splicing factor 3A subunit 1 |
| *Pmi03g0797* | Pmi03 | 6086760 | 6090907 | Bifunctional TH2 protein, mitochondrial |
| *Pmi03g0798* | Pmi03 | 6088908 | 6089447 | Polyprotein P3 |
| *Pmi03g0799* | Pmi03 | 6098258 | 6099450 | Late embryogenesis abundant protein 2 |
| *Pmi03g0800* | Pmi03 | 6100645 | 6101981 | Late embryogenesis abundant protein 7 |
| *Pmi03g0801* | Pmi03 | 6103512 | 6111631 | Lys-63-specific deubiquitinase BRCC36 |
| *Pmi03g0802* | Pmi03 | 6130272 | 6132206 | Zinc finger Ran-binding domain-containing protein 2 |
| *Pmi03g0803* | Pmi03 | 6137296 | 6141998 | Biotin carboxyl carrier protein of acetyl-CoA carboxylase |
| *Pmi03g0804* | Pmi03 | 6145377 | 6149390 | Zinc finger CCCH domain-containing protein 39 |
| *Pmi03g0805* | Pmi03 | 6163676 | 6166716 | Probable disease resistance protein At5g63020 |
| *Pmi03g0806* | Pmi03 | 6176858 | 6177331 | uncharacterized protein LOC110761877 |
| *Pmi03g0807* | Pmi03 | 6195914 | 6197490 | 50S ribosomal protein 6, chloroplastic |
| *Pmi03g0808* | Pmi03 | 6199384 | 6201116 | Aspartate racemase |
| *Pmi03g0809* | Pmi03 | 6201979 | 6203345 | Chaperone protein DnaJ 2 |
| *Pmi03g0810* | Pmi03 | 6203997 | 6206028 | uncharacterized protein LOC18783010 |
| *Pmi03g0811* | Pmi03 | 6208693 | 6210521 | F-box only protein 6 |
| *Pmi03g0812* | Pmi03 | 6216948 | 6217165 | Protein DELAY OF GERMINATION 1 |
| *Pmi03g0813* | Pmi03 | 6225435 | 6230227 | Apoptotic protease-activating factor 1 |
| *Pmi03g0814* | Pmi03 | 6230576 | 6233783 | DNA replication complex GINS protein PSF1 |
| *Pmi03g0815* | Pmi03 | 6236701 | 6237060 | MAPK kinase substrate protein At1g80180 |
| *Pmi03g0816* | Pmi03 | 6241593 | 6242507 | Putative pentatricopeptide repeat-containing protein At3g11460, mitochondrial |
| *Pmi03g0817* | Pmi03 | 6242522 | 6243100 | Pentatricopeptide repeat-containing protein At1g59720, chloroplastic/mitochondrial |
| *Pmi03g0818* | Pmi03 | 6244934 | 6248200 | Probable polygalacturonase At1g80170 |
| *Pmi03g0819* | Pmi03 | 6250176 | 6252933 | Peptide deformylase 1A, chloroplastic/mitochondrial |
| *Pmi03g0820* | Pmi03 | 6255462 | 6256144 | uncharacterized protein LOC18784010 |
| *Pmi03g0821* | Pmi03 | 6266930 | 6268945 | Metallothiol transferase FosB |
| *Pmi03g0822* | Pmi03 | 6276357 | 6279358 | Uncharacterized protein At2g34160 |
| *Pmi03g0823* | Pmi03 | 6279741 | 6280094 | Putative ribonuclease H protein At1g65750 |
| *Pmi03g0824* | Pmi03 | 6282021 | 6284946 | Pentatricopeptide repeat-containing protein At1g52640, mitochondrial |
| *Pmi03g0825* | Pmi03 | 6298967 | 6303440 | O-fucosyltransferase 13 |
| *Pmi07g2338* | Pmi07 | 20905005 | 20906742 | uncharacterized protein LOC18771582 |
| *Pmi07g2339* | Pmi07 | 20907420 | 20910291 | BTB/POZ domain and ankyrin repeat-containing protein NPR1 |
| *Pmi07g2340* | Pmi07 | 20911396 | 20913254 | Heat stress transcription factor A-2 |
| *Pmi07g2341* | Pmi07 | 20915201 | 20917932 | 5'-nucleotidase SurE |
| *Pmi07g2342.2* | Pmi07 | 20918520 | 20924302 | Zinc finger CCCH domain-containing protein 65 |
| *Pmi07g2343* | Pmi07 | 20924509 | 20926521 | 30S ribosomal protein S16-2, chloroplastic/mitochondrial |
| *Pmi07g2344.1* | Pmi07 | 20927859 | 20931243 | Nucleosome assembly protein 1;2 |
| *Pmi07g2345* | Pmi07 | 20931905 | 20935512 | DNA repair protein recA homolog 3, mitochondrial |
| *Pmi07g2346* | Pmi07 | 20936525 | 20940021 | Putative transcription factor bHLH041 |
| *Pmi07g2347* | Pmi07 | 20943311 | 20951263 | Meiosis regulator and mRNA stability factor 1 |
| *Pmi07g2348* | Pmi07 | 20952093 | 20953370 | UPF0481 protein At3g47200 |
| *Pmi07g2349* | Pmi07 | 20955174 | 20956446 | UPF0481 protein At3g47200 |
| *Pmi07g2350* | Pmi07 | 20958088 | 20959133 | UPF0481 protein At3g47200 |
| *Pmi07g2351* | Pmi07 | 20960166 | 20961485 | UPF0481 protein At3g47200 |
| *Pmi07g2352* | Pmi07 | 20962602 | 20963236 | Putative LRR receptor-like serine/threonine-protein kinase |
| *Pmi07g2353* | Pmi07 | 20965251 | 20966569 | UPF0481 protein At3g47200 |
| *Pmi07g2354* | Pmi07 | 20968063 | 20970404 | Putative uncharacterized protein YDL057W |
| *Pmi07g2355* | Pmi07 | 20971296 | 20975012 | CRM-domain containing factor CFM3, chloroplastic/mitochondrial |
| *Pmi07g2356* | Pmi07 | 20977061 | 20977282 | NA |
| *Pmi07g2357* | Pmi07 | 20977315 | 20980426 | Cytokinin dehydrogenase 3 |
| *Pmi07g2358* | Pmi07 | 20986849 | 20989368 | Cytokinin dehydrogenase 4 |
| *Pmi07g2359* | Pmi07 | 20991034 | 20993498 | Translation machinery-associated protein 22 |
| *Pmi07g2360* | Pmi07 | 20994901 | 20995350 | LOB domain-containing protein 22 |
| *Pmi07g2390* | Pmi07 | 21199117 | 21200902 | Transcription factor MYBS3 |
| *Pmi07g2391* | Pmi07 | 21204567 | 21207513 | Probable acyl-[acyl-carrier-protein]--UDP-N-acetylglucosamine O-acyltransferase, mitochondrial |
| *Pmi07g2392* | Pmi07 | 21209147 | 21213931 | Inactive protein kinase SELMODRAFT_444075 |
| *Pmi07g2393* | Pmi07 | 21216175 | 21219651 | Protein EFFECTOR OF TRANSCRIPTION 2 |
| *Pmi07g2394* | Pmi07 | 21219727 | 21221346 | Serine acetyltransferase 5 |
| *Pmi07g2395* | Pmi07 | 21221742 | 21223561 | Pentatricopeptide repeat-containing protein At2g15690, mitochondrial |
| *Pmi07g2396* | Pmi07 | 21221833 | 21222048 | NA |
| *Pmi07g2397* | Pmi07 | 21223886 | 21226450 | Mitochondrial carrier protein CoAc2 |
| *Pmi07g2398* | Pmi07 | 21227081 | 21229869 | Thiamine phosphate phosphatase-like protein |
| *Pmi07g2399* | Pmi07 | 21231142 | 21235334 | Protein NDL1 |
| *Pmi07g2400* | Pmi07 | 21235771 | 21238899 | Pentatricopeptide repeat-containing protein At4g13650 |
| *Pmi07g2401* | Pmi07 | 21244007 | 21249052 | Pentatricopeptide repeat-containing protein At2g27610 |
| *Pmi07g2402* | Pmi07 | 21249084 | 21251924 | NA |
| *Pmi07g2403* | Pmi07 | 21252221 | 21254828 | NA |
| *Pmi07g2404* | Pmi07 | 21255005 | 21257489 | Probable protein arginine N-methyltransferase 1 |
| *Pmi07g2405* | Pmi07 | 21258279 | 21262604 | Xaa-Pro dipeptidase |
| *Pmi07g2406* | Pmi07 | 21266683 | 21268360 | 1-aminocyclopropane-1-carboxylate synthase 7 |
| *Pmi07g2407* | Pmi07 | 21274737 | 21276297 | 1-aminocyclopropane-1-carboxylate synthase 7 |
| *Pmi07g2408* | Pmi07 | 21279499 | 21281827 | uncharacterized protein LOC18769397 |
| *Pmi07g2409* | Pmi07 | 21282744 | 21285193 | Probable phospholipase A2 homolog 1 |
| *Pmi07g2410* | Pmi07 | 21285282 | 21285503 | NA |
| *Pmi07g2411.1* | Pmi07 | 21292796 | 21299070 | IST1-like protein |
| *Pmi07g2412* | Pmi07 | 21299890 | 21301279 | Probable caffeoyl-CoA O-methyltransferase At4g26220 |
| *Pmi07g2413* | Pmi07 | 21301923 | 21303971 | Probable caffeoyl-CoA O-methyltransferase At4g26220 |
| *Pmi07g2414* | Pmi07 | 21304202 | 21306480 | Deoxyhypusine synthase |
| *Pmi07g2415.1* | Pmi07 | 21307326 | 21309141 | 60S ribosomal protein L28-1 |
| *Pmi07g2416* | Pmi07 | 21310287 | 21313545 | Histone acetyltransferase type B catalytic subunit |
| *Pmi07g2417* | Pmi07 | 21313564 | 21315972 | uncharacterized protein LOC18769115 |
| *Pmi07g2418* | Pmi07 | 21316088 | 21323502 | Zinc protease PQQL-like |
| *Pmi07g2419* | Pmi07 | 21324203 | 21326096 | 60S ribosomal protein L31-1 |
| *Pmi07g2420* | Pmi07 | 21326376 | 21327209 | Pre-mRNA-splicing factor ATP-dependent RNA helicase DEAH1 |
| *Pmi07g2421* | Pmi07 | 21327310 | 21328638 | Pre-mRNA-splicing factor ATP-dependent RNA helicase DEAH1 |
| *Pmi07g2422* | Pmi07 | 21328654 | 21328974 | Pre-mRNA-splicing factor ATP-dependent RNA helicase DEAH1 |
| *Pmi07g2423* | Pmi07 | 21329666 | 21333020 | uncharacterized protein LOC18769800 |
| *Pmi07g2424* | Pmi07 | 21335556 | 21338931 | IAA-amino acid hydrolase ILR1-like 4 |
| *Pmi07g2425* | Pmi07 | 21351416 | 21354772 | IAA-amino acid hydrolase ILR1-like 1 |
| *Pmi07g2426* | Pmi07 | 21369910 | 21373268 | Vacuolar protein sorting-associated protein 32 homolog 2 |
| *Pmi07g2427* | Pmi07 | 21374014 | 21374973 | Arabinogalactan protein 20 |
| *Pmi07g2428* | Pmi07 | 21375649 | 21377913 | Meiotic nuclear division protein 1 homolog |
| *Pmi07g2429* | Pmi07 | 21379531 | 21383337 | Probable LRR receptor-like serine/threonine-protein kinase At4g29180 |
| *Pmi07g2430* | Pmi07 | 21386307 | 21387799 | Transcription factor RAX3 |
| *Pmi07g2431* | Pmi07 | 21391321 | 21393571 | Zinc finger CCCH domain-containing protein 20 |
| *Pmi07g2469* | Pmi07 | 21599289 | 21601501 | Senescence/dehydration-associated protein At4g35985, chloroplastic |
| *Pmi07g2470* | Pmi07 | 21601629 | 21605564 | Uncharacterized protein C3F10.06c |
| *Pmi07g2471* | Pmi07 | 21610509 | 21616424 | Putative ABC transporter B family member 8 |
| *Pmi07g2472* | Pmi07 | 21616440 | 21617743 | Pseudo histidine-containing phosphotransfer protein 5 |
| *Pmi07g2473* | Pmi07 | 21618654 | 21622528 | Protein phosphatase 2C 56 |
| *Pmi07g2474* | Pmi07 | 21625811 | 21626206 | ERAD-associated E3 ubiquitin-protein ligase HRD1 |
| *Pmi07g2475* | Pmi07 | 21627750 | 21628136 | ERAD-associated E3 ubiquitin-protein ligase HRD1 |
| *Pmi07g2476* | Pmi07 | 21629451 | 21629657 | NA |
| *Pmi07g2477* | Pmi07 | 21631238 | 21634878 | 2-oxoglutarate-Fe(II) type oxidoreductase hxnY |
| *Pmi07g2478* | Pmi07 | 21635219 | 21640285 | NADP-dependent malic enzyme |
| *Pmi07g2479* | Pmi07 | 21640587 | 21650079 | Probable RNA-dependent RNA polymerase 5 |
| *Pmi07g2480* | Pmi07 | 21654058 | 21668147 | uncharacterized protein LOC109950260 |
| *Pmi07g2481* | Pmi07 | 21669294 | 21676028 | Golgin candidate 1 |
| *Pmi07g2482* | Pmi07 | 21679386 | 21680018 | uncharacterized protein LOC18770571 |
| *Pmi07g2483* | Pmi07 | 21687090 | 21688220 | NA |
| *Pmi07g2484* | Pmi07 | 21688722 | 21689348 | Lachrymatory-factor synthase |
| *Pmi07g2485* | Pmi07 | 21694110 | 21696075 | Transcription termination factor MTERF8, chloroplastic |
| *Pmi07g2486* | Pmi07 | 21697038 | 21704743 | Cell division cycle protein 27 homolog B |
| *Pmi07g2487* | Pmi07 | 21705631 | 21708120 | Protein phosphatase 1 regulatory inhibitor subunit PPP1R7 homolog |
| *Pmi07g2488* | Pmi07 | 21708441 | 21710273 | SNW/SKI-interacting protein |
| *Pmi07g2489* | Pmi07 | 21716942 | 21723296 | Dolichyl-diphosphooligosaccharide--protein glycosyltransferase subunit STT3A |
| *Pmi07g2490* | Pmi07 | 21723457 | 21728807 | Protein unc-13 homolog |
| *Pmi07g2491* | Pmi07 | 21732182 | 21734955 | Pentatricopeptide repeat-containing protein At4g02750 |
| *Pmi07g2492* | Pmi07 | 21745021 | 21746790 | Dehydration-responsive element-binding protein 3 |

Note: ‘NA’ indicated Not Applicable.

**Supplementary Table 23** Selected genes in the biological processes associated with plateau adaptability.

| Biological processes | Gene ID | Function annotated by NR or Swiss-Prot database |
| --- | --- | --- |
| DNA repair | *Pmi01g1876* | Fanconi-associated nuclease 1 homolog |
|  | *Pmi03g0781* | Protein ANTI-SILENCING 1 |
|  | *Pmi03g0788* | (6-4)DNA photolyase |
|  | *Pmi03g0801* | Lys-63-specific deubiquitinase BRCC36 |
|  | *Pmi07g2344* | Nucleosome assembly protein 1;2 |
|  | *Pmi07g2345* | DNA repair protein recA homolog 3 |
|  | *Pmi07g2347* | Meiosis regulator and mRNA stability factor 1 |
|  | *Pmi07g2428* | Meiotic nuclear division protein 1 homolog |
| Induction of programmed cell death | *Pmi02g3030* | BAG family molecular chaperone regulator 1 |
|  | *Pmi02g3045* | Protein ROOT PRIMORDIUM DEFECTIVE 1 |
|  | *Pmi03g0803* | Biotin carboxyl carrier protein of acetyl-CoA carboxylase |
| Response to cold | *Pmi01g2181* | Protein TRIGALACTOSYLDIACYLGLYCEROL 2 |
|  | *Pmi02g2009* | Delta(8)-fatty-acid desaturase |
|  | *Pmi02g2010* | Acidic endochitinase |
|  | *Pmi02g2011* | Delta(8)-fatty-acid desaturase 1 |
|  | *Pmi02g3013* | Molybdenum cofactor sulfurase |
|  | *Pmi02g3025* | Dehydration-responsive element-binding protein 1D |
|  | *Pmi02g3042* | Putative non-specific lipid-transfer protein 14 |
|  | *Pmi03g0799* | Late embryogenesis abundant protein 2 |
|  | *Pmi03g0800* | Late embryogenesis abundant protein 7 |
|  | *Pmi07g2391* | Probable acyl-[acyl-carrier-protein]--UDP-N-acetylglucosamine O-acyltransferase |
|  | *Pmi07g2394* | Serine acetyltransferase 5 |
|  | *Pmi07g2469* | Senescence/dehydration-associated protein At4g35985 |
|  | *Pmi07g2473* | Protein phosphatase 2C 56 |
|  | *Pmi07g2492* | Dehydration-responsive element-binding protein 3 |
| Response to UV | *Pmi02g3031* | Dihydrodipicolinate reductase-like protein CRR1 |
|  | *Pmi02g3040* | Protein FAR1-RELATED SEQUENCE 5 |
|  | *Pmi02g3044* | Homeobox-leucine zipper protein ANTHOCYANINLESS 2 |
|  | *Pmi03g0775* | Pentatricopeptide repeat-containing protein At1g15510 |
|  | *Pmi03g0788* | (6-4)DNA photolyase |

**Supplementary Table 24** The genotype of 35 bp deletion in the promoter of *Prupe. 2G053600* in varieties with different root-knot nematode resistance. The primers can be found in Table S26. The resistance of different varieties was refferred to Zhu et al. (2000).

| Species | Varieties | 35 bp in the promoter of *Prupe.2G053600* | The resistance to RKN |
| --- | --- | --- | --- |
| *P. mira* | 2010-138 | + | Susceptible |
| *P. davidiana* | Hong Hua Shan Tao | + | Resistance |
|  | Bai Hua Shan Tao | + | Resistance |
| *P. kansuensis* | Hong Gen Gan Su Tao | + | Resistance |
|  | Bai Gen Gan Su Tao | + | Resistance |
| *P. persica* | Hong Shu Xing | - | Resistance |
|  | Nemaguard | - | Resistance |
|  | Tsukuba 3# | - | Resistance |
|  | Okinawa | - | Resistance |
|  | Qingzhou Bai Pi Mi Tao | + | Resistance |
|  | Zhong Bi Fen Xiu | - | Resistance |
|  | Harrow Blood | - | Susceptible |
|  | Bailey | - | Susceptible |
|  | Fen Hua Bi Tao | - | Susceptible |
|  | Fen Shou Xing | - | Susceptible |
|  | Siberian C | - | Susceptible |
|  | Shen Zhou Shui Mi | - | Susceptible |
|  | Wu Bao Tao | - | Susceptible |
|  | Hun Chun Tao | - | Susceptible |

**Supplementary Table 25** The genotype of 19 bp deletion in the promoter of *Pmi02g3025* in varieties with different cold resistance.The primers can be found in Table S26. The resistance of different varieties was refferred to unpublished data of our laboratory by using young branches.

| Species | Varieties | 19 bp deletion in the promoter of *Pmi02g3025* | The resistance to low temperature (4 ℃) |
| --- | --- | --- | --- |
| *P. persica* | An Nong Shui Mi | - | 9 |
|  | Zao Yan | - | 9 |
|  | Tai Bai | - | 9 |
|  | Yan Hong | - | 7 |
|  | Yumyeong | - | 7 |
|  | Shenzhou Bai Mi | - | 7 |
|  | Ge Gu | - | 5 |
|  | Okubo | - | 5 |
|  | Zhong Hua Shou Tao | - | 3 |
|  | Okitsu | - | 3 |
|  | Hardired | - | 3 |
|  | Wuhan Da Hong Pao | - | 1 |
|  | Ying Xue | - | 1 |
|  | Xi Mei 2# | - | 1 |

**Supplementary Table 26** The primers used in the study.

| Primer name | Primer sequence (5’-3’) | Application |
| --- | --- | --- |
| Prupe.2G053600-PRI101-F | ttgatacatatgcccgtcgacATGGCTTTGATCGGAGAGGC | Amplification of *Prupe.2G053600* for gene transformation |
| Prupe.2G053600-PRI101-R | cgatcggggaaattcgagctcTTATATGATCACTTCGTTGCCTA |  |
| Prupe.2G053600-35S-DNA-F | GAAACCTCCTCGGATTCCATT | PCR validation of plants with *Prupe.2G053600* transformation |
| Prupe.2G053600-35S-DNA-R | GTCGACGGGCATATGTATCAA |  |
| Prupe.2G053600-35S-RNA-F | AGTTGTGGATGACGCAGAGG | qRT-PCR validation of plants with *Prupe.2G053600* transformation |
| Prupe.2G053600-35S-RNA-R | CACCTTCGAGCCTAAGGCAA |  |
| Prupe.2G053600-35bp-F | CGAATTGTCCAACGCTGTCG | Validation of genotype of 35 bp deletion in the promoter of *Prupe.2G053600* in nature and cross population |
| Prupe.2G053600-35bp-R | CAAATGCTAAGCGCCGTCAA |  |
| Prupe.2G053600-Pro-0-F | gaccatgattacgccaagcttATAAGGGATGGACTAAACATTGGAA | Amplification of promoter sequence (2063 bp) with the reverse primer of Prupe.2G053600-Pro-0-R |
| Prupe.2G053600-Pro-1-F | gaccatgattacgccaagcttCTTCTTGCCTGTCTCAAAGATAAATC | Amplification of promoter sequence (1497 bp) with the reverse primer of Prupe.2G053600-Pro-0-R |
| Prupe.2G053600-Pro-2-F | gaccatgattacgccaagcttTAGTGTCATCTTTGTTTTTGGAAACAG | Amplification of promoter sequence (693 bp) with the reverse primer of Prupe.2G053600-Pro-0-R |
| Prupe.2G053600-Pro-3-F | gaccatgattacgccaagcttGTTGTCTTGCCCCACTTCTGC | Amplification of promoter sequence (282 bp) with the reverse primer of Prupe.2G053600-Pro-0-R |
| Prupe.2G053600-Pro-4-F | gaccatgattacgccaagcttGACCAGTACTTGAAAGAGGGGTT | Amplification of promoter sequence (161 bp) with the reverse primer of Prupe.2G053600-Pro-0-R |
| Prupe.2G053600-Pro-0-R | accacccggggatcctctagaGAGGAGTGGCTCATCGAGTTTC | The reverse primer for amplification of the promoter sequence of *Prupe.2G053600* |
| Pmi02g3025-PRI101-F | catatgcccgtcgaccccgggATGGATGGTTTTTGTCCTTACTACG | Amplification of *Pmi02g3025* for gene transformation |
| Pmi02g3025-PRI101-R | gttgattcagaattcggatccTTAAATCGAATAACTCCATAGTGGCA |  |
| Pmi02g3025-Pro-1-F | AGTTGGGCTAGTTCTTTCAATATACAA | Amplification of the promoter sequence of *Pmi02g3025* |
| Pmi02g3025-Pro-1-R | CGTCATCCTCGGTGTAAAGAGG |  |
| Pmi02g3025-Pro-2-F | TCCACCAGAGCCGTAAACAC |  |
| Pmi02g3025-Pro-2-R | CCGAGCCAAATCCTGGTCTT |  |
| Pmi02g3025-Gene-1-F | ATGGATGGTTTTTGTCCTTACTACG | Amplification of the genic sequence of *Pmi02g3025* |
| Pmi02g3025-Gene-1-R | TTAAATCGAATAACTCCATAGTGGCA |  |
| Pmi02g3025-Gene-2-F | TACCTACCAAAAACAGGAATGATGC |  |
| Pmi02g3025-Gene-2-R | ACGTGCCACTATGGAGTTATTCGAT |  |
| Pmi02g3025-35S-RNA-F | GCTTGCTTGAACTTTGCCGA | qRT-PCR validation of plants with *Pmi02g3025* transformation |
| Pmi02g3025-35S-RNA-R | CAGAAACCTCATCCGACCCC |  |
| Pmi02g3025-19bp-F | GCTTGCTTGAACTTTGCCGA | Validation of genotype of 19 bp deletion in the promoter of *Pmi02g3025* in nature population |
| Pmi02g3025-19bp-R | TGTTCCGATGCTGACTTGCT |  |
