# Supplementary material for: Chromosome-level genome assemblies of four wild peach species provide insights into genome evolution and genetic basis of stress resistance
Source: BMC Biol. 2022 Jun 13;20:139. doi: 10.1186/s12915-022-01342-y (PMC9195245; doi:10.1186/s12915-022-01342-y)
Supplement: Supplementary file 2 — Additional file 2: Fig. S1. Estimation of genome sizes of P. mira (a), P. davidiana (b), P. kansuensis (c), and P. ferganensis (d) based on K-mer analysis. Fig. S2. Genome size, annotated gene number, and the length of tandam repeat sequences, interpersed repeat sequences in P. mira, P. davidiana, P. kansuensis, and P. ferganensis. Fig. S3. Genome variations across the pseudo-chromosomes of four wild peach species compared to the reference (Prunus persica). The circles from the outer to the inner (A-P) represent copy number variation (CNV) density in P. ferganensis (A), P. kansuensis (B), P. davidiana (C), and P. mira (D), and structure variations (SVs) in P. ferganensis (E), P. kansuensis (F), P. davidiana (G), and P. mira (H), and indels in P. ferganensis (I), P. kansuensis (J), P. davidiana (K), and P. mira (L), as well as SNPs in P. ferganensis (M), P. kansuensis (N), P. davidiana (O), and P. mira (P) in each sliding window of 0.1 Mb. Fig. S4. The electrophoresis results of 20 primers which amplified in two varieties (2010-138 and Shen Zhou Li He Shui Mi) belonging to P. mira (Pm) and P. persica (Pp), respectively. Fig. S5. KEGG pathways enriched in genes comprising large-effect SNPs of between P. ferganensis and P. persica. Fig. S6. KEGG pathways enriched in genes comprising indels in four wild peach species compared to P. persica. Fig. S7. KEGG pathways enriched in genes comprising structure variations in P. mira and P. kansuensiss compared to P. persica. Fig. S8. KEGG pathways enriched in genes comprising copy number variations in P. ferganensis (a), P. kansuensis (b), P. davidiana (c), and P. mira (d) compared to P. persica. Fig. S9. Venn diagram of gene families identified from the five species of peach. Fig. S10. Statistics of single-copy orthologs, multiple-copy orthologs, and unique orthologs in 15 species. Fig. S11. KEGG pathways enriched in expanded and contracted gene families of the four wild peach species. Fig. S12. Whole-genome duplication and spec [file 12915_2022_1342_MOESM2_ESM.docx]

**
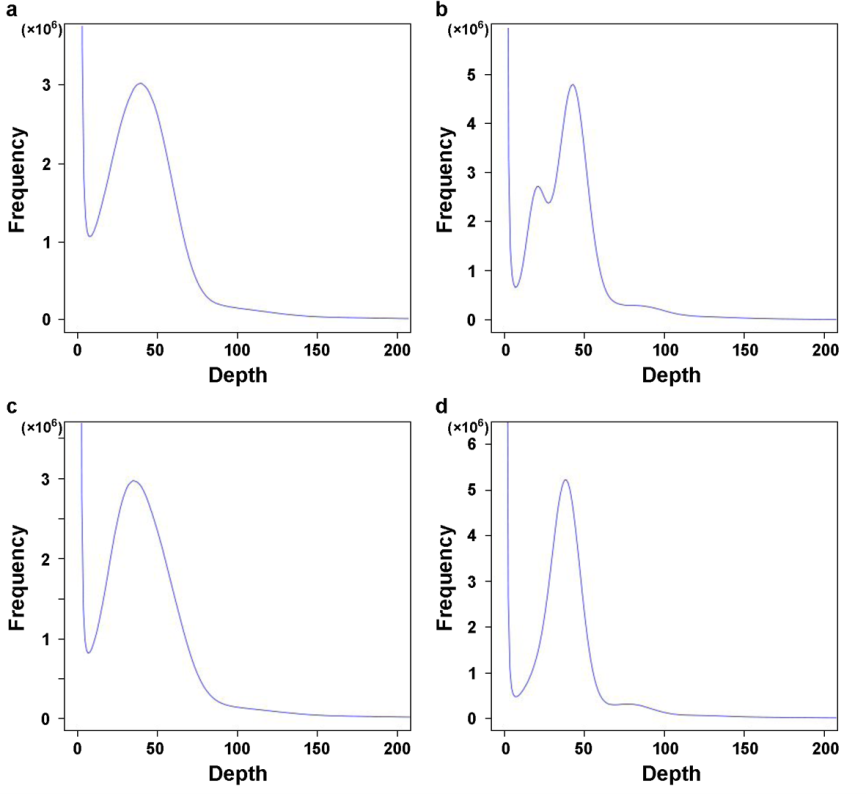
**

**Supplementary Figure 1 Estimation of genome sizes of *P. mira* (a), *P. davidiana* (b), *P. kansuensis* (c), and *P. ferganensis* (d) based on K-mer analysis.**

**
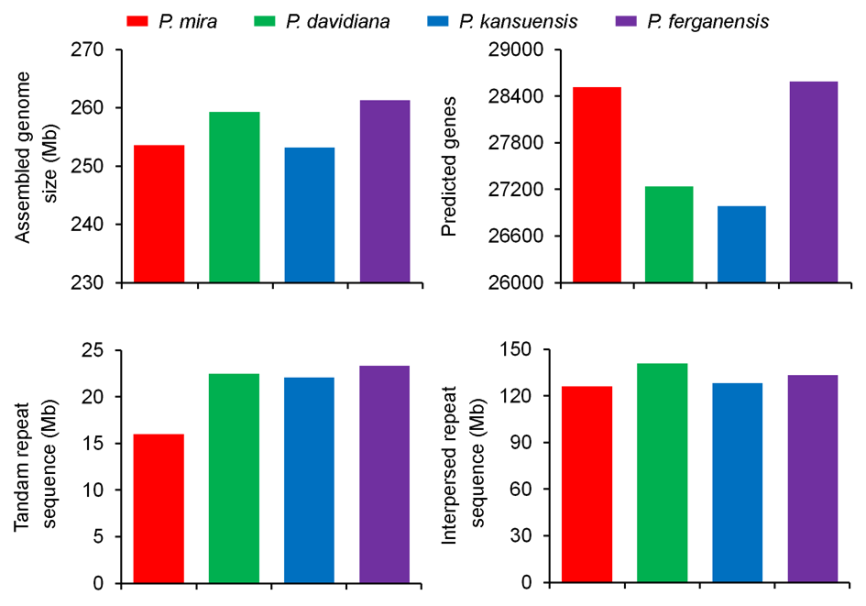
**

**Supplementary Figure 2 Genome size, annotated gene number, and the length of tandam repeat sequences, interpersed repeat sequences in *P. mira*, *P. davidiana*, *P. kansuensis*, and *P. ferganensis.***


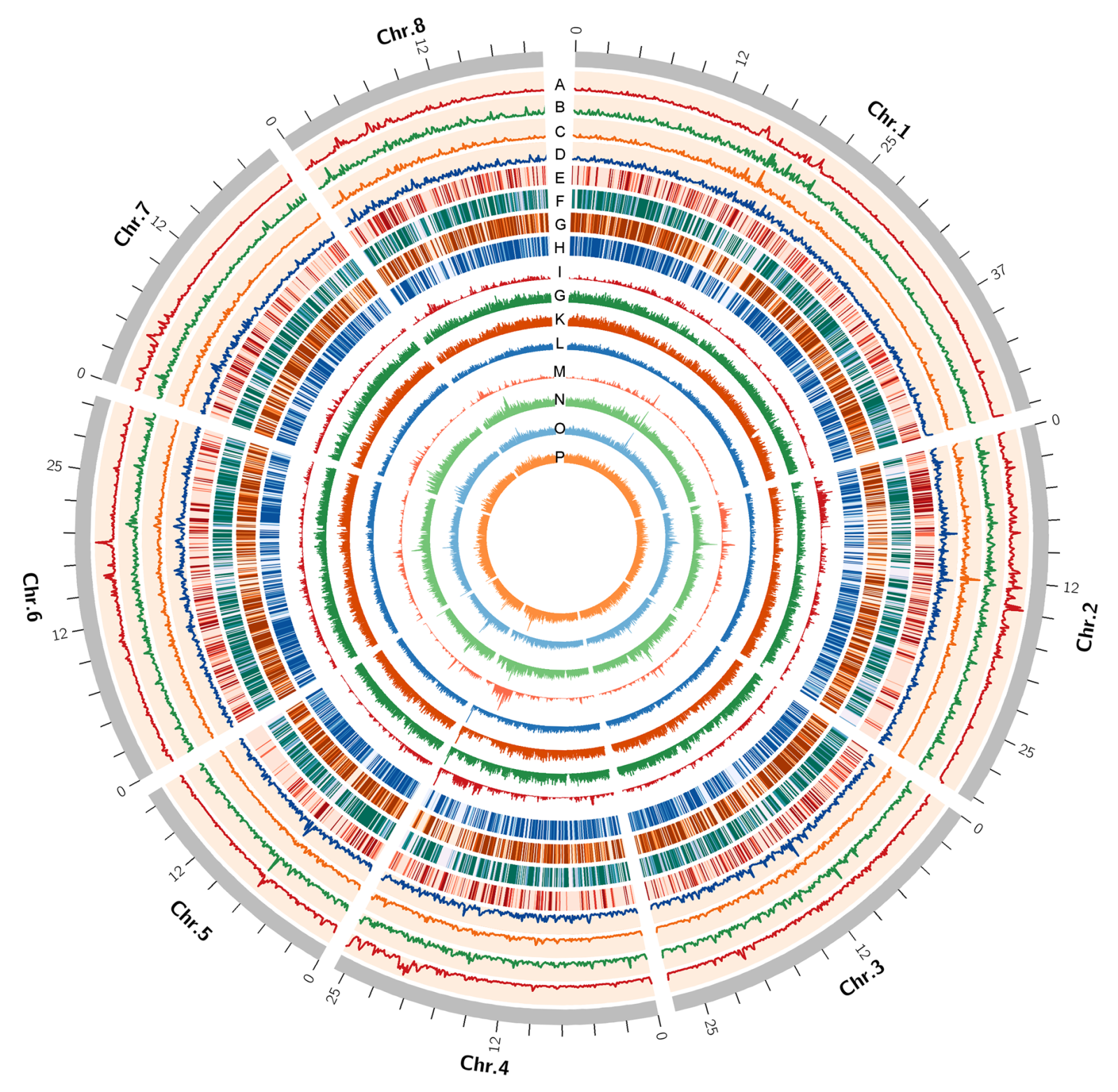


**Supplementary Figure 3 Genome variations across the pseudo-chromosomes of four wild peach species compared to the reference (*Prunus persica*).** The circles from the outer to the inner (A-P) represent copy number variation (CNV) density in *P. ferganensis* (A), *P. kansuensis* (B), *P. davidiana* (C), and *P. mira* (D), and structure variations (SVs) in *P. ferganensis* (E), *P. kansuensis* (F), *P. davidiana* (G), and *P. mira* (H), and indels in *P. ferganensis* (I), *P. kansuensis* (J), *P. davidiana* (K), and *P. mira* (L), as well as SNPs in *P. ferganensis* (M), *P. kansuensis* (N), *P. davidiana* (O), and *P. mira* (P) in each sliding window of 0.1 Mb.


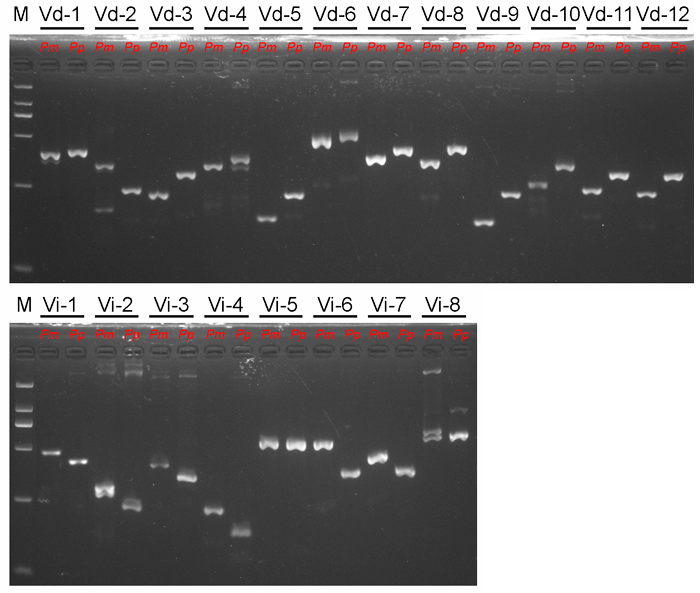


**Supplementary Figure 4 The electrophoresis results of 20 primers which amplified in two varieties (2010-138 and Shen Zhou Li He Shui Mi) belonging to *P. mira* (*Pm*) and *P. persica* (*Pp*), respectively.** The ‘M’ indicate marker, and Vd-1 to Vd-12 and Vi-1 to Vi-8 represents different primers.


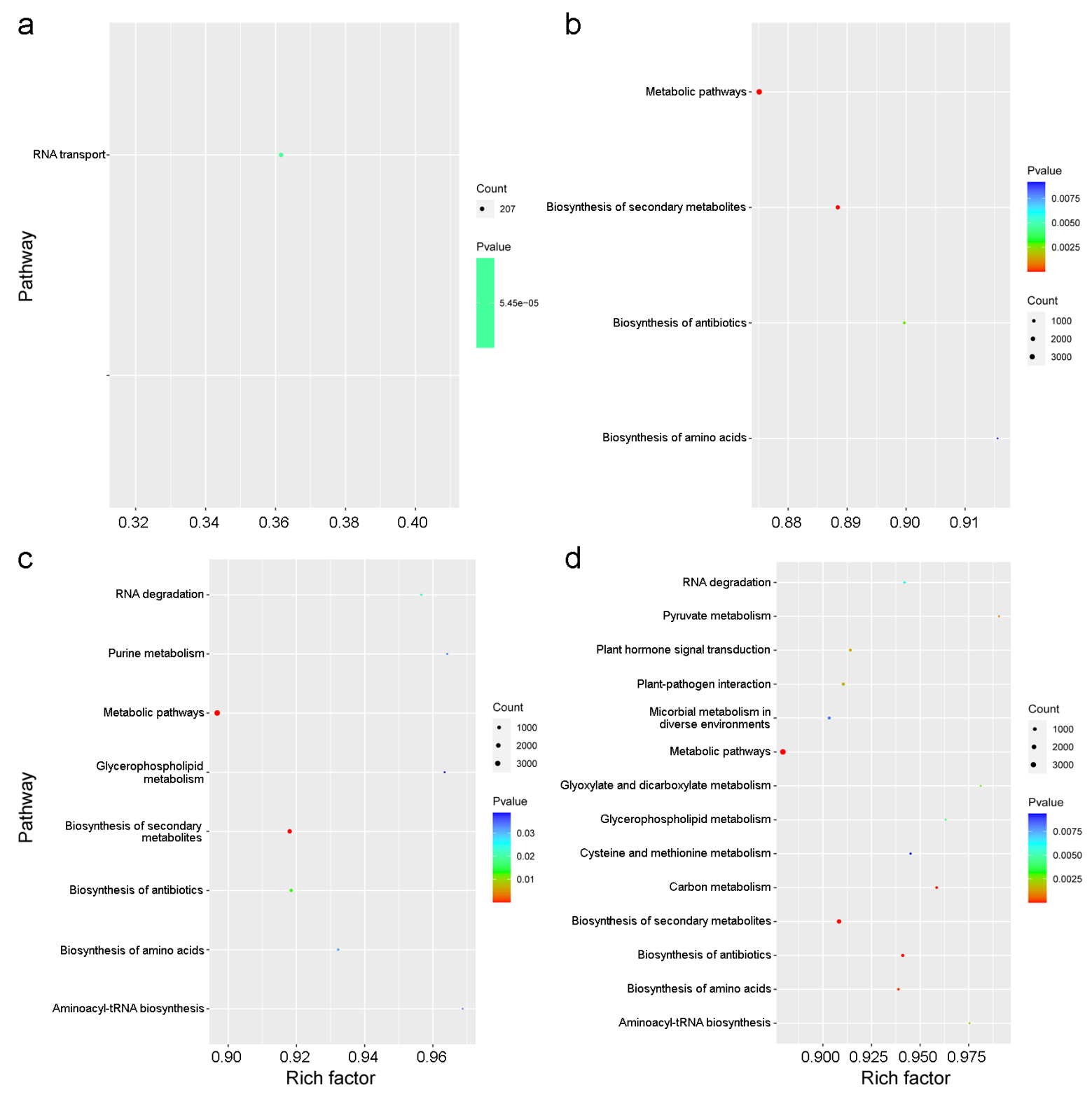


**Supplementary Figure 5 KEGG pathways enriched in genes comprising large-effect SNPs of *P. ferganensis* (a), *P. kansuensis* (b), *P. davidiana* (c), and *P. mira* (d) compared to *P. persica*.**


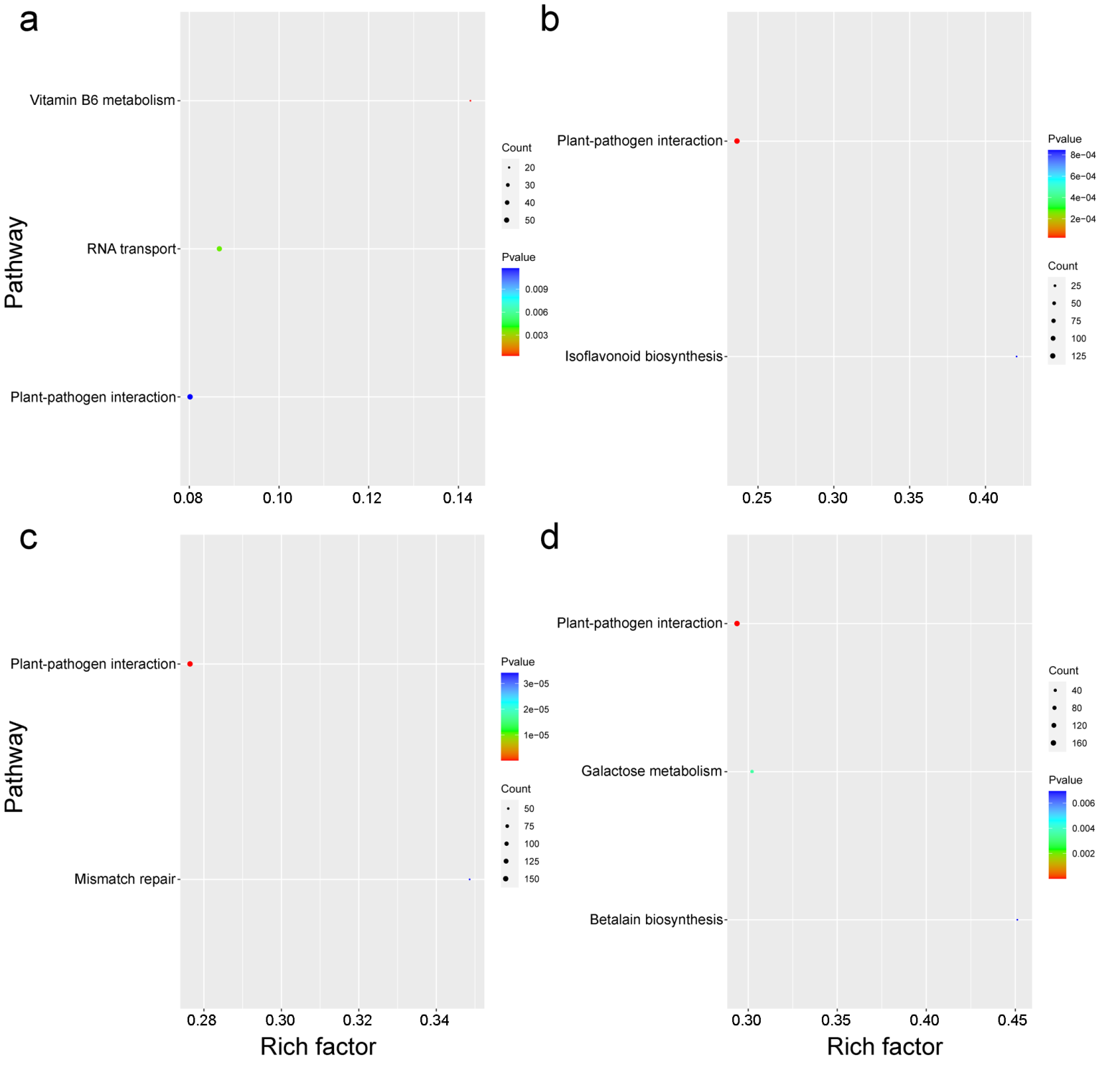


**Supplementary Figure 6 KEGG pathways enriched in genes comprising indels of *P. ferganensis* (a), *P. kansuensis* (b), *P. davidiana* (c), and *P. mira* (d) compared to *P. persica*.**


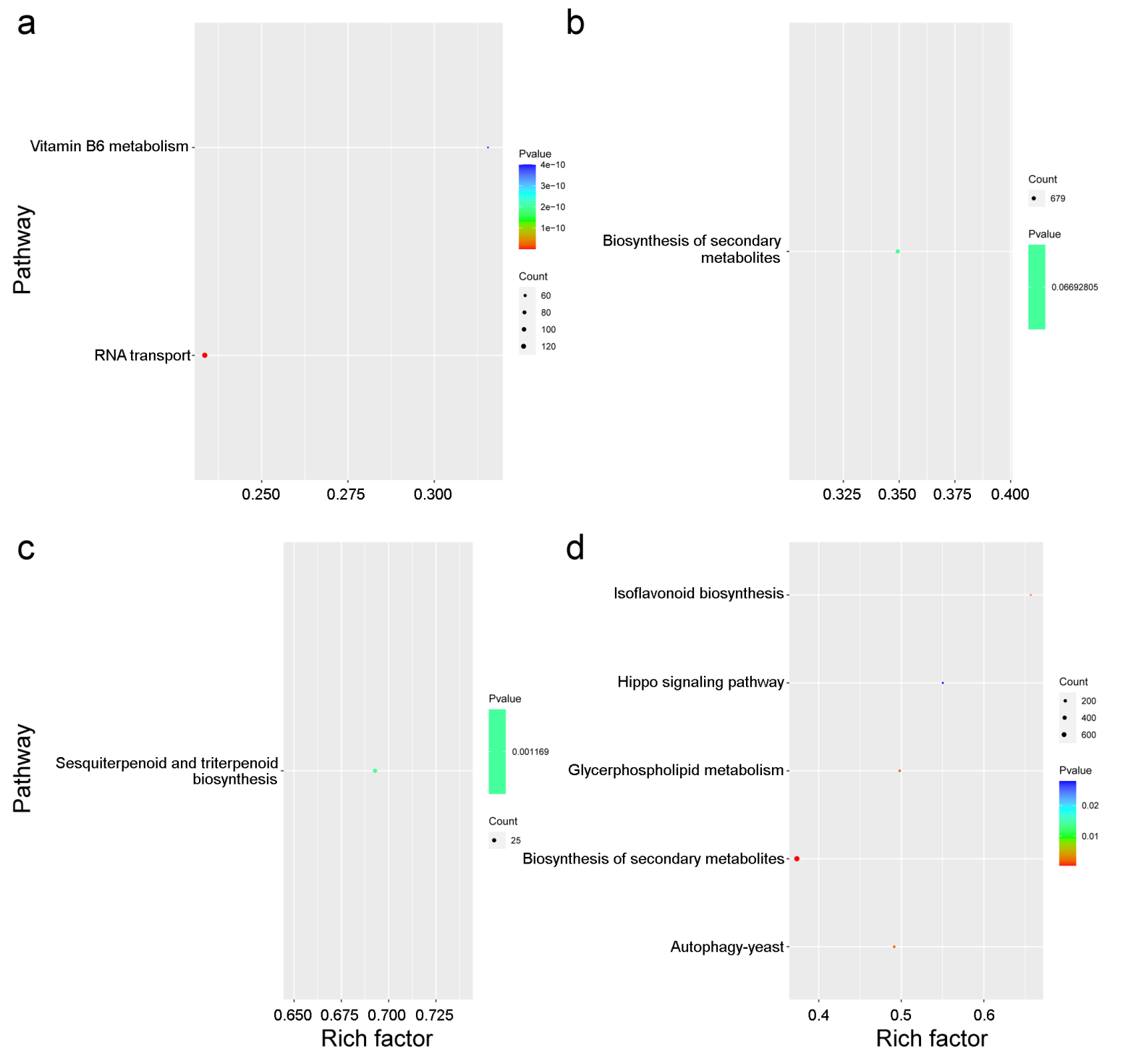


**Supplementary Figure 7 KEGG pathways enriched in genes comprising structure variations of *P. ferganensis* (a), *P. kansuensis* (b), *P. davidiana* (c), and *P. mira* (d) compared to *P. persica*.**

**
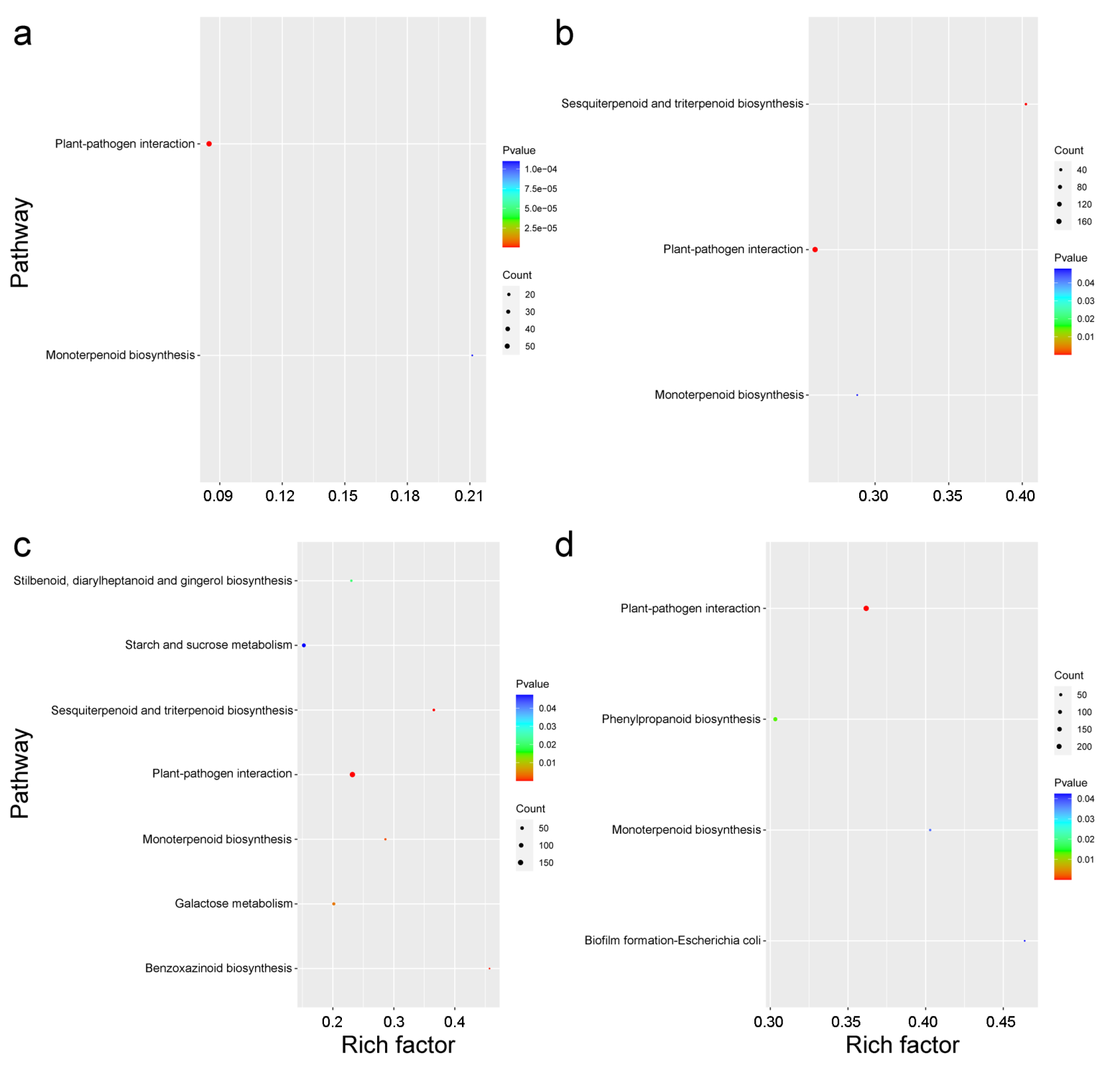
**

**Supplementary Figure 8 KEGG pathways enriched in genes comprising copy number variations in *P. ferganensis* (a), *P. kansuensis* (b), *P. davidiana* (c), and *P. mira* (d) compared to *P. persica*.**


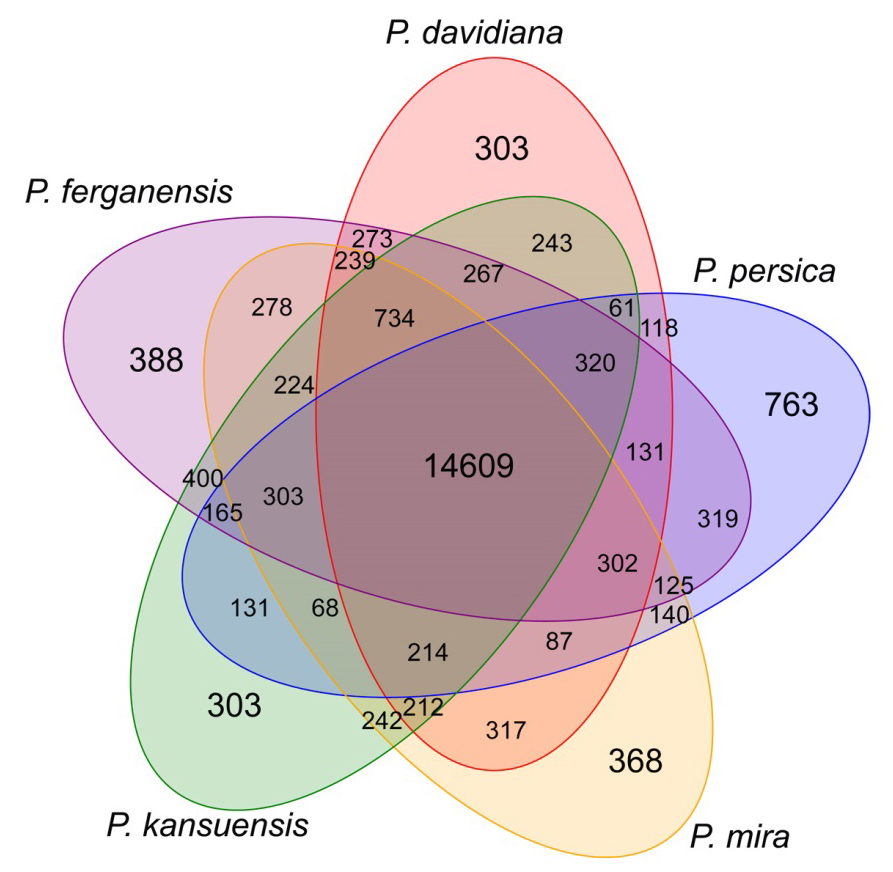


**Supplementary Figure 9 Venn diagram of gene families identified from the five species of peach.**

**
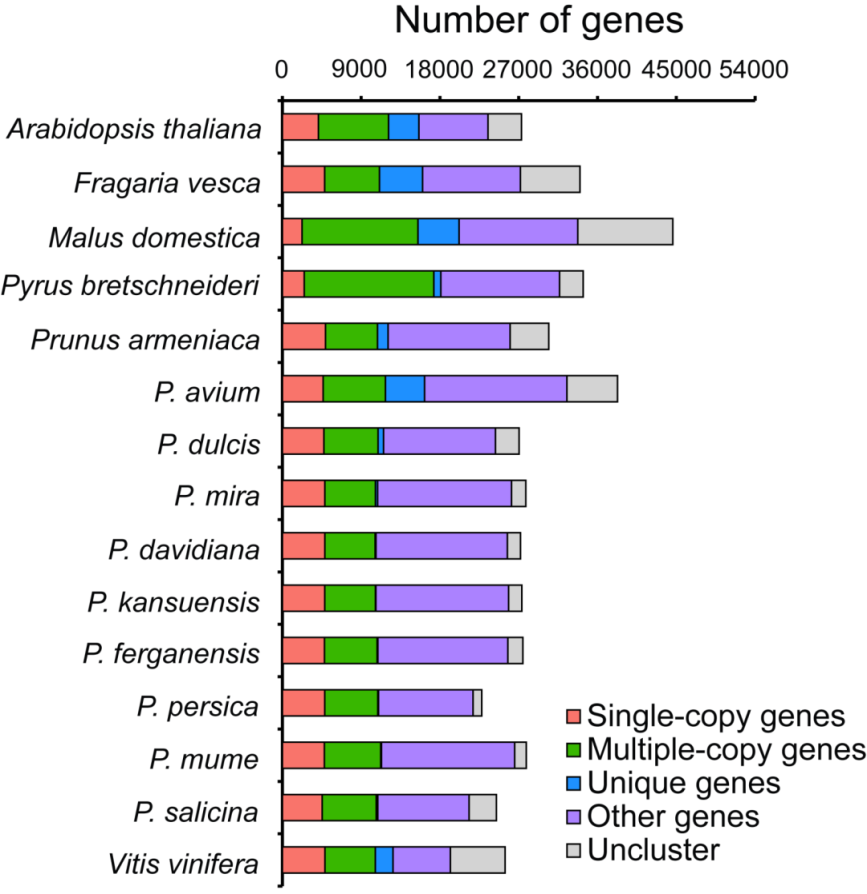
**

**Supplementary Figure 10 Statistics of single-copy orthologs, multiple-copy orthologs, and unique orthologs in 15 species.**

|  |
| --- |
|  |

**Supplementary Figure 11 KEGG pathways enriched in expanded and contracted gene families of the four wild peach species.**

**Supplementary Figure 12 Whole-genome duplication and speciation events in peach as revealed by the distribution of 4DTv distance among paralogous and orthologs genes in different species.**


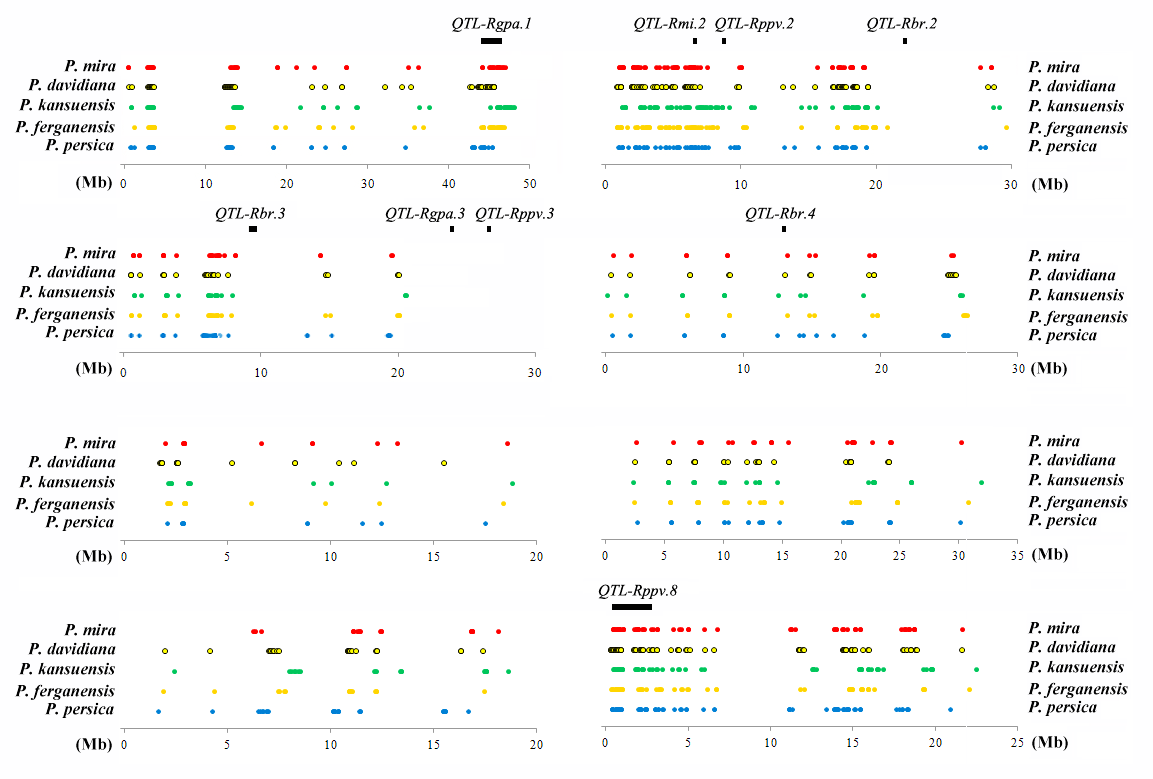


**Supplementary Figure 13 Distribution of resistance (*R*) genes across the 8 chromosomes in five peach species of peach and their overlaps with disease resistance QTLs.** In the figure, ‘*Rm2*’ and ‘*MP.SD-3.1*’ indcated resistance gene or QTLs to green peach aphid which reported by Lambert et al. (2016) and Sauge et al. (2012), respectively, ‘*Rmia*’ indicated resistance to *Meloidogyne incognita* which reported by Duval et al. (2014), ‘*Rppv.2*’ and ‘*Rppv.3*’ indicated resistance QTLs to plum pox virus which reported by Cirilli et al. (2017), ‘*Vr2*’ and ‘*Vr3*’ indicated resistance to peach powdery mildew which reported by Pascal et al. (2017) and Donoso et al. (2016), ‘*SK-if 2009.2*’, ‘*SK-if 2010.2*’, ‘*Fl-rd 2010.3*’, ‘*SK-if 2009.4*’, and ‘*SK-if 2010.4b*’ indicated resistance QTLs to brown rot which reported by Pacheco et al. (2014). And the last number indicated the chromosome number which located that QTL.

**
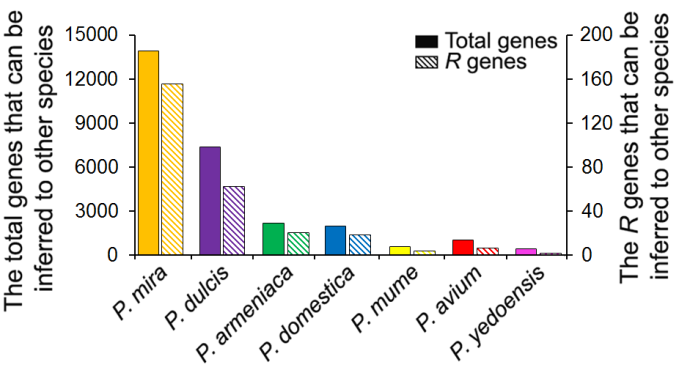
**

**Supplementary Figure 14 Percent of *P. davidiana*-specific contigs covered by reads from different *Prunus* species.**

**
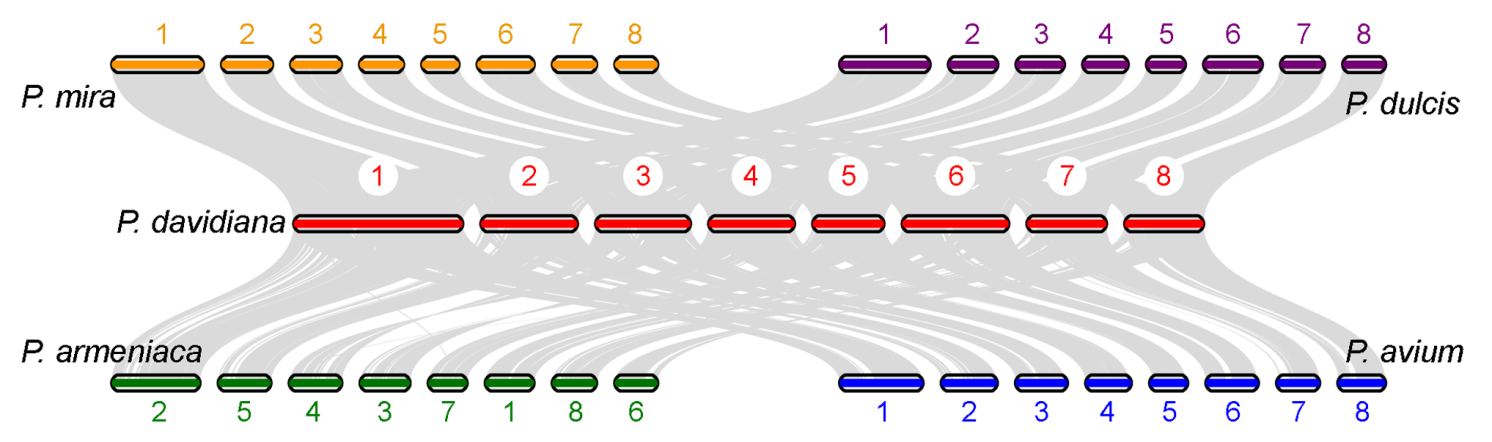
**

**Supplementary Figure 15 Collinearity among *P. davidiana*, *P. mira*, *P. dulcis*, *P. armeniaca*, and *P. avium* genomes.**


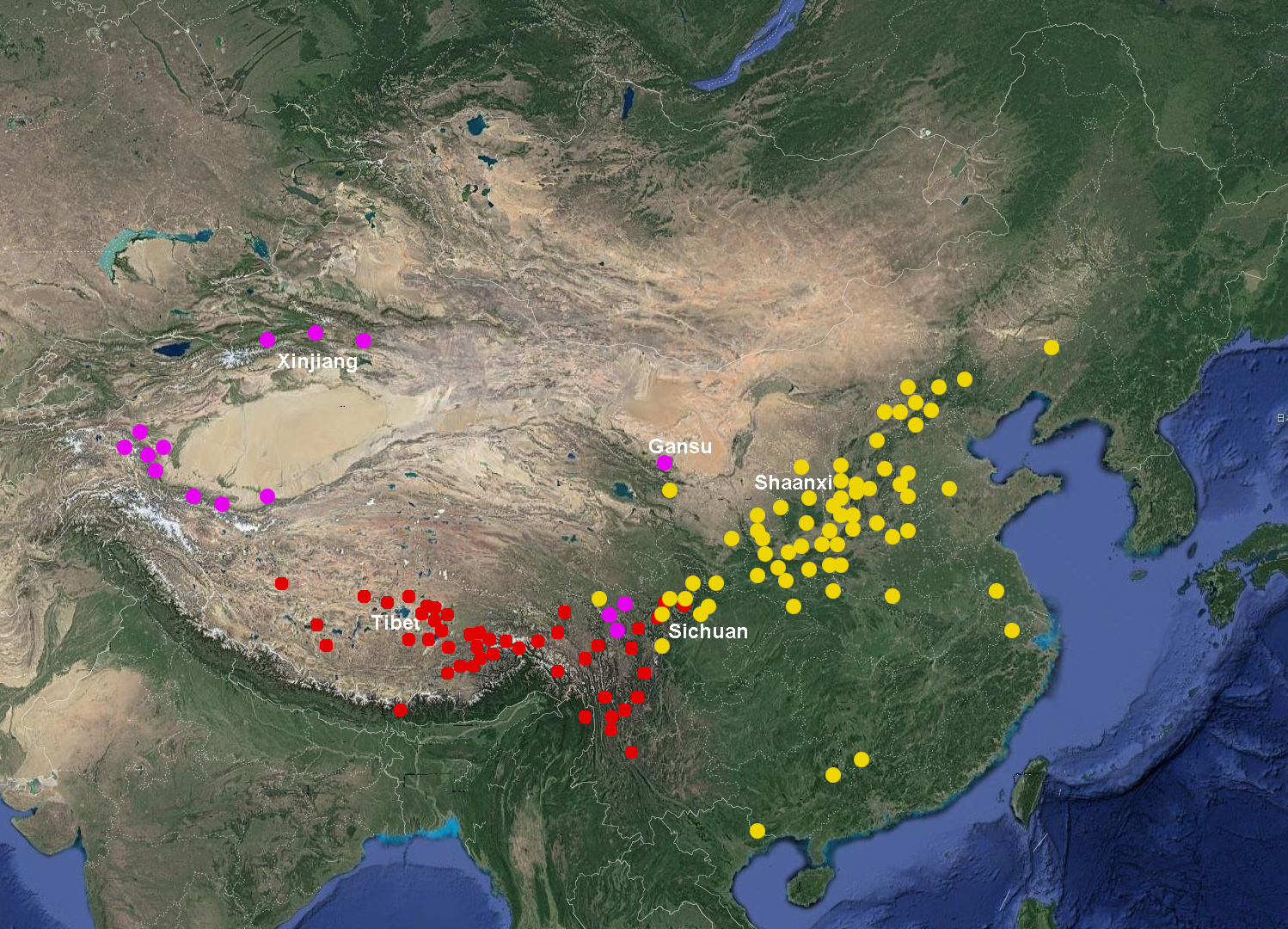


**Supplementary Figure 16 Geographical distribution of *P. mira* (red circle), *P. davidiana* (yellow), and *P. dulcis* (orange) which originated in China.**

**
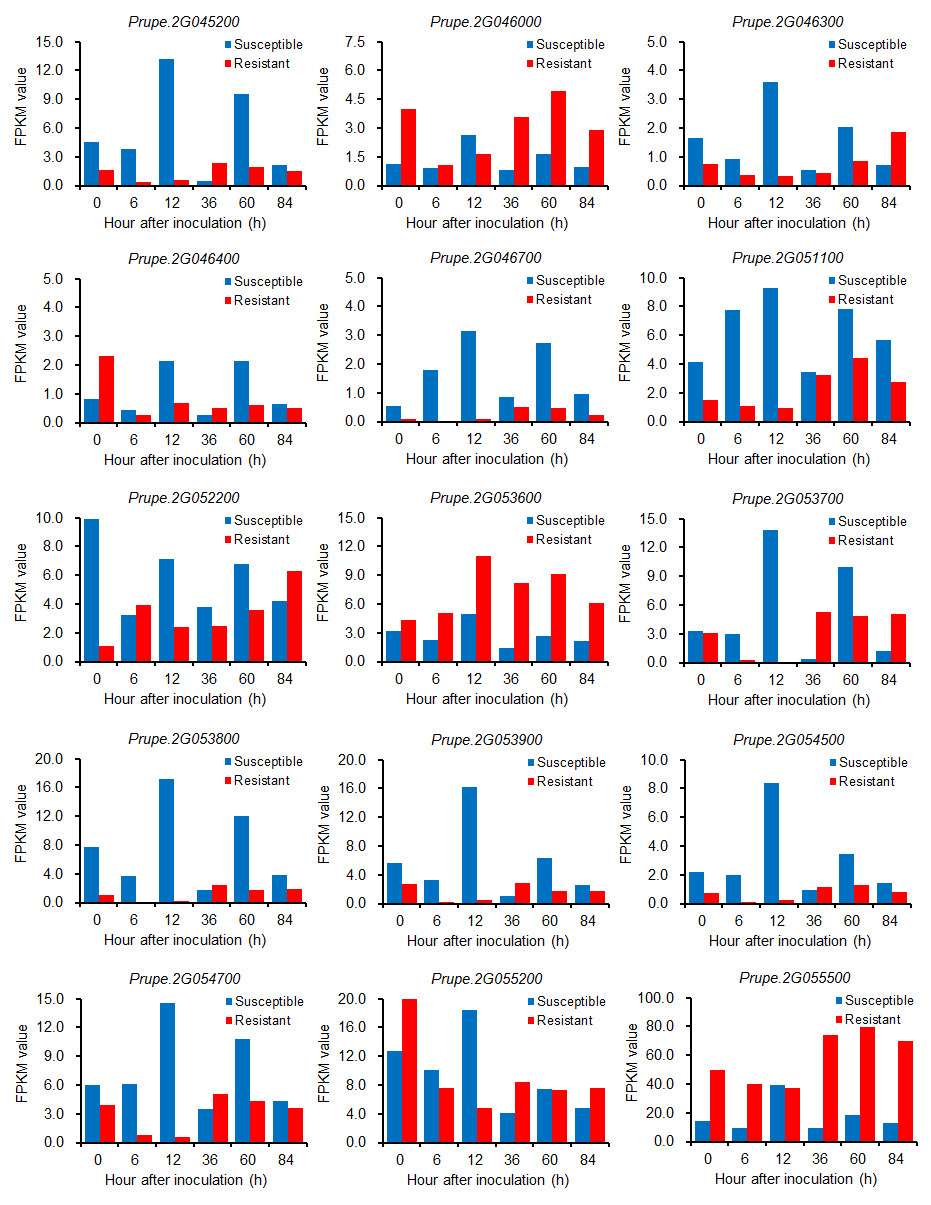
**

**
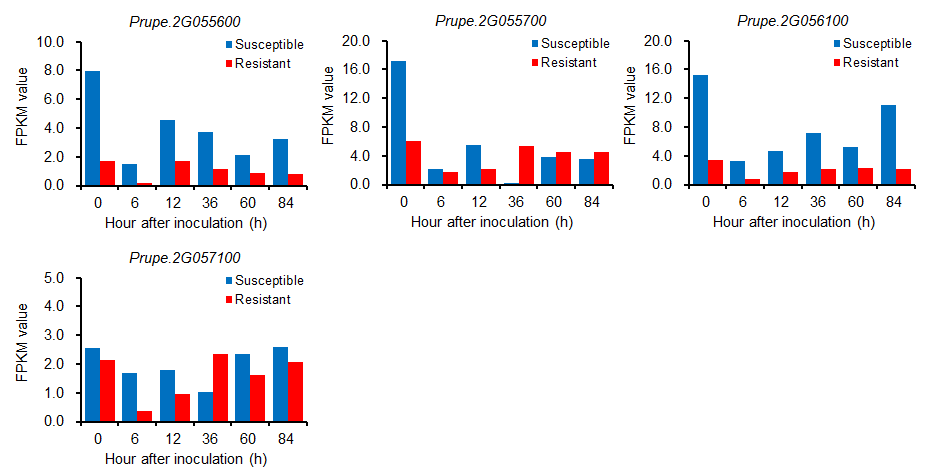
**

**Supplementary Figure 17 Expression of 19 *R* genes in two accessions (‘Hong Gen Gan Su Tao 1#’ and ‘Bailey’) inoculated with nematode after 0, 6, 12, 36, 60, and 84 hours.**

**
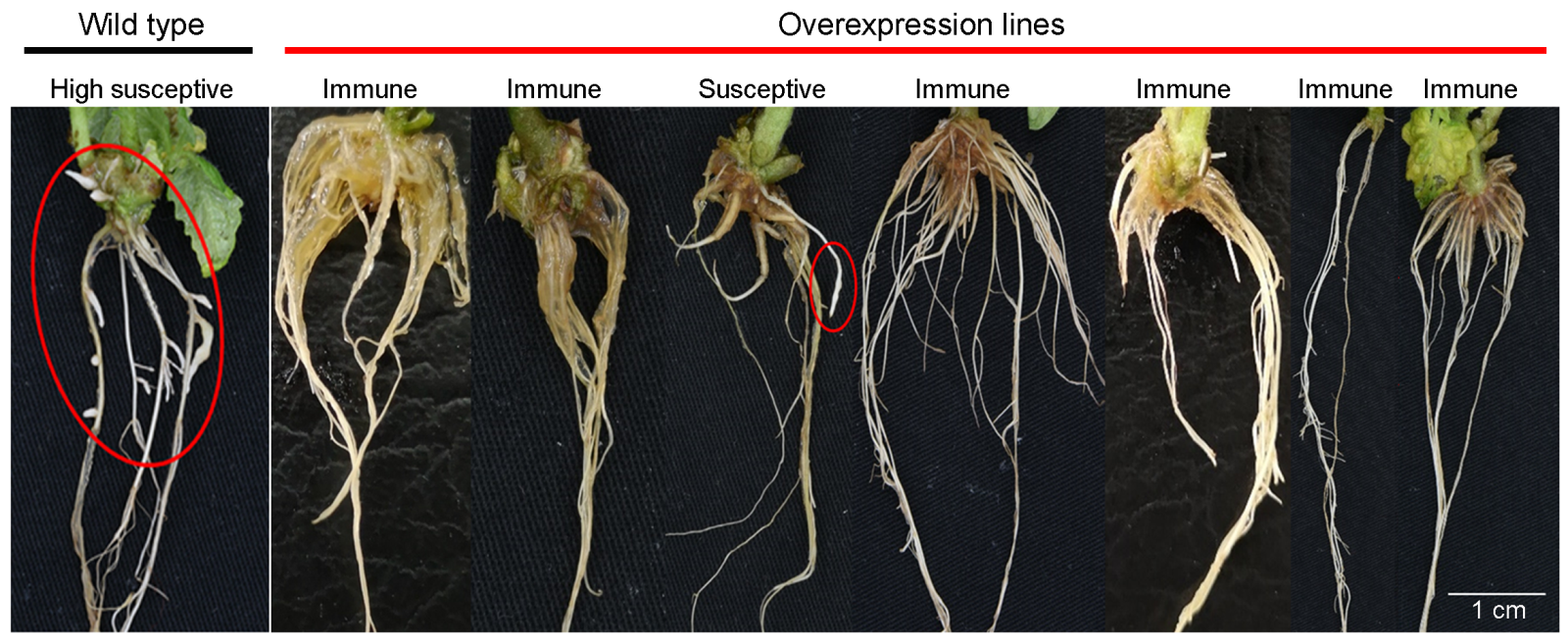
**

**Supplementary Figure 18 The evaluation of resistant to root-knot nematodes in transgenic tomato lines with *Prupe.2G053600* gene and its wild type.**

**
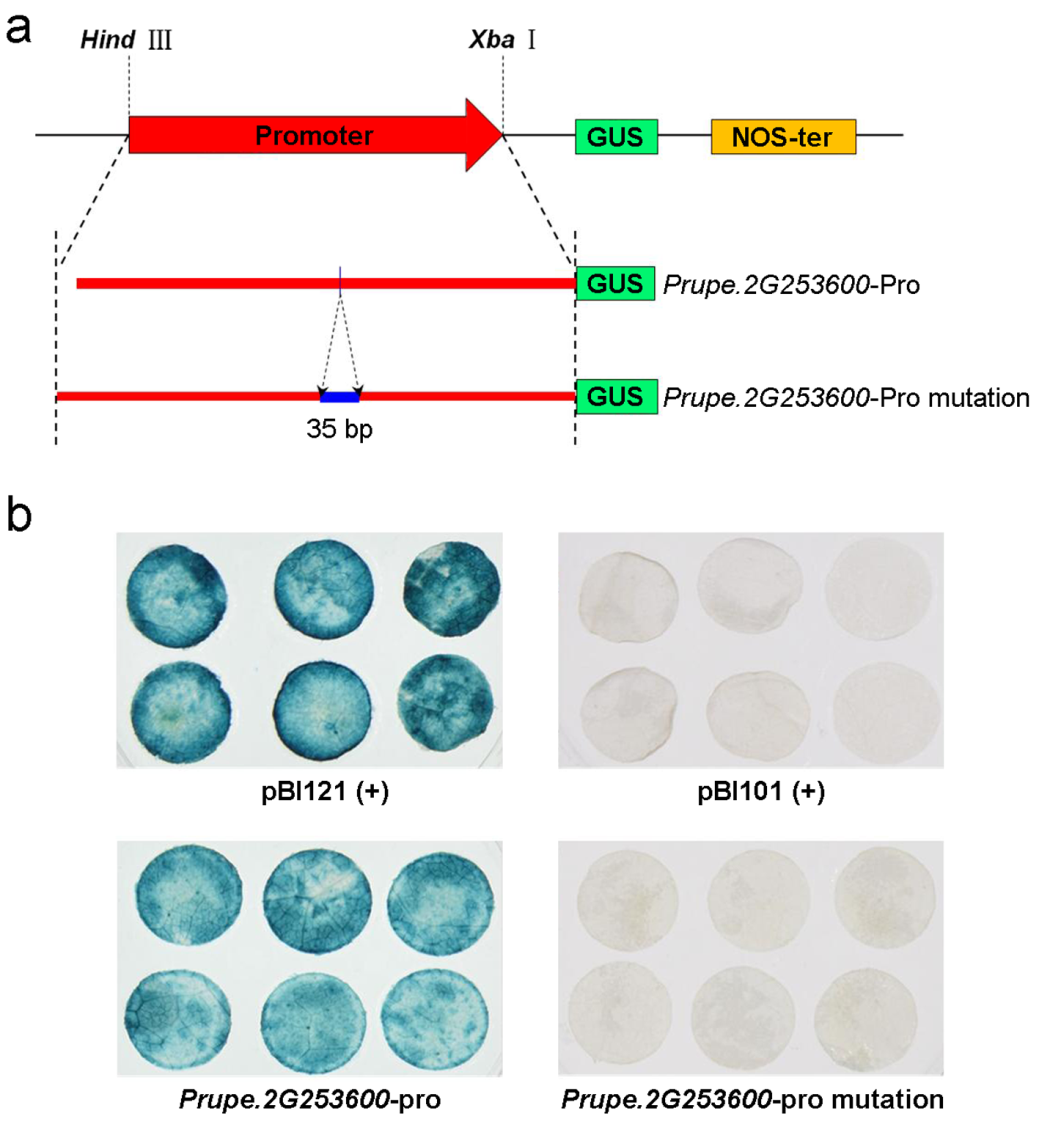
**

**Supplementary Figure 19 The vector construction (a) and promoter activity assay (b) of *Prupe.2G253600* promoter which recovered 35 bp deletion in ‘Hong Gen Gan Su Tao 1#’ peach.**

**Supplementary Figure 20 The altitude distribution of accessions used to identify altitude adaptability related genes for genome resequencing.** In the study, a total of 49 accessions were used. From the figure, the intermediate value is about 3200-3400 m. Then, low altitude and high altitude accessions can be divided according to this value.


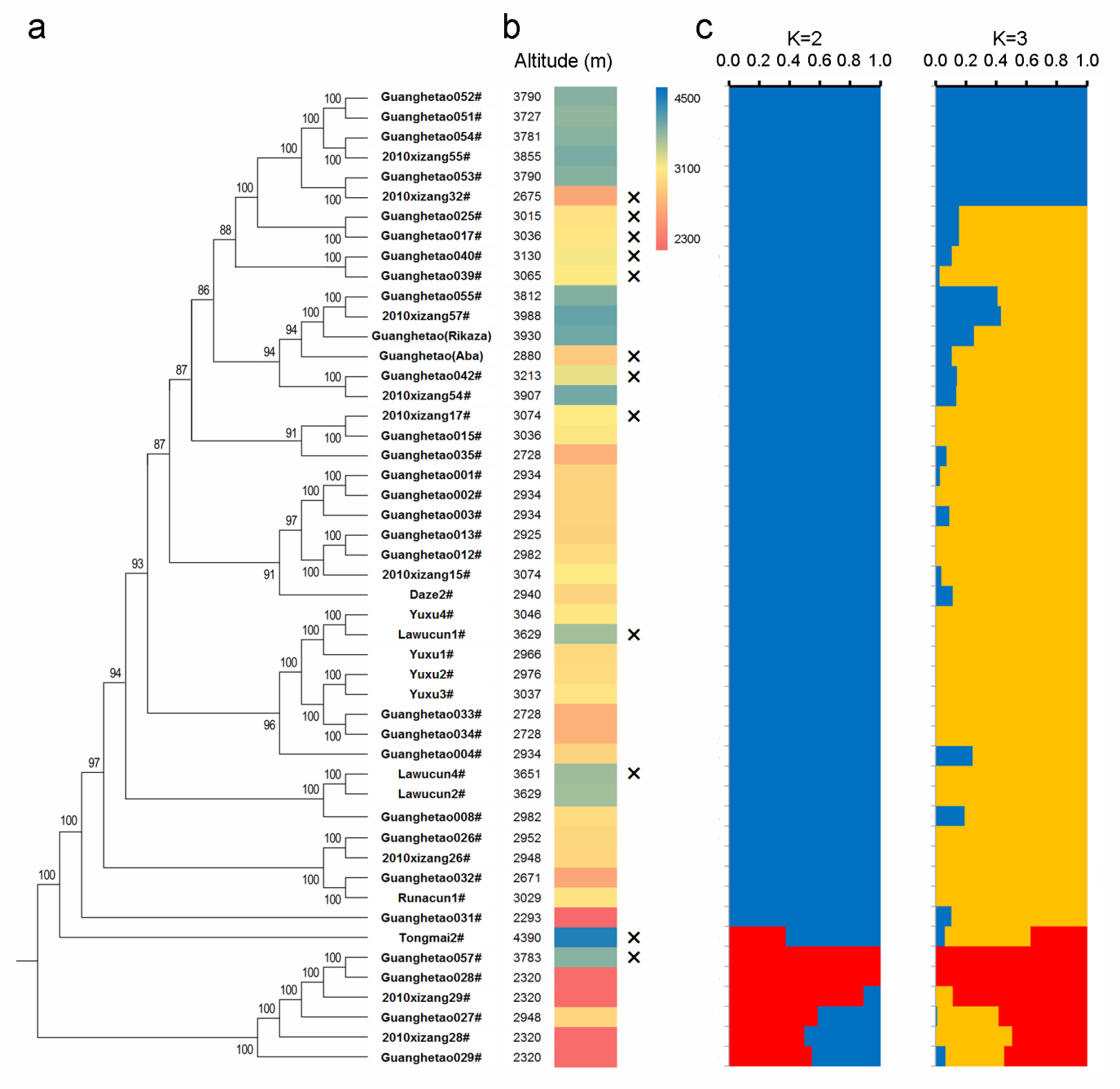


**Supplementary Figure 21 Phylogenetic tree of 49 accessions of *P. mira* (a) originating from regions with different altitudes (b) and the population structure (c) when K=2 and 3.** According to the phylogenetic tree and STRUCTURE analysis, the accessions thought to have not corresponded to its altitude categories or reckoned as an admixture subgroup between high and low altitude subgroups were labelled with multiple sign and removed in the following analysis.

**
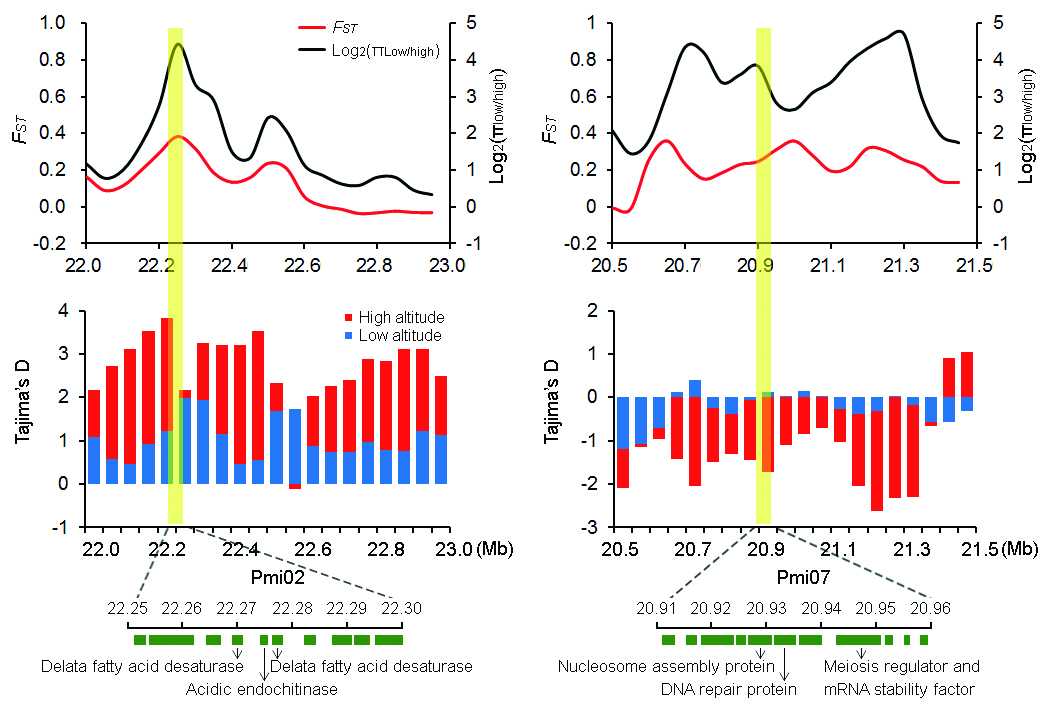
**

**Supplementary Figure 22 Two genome regions associated with high-altitude adaptation.** We also detected two genomic regions showing strong reduction of diversity and high differentiation. In the first region spanning from 22.25 Mb to 22.30 Mb of Chr. 2, *Pmi02g2009* and *Pmi02g2011* encoded protein delta (8)-fatty-acid desaturase and *Pmi02g2010* encoded acidic endochitinase were involved in cold response. And in another region (Chr. 7: 20.91..20.96 Mb), *Pmi07g2344* encoded nucleosome assembly protein, *Pmi07g2345* encoded DNA repair protein recA homolog, *Pmi07g2347* encoded meiosis regulator and mRNA stability factor were involved in DNA repair. The functions of above genes were referred to the KEGG annotation results on uniprot database (www.uniprot.org/).


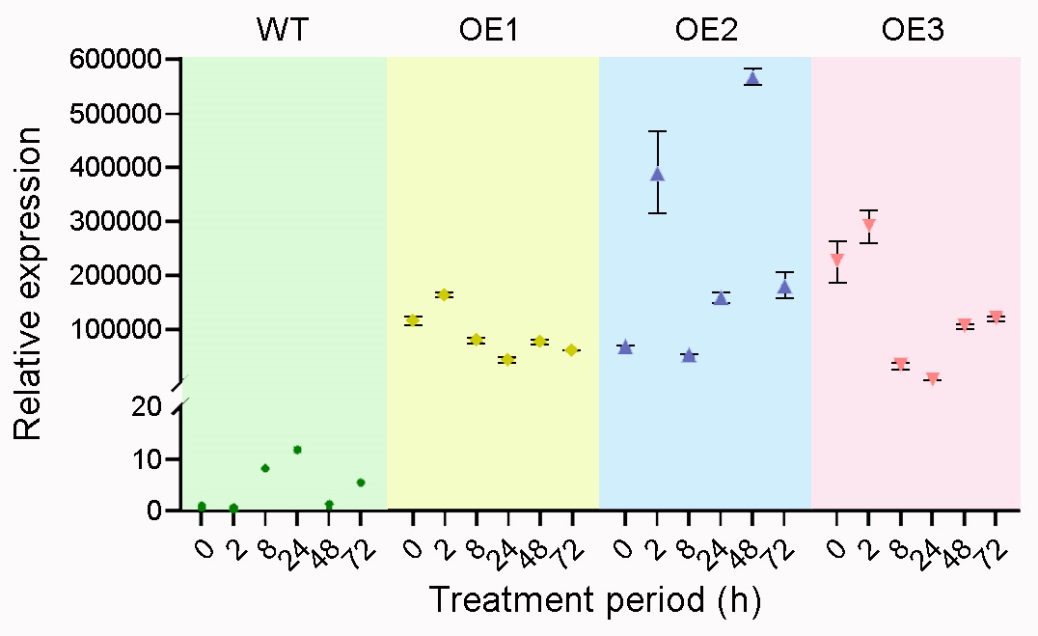


**Supplementary Figure 23 The gene expression of wild-type (WT) and three transgenic plants (OE1-OE3) in Arabidopsis which induced by 4 ℃ for different period.**

**Supplementary Figure 24 The proline content of wild-type (WT) and transgenic plant (OE) in Arabidopsis which induced by 4 ℃ for 24 and 48 days.** We found that the proline contents at 24 and 48 hours of cold induction were higher than 0 hour. Moreover, the increase in the transgenic plants was significantly higher than that in wild type ones.


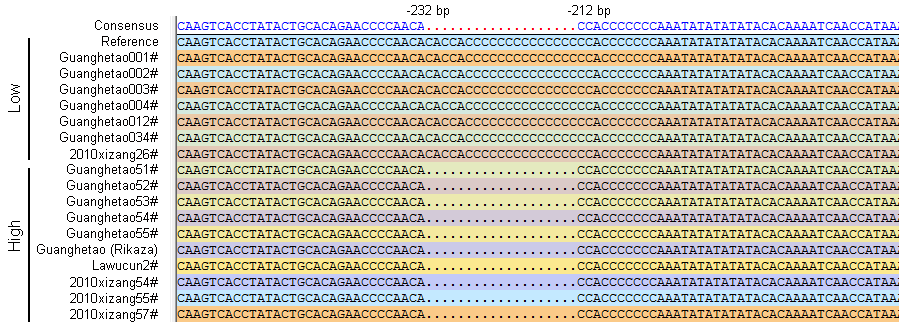


**Supplementary Figure 25 A deletion identified in the promoter of *Pmi02g3025* gene in varieties with different altitude.**
